# Co-option and neofunctionalization of stomatal executors for defence against herbivores in Brassicales

**DOI:** 10.1038/s41477-025-01921-1

**Published:** 2025-02-24

**Authors:** Makoto Shirakawa, Tomoki Oguro, Shigeo S. Sugano, Shohei Yamaoka, Mayu Sagara, Mai Tanida, Kyoko Sunuma, Takuya Iwami, Tatsuyoshi Nakanishi, Keita Horiuchi, Kie Kumaishi, Soma Yoshida, Mutsumi Watanabe, Takayuki Tohge, Takamasa Suzuki, Yasunori Ichihashi, Atsushi Takemiya, Nobutoshi Yamaguchi, Takayuki Kohchi, Toshiro Ito

**Affiliations:** 1 Graduate School of Science and Technology, Nara Institute of Science and Technology, Ikoma, Japan; 2 Precursory Research for Embryonic Science and Technology, Japan Science and Technology Agency, Kawaguchi-shi, Japan; 3 Bioproduction Research Institute, National Institute of Advanced Industrial Science and Technology, Tsukuba, Japan; 4 Graduate School of Biostudies, Kyoto University, Kyoto, Japan; 5 RIKEN BioResource Research Center, Tsukuba, Japan; 6 Graduate School of Sciences and Technology for Innovation, Yamaguchi University, Yamaguchi, Japan; 7 Department of Biological Chemistry, College of Bioscience and Biotechnology, Chubu University, Kasugai, Japan

**Keywords:** Cell fate, Stomata

## Abstract

Co-option of gene regulatory networks leads to the acquisition of new cell types and tissues. Stomata, valves formed by guard cells (GCs), are present in most land plants and regulate CO_2_ exchange. The transcription factor (TF) FAMA globally regulates GC differentiation. In the Brassicales, FAMA also promotes the development of idioblast myrosin cells (MCs), another type of specialized cell along the vasculature essential for Brassicales-specific chemical defences. Here we show that in *Arabidopsis thaliana*, FAMA directly induces the TF gene *WASABI MAKER* (*WSB*), which triggers MC differentiation. *WSB* and *STOMATAL CARPENTER 1* (*SCAP1*, a stomatal lineage-specific direct FAMA target), synergistically promote GC differentiation. *wsb* mutants lacked MCs and the *wsb scap1* double mutant lacked normal GCs. Evolutionary analyses revealed that WSB is conserved across stomatous angiosperms. We propose that the conserved and reduced transcriptional FAMA–*WSB* module was co-opted before evolving to induce MC differentiation.

## Abstract (web summary)

The authors identified a transcriptional module, FAMA–WASABI MAKER (WSB), for the development of stomata and idioblast myrosin cells. They propose that the conserved and reduced FAMA–WSB module was co-opted before evolving to induce idioblast development.

## Main

During eukaryotic evolution, the co-option of existing gene regulatory networks (GRNs) controlled by transcription factors (TFs) has been associated with the acquisition of new cell types, tissues and organs^1–4^. Basic helix-loop-helix (bHLH)-type TFs play key roles in cell fate determination and cell differentiation during eukaryotic development^5–7^.

In plants, three sister bHLH TFs (SPEECHLESS [SPCH], MUTE and FAMA) promote the differentiation of stomata in epidermal tissues^8–13^. Stomata are valves through which CO_2_ passes, placing them at the centre of the global carbon cycle; stomata are composed of a pair of specialized cells, the guard cells (GCs)^8–13^. SPCH, MUTE and FAMA form heterodimers with other bHLH-type TFs, namely, SCREAM (SCRM, also reported as INDUCER OF CBF EXPRESSION 1 (ICE1)) and SCRM2, and control the transition from protodermal cells to meristemoids, from meristemoids to guard mother cells (GMCs), or from GMCs to GCs^14–17^. Recently, it was reported that MUTE–SCRM and FAMA–SCRM heterodimers have the potential for pioneering activity (that is, as pioneer TFs), binding their target genes even when ensconced in closed or open local chromatin, and they initiate remodelling of the epigenome^18^. Although early stomatal development is well understood, GC differentiation remains enigmatic^19^. Indeed, how FAMA regulates the differentiation of GCs through its direct targets is largely unknown.

Two TF genes, *ETHYLENE-RESPONSE FACTOR 51* (*ERF51*, also reported as *DEHYDRATION-RESPONSIVE ELEMENT-BINDING PROTEIN 2F* (*DREB2F*))^20,21^ and *STOMATAL CARPENTER 1* (*SCAP1*)^20,22,23^, are potential downstream factors of FAMA because the expression of *ERF51* and *SCAP1* is upregulated in an oestrogen-inducible *FAMA* overexpression line^20^. In addition, *ERF51* is expressed in the stomatal lineages and FAMA directly binds to the *WSB* promoter region in *FAMA* overexpression lines^20^. However, a previous study reported that an *erf49 erf50 erf51 erf52* quadruple mutant did not exhibit highly penetrant stomatal phenotypes^20^. Therefore, the physiological function of ERF51 in stomatal development remains largely unknown. Independent of the study by ref. ^20^, *SCAP1* was isolated as the causal mutated gene in a mutant impaired in CO_2_-dependent changes of stomatal conductance and is specifically expressed in GCs^22^. Although a subset of *scap1* GCs showed an abnormal morphology (the ventral cell walls of GCs appeared floppy and were often irregularly curved), the stomatal phenotypes of *scap1* mutants were much weaker than those of *fama* mutants^22^. *SCAP1* is probably a direct target of FAMA; however, other direct targets of FAMA may synergistically promote the differentiation of GCs together with SCAP1 (ref. ^24^).

We and another group previously reported that FAMA regulates the differentiation of another type of specialized cells called idioblast myrosin cells (MCs), which are distributed along the vasculature in inner tissues^25–29^. MCs play a critical role in a Brassicales-specific chemical defence system called the myrosinase-glucosinolate system, in which MCs are thought to protect the vasculature from herbivory attacks^27–29^. When herbivores damage plant tissues, myrosinase (also named thioglucoside glucohydrolase) and its substrate, glucosinolate, are released from MCs and S cells (a glucosinolate-rich cell type), respectively, and they react to produce the toxic compounds isothiocyanates^27–29^. Humans also detect isothiocyanates as pungency in some foods (such as wasabi and horseradish)^30^. Despite the importance of the myrosinase-glucosinolate system as a plant defence mechanism, details about the downstream targets of FAMA involved in the development of MCs, and how Brassicales plants acquired this defence system (MCs and S cells) during evolution are largely unknown^27–29^.

We previously hypothesized that FAMA-mediated GRNs were co-opted from GCs to MCs during evolution, as GCs are one of the oldest innovations of land plants, whereas MCs are a Brassicales innovation^29,31,32^. However, the precise components of the GRNs regulating GC and MC development via FAMA, and how each cell identity is differentially regulated are largely unknown, as are the key genes directly regulated by FAMA.

Here we describe two direct FAMA target genes that encode TFs: ERF51, which we renamed WASABI MAKER (WSB) after its functional analysis, and SCAP1, in *Arabidopsis thaliana* (hereafter, Arabidopsis). Genetic and biochemical analyses revealed that FAMA deploys *WSB* and *SCAP1* for GC differentiation, and *WSB* specifically for MC differentiation. We propose that the FAMA–*WSB–SCAP1* GRN had an established function in the differentiation of GCs before being co-opted and reduced to the FAMA–*WSB* GRN for the differentiation of MCs during evolution, reflecting the neofunctionalization of FAMA and WSB. This study not only advances our understanding of how specialized cells arise during evolution, but also provides insight into the co-option and neofunctionalization of GRNs during evolution.

## Results

### Comprehensive atlas of genes regulated by pioneer TF FAMA

**Fig. 1:**
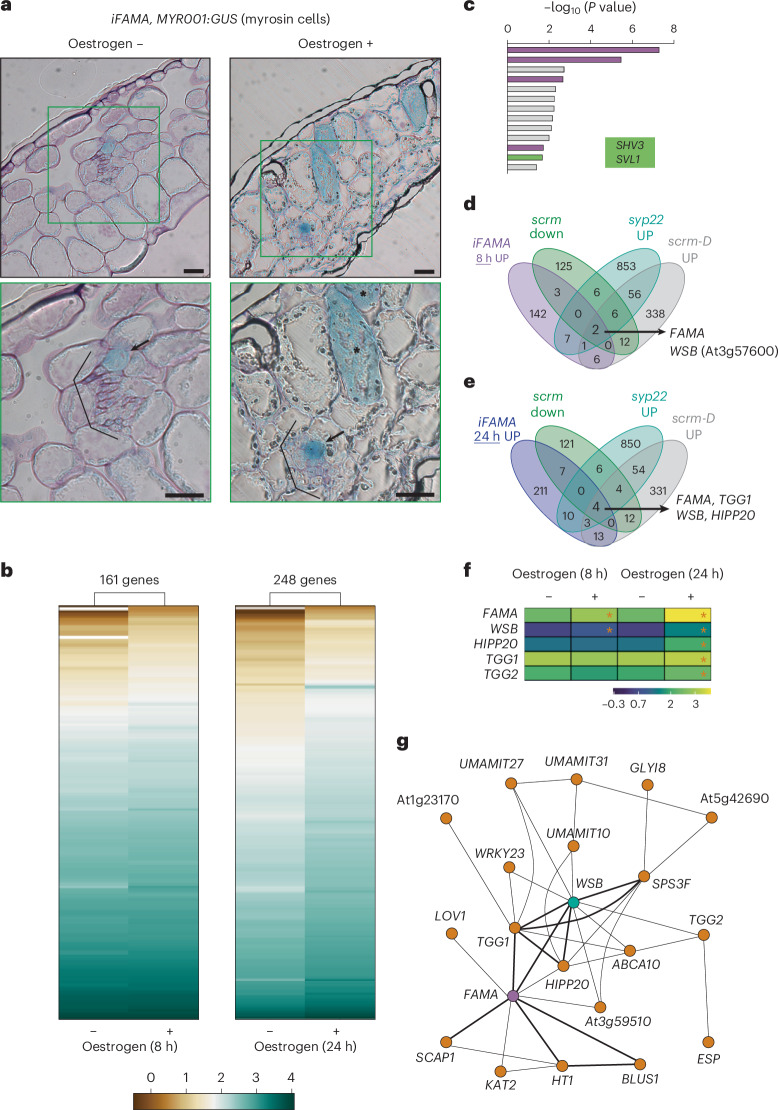
The TF gene *WSB* is a direct target of FAMA based on combinational RNA-seq analyses. **a**, Ectopic expression of the idioblast myrosin cell (MC) marker *MYR001* in *iFAMA* transgenic lines, as shown in cross sections of leaves from *iFAMA* transgenic seedlings 4 days after germination (DAG) with (+) or without (−) oestrogen treatment (10 µM oestrogen) for 8 h. Boxed areas in upper panels are enlarged in lower panels. Black brackets, vascular bundles; arrows, MCs along the vasculature; asterisks, ectopic MCs. Scale bars, 20 µm. **b**, *k*-means clustering analysis of differentially expressed genes between *iFAMA* seedlings at 4 DAG with or without oestrogen treatment (10 µM oestrogen) for 8 h (left) or 24 h (right). Color bar indicates the expression levels (log_2_-fold change, ppm). Gene lists used in this panel are given in Supplementary Data 1 and 2. **c**, GO term enrichment analysis of 161 genes of *iFAMA* 8 h UP (*P* < 0.05). The *P* values for GO analysis were obtained by converting the *Z*-scores on the basis of a two-tailed *Z*-test. Cell wall categories are highlighted in purple; the stomatal complex morphogenesis category including *SHV3* and *SVL1* is highlighted in green. The names of genes belonging to each GO category are listed in Supplementary Data 3. **d**, Venn diagram showing the extent of overlap between *iFAMA* 8 h UP (light purple), *scrm* DOWN (green), *syp22* UP (cyan) and *scrm-D* UP (grey) differentially expressed genes. *FAMA* and *WSB* (At3g57600) are the only two common genes. **e**, Venn diagram showing the extent of overlap between *iFAMA* 24 h UP (deep purple), *scrm* DOWN (green), *syp22* UP (cyan) and *scrm-D* UP (grey). *FAMA*, *WSB*, *TGG1* and *HIPP20* are the four common genes. **f**, Heat map representation of the expression levels of *FAMA*, *WSB*, *HIPP20*, *TGG1* and *TGG2*. Color bar indicates the expression levels (log_2_-fold change, ppm). Asterisks indicate significant differences between + and − oestrogen treatment (10 µM oestrogen) at the same time point (FDR < 0.05). **g**, Co-expression network of *WSB* based on the Arabidopsis ATTED-II transcriptome database^40^. Lines indicate connections between genes. Note that *WSB* (green) is the only gene directly connected to *FAMA* (purple), *TGG1* and *TGG2*. The only TF genes that are strongly connected with *FAMA* (as shown by thicker lines) are *WSB* and *SCAP1*.

**Extended Data Fig. 1:**
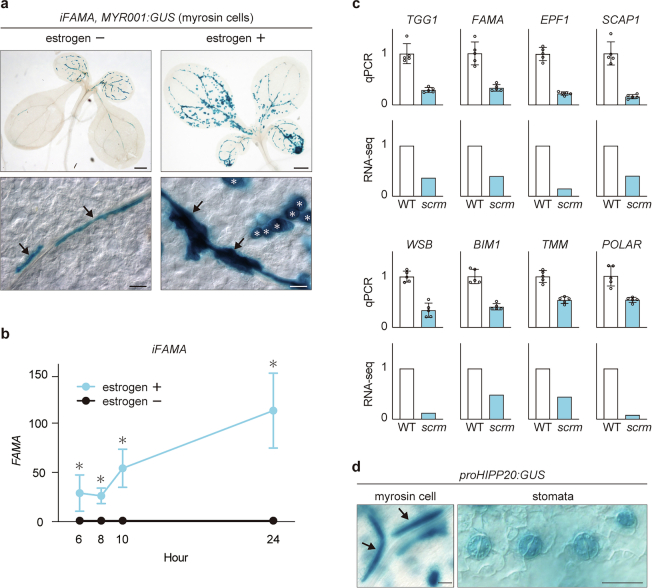
Identification of downstream targets of FAMA. **a**, Ectopic expression of an MC marker is induced by the transient overexpression of *FAMA*. Representative GUS staining pattern of leaves from *iFAMA* transgenic seedlings at 4 DAG with (+) or without (−) 8 h of estrogen treatment (10 µM). MCs were visualized with the reporter *MYR001:GUS*. In the lower panels, arrows indicate MCs next to vascular cells, and asterisks indicate ectopic MCs. **b**, Time-course analysis of *FAMA* expression levels in *iFAMA* seedlings at 4 DAG with (+) or without (−) estrogen treatment (10 µM; 6, 8, 10, or 24 h) (*n* = 5). Data are means ± standard deviation (SD). A two-tailed unpaired Student’s *t*-test was used to calculate the *p*-values between estrogen treatments. *, *p* < 0.05. **c**, Validation of RNA-seq data from *scrm*. The elongating top 1 cm of the stem without flowers was collected at 25–30 DAG from WT and *scrm* (*n* = 5). The expression data of eight marker genes were determined by RT-qPCR (upper) and compared to the results obtained by RNA-seq (lower) using the same RNA samples from the WT and *scrm*. RT-qPCR experiments were performed five times, and open circles represent individual data points. Data are means ± SD. Note that the expression levels of all eight genes in *scrm* were significantly downregulated (A two-tailed Student’s *t*-test was used to calculate the *p*-values between WT and *scrm*. *, *p* < 0.05.) compared to the WT. Consistent with the RT-qPCR analysis, the same eight marker genes were also significantly downregulated in *scrm* compared to the WT (false discovery rate [FDR] < 0.05). **d**, GUS staining of a rosette leaf from WT Arabidopsis (Col-0) seedlings at 14 DAG harboring *proHIPP20:GUS*. Enlarged image of myrosin lineage cells (left) and epidermal stomatal lineage cells (right) are shown. Arrows indicate MCs along veins. Scale bars, 500 µm (**a**, upper), 50 µm (**a**, lower), and 20 µm (**d**).

To obtain a nearly complete map of transcriptional changes driven by FAMA, we employed an oestrogen-inducible *FAMA* overexpression line (*iFAMA* for induced *FAMA* expression), which was previously used to show that FAMA is sufficient for the differentiation of MCs and GCs^14,20,26^. Oestrogen treatment was reported to suffice for GC induction^14,20^. Indeed, after 8 h of oestrogen treatment, we detected ectopic expression of the MC marker gene *MYR001* (ref. ^33^) (consisting of the *VACUOLAR SORTING RECEPTOR HOMOLOG 1* (*VSR1*) promoter driving the reporter gene *β-GLUCURONIDASE* (*GUS*)) in *iFAMA* outside the area adjacent to the vasculature where MCs normally develop. This result suggested that MC characteristics were conferred to mesophyll cells by *FAMA* expression (Fig. 1a and Extended Data Fig. 1a) and indicated that 8 h of *FAMA* induction is sufficient to trigger FAMA-mediated downstream transcriptional changes that promote MC differentiation. A time-course expression analysis of oestrogen-treated *iFAMA* seedlings revealed a rapid upregulation of *FAMA* transcript levels relative to seedlings mock treated with diluted ethanol (−oestrogen), by >25-fold within 8 h and >100-fold after 24 h (Extended Data Fig. 1b). Transcriptome deep sequencing (RNA-seq) analysis of *iFAMA* mock-treated or oestrogen-treated seedlings identified 161 and 248 upregulated and 418 and 189 downregulated genes in *iFAMA* after 8 and 24 h of oestrogen treatment, respectively (hereafter referred to as *iFAMA* 8 h UP or DOWN and *iFAMA* 24 h UP or DOWN) compared with mock-treated *iFAMA* seedlings (with a false discovery rate (FDR) < 0.05) (Fig. 1b, and Supplementary Data 1 and 2). At the 24-h time point, more genes were upregulated and to a much greater extent than at 8 h (Fig. 1b). The Gene Ontology (GO) categories enriched for *iFAMA* 8 h UP genes were overwhelmingly related to the cell wall, suggesting that FAMA regulates cell wall modifications during the differentiation of GCs and MCs (Fig. 1c purple and Supplementary Data 3). Highly enriched categories also included the term ‘stomatal complex morphogenesis’ (Fig. 1c green and Supplementary Data 3). Strikingly, two genes associated with this category, *SHAVEN3* (*SHV3*) and *SHV3*-*LIKE 1* (*SVL1*), are redundantly required for GC morphogenesis^34^. Thus, we successfully captured the transcriptional changes driven by FAMA in *iFAMA* 8 h UP, which might include key genes for the differentiation of MCs and GCs.

### Identification of the TF gene *WSB*, a primary target of FAMA

To identify key genes for the differentiation of MCs and GCs from the 161 genes regulated by FAMA, we focused on SCRMs, which interact with FAMA to promote the differentiation of MCs and GCs^17,26^. The *fama* and *scrm scrm2* mutants exhibited severe dwarfism phenotypes^14,17^. We reasoned that it might be difficult to collect tissue samples with similar shapes, including similar cell types, from the wild type (WT) and these mutants due to this large size difference; an RNA-seq analysis using these mutants would probably mainly reflect the differences in cell types in the collected samples rather than a specific differential gene expression caused by loss of FAMA–SCRM complexes. Therefore, we used *scrm* single mutants which did not exhibit severe dwarfism and showed the low accumulation levels of the myrosinase, THIOGLUCOSIDE GLUCOHYDROLASE 1 (TGG1) in leaves and stems^17,26^. In addition, we performed RNA-seq analysis on stems, as they offered samples with no morphological differences between WT and *scrm* although *scrm* leaves were slightly smaller than those of WT, thus allowing us to collect samples at similar developmental stages and similar size. The loss of SCRM function was associated with the downregulation of 154 genes (*scrm* DOWN), including known marker genes for both GCs and MCs (Extended Data Fig. 1c and Supplementary Data 4). To identify the downstream targets of FAMA that are expressed in both GCs and MCs, we included two additional published transcriptome datasets: (1) genes upregulated in the semi-dominant mutant *scrm-D* (*scrm-D* UP), in which mature stomata are overproduced^17,35^; and (2) genes upregulated in the *syntaxin of plants 22* (*syp22*) mutant (*syp22* UP), in which mature MCs are overproduced^36–39^ (Supplementary Data 5).

The overlap between these four datasets comprised only two genes with *iFAMA* 8 h UP: *FAMA* itself and At3g57600 (Fig. 1d and Supplementary Data 5). We also detected only four genes overlapping with *iFAMA* 24 h UP: *FAMA*, At3g57600, *TGG1* (refs. ^36,40^) and *HEAVY METAL ASSOCIATED ISOPRENYLATED PLANT PROTEIN 20* (*HIPP20*)^41^ (Fig. 1e and Supplementary Data 5). We selected At3g57600, which we named *WASABI MAKER* (*WSB*) after functional analysis (see below), as it was upregulated before *HIPP20* and the myrosinase genes *TGG1* and *TGG2* (Fig. 1f), which are expressed in both GCs and MCs (Extended Data Fig. 1d)^40^. Moreover, *WSB* was the only gene co-expressed with *FAMA*, *TGG1* and *TGG2* in the ATTED-II database^42^ (Fig. 1g); the overlap between the four *iFAMA* transcriptome datasets from this study and the transcriptome dataset from ref. ^20^ consisted of only two genes, *FAMA* and *WSB* (Supplementary Data 6). *WSB* encodes an APETALA2/ERF (AP2/ERF)-type TF belonging to Group IV of this family and known as ERF51 and DREB2F, with no known biological function^43^.

### *WSB* is a direct target of the FAMA–SCRM complexes

**Fig. 2:**
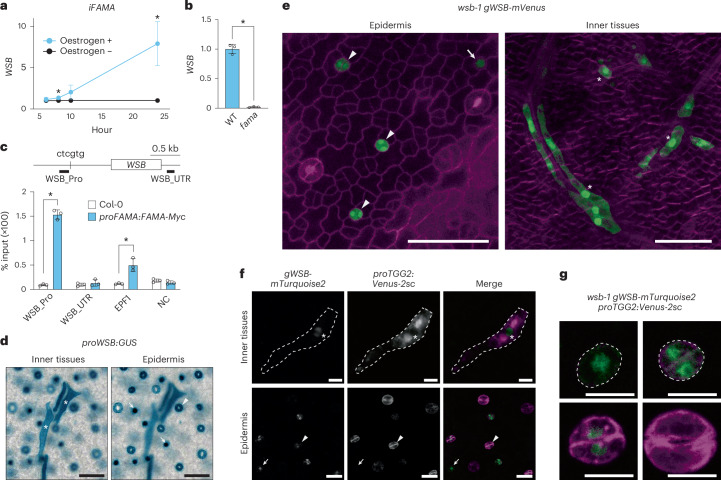
*WSB* is expressed in MCs and GCs. **a**, Time-course analysis of *WSB* expression levels in seedlings at 4 DAG for *iFAMA* lines with or without oestrogen treatment (10 µM oestrogen for 6, 8, 10 or 24 h) (*n* = 5; relative expression levels) (means ± s.d.). A two-tailed unpaired Student’s *t*-test was used to calculate the *P* values between + and − oestrogen treatments. *P < 0.05. **b**, RT–qPCR analysis of *WSB* transcript levels in whole seedlings at 14 DAG for WT (Col-0) and *fama* (*n* = 3; relative expression levels). Open circles represent individual data points. Data are means ± s.d. A two-tailed unpaired Student’s *t*-test was used to calculate the *P* values between WT and *fama*. *P < 0.05. **c**, ChIP–qPCR assay testing the binding of FAMA to the *WSB* locus (*n* = 3; % input). The percentage of input of FAMA-Myc at *WSB* was analysed using *fama proFAMA:FAMA-Myc* seedlings at 4 DAG. Open circles represent the percentage of input from each sample. Data are means ± s.d. A two-tailed unpaired Student’s *t*-test was used to calculate the *P* values between *proFAMA:FAMA-Myc* and Col-0. *P < 0.05. *EPF1* served as a positive control. NC, negative control. **d**, GUS staining of true leaves from WT Arabidopsis (Col-0) harbouring the *proWSB:GUS* reporter at 14 DAG. The two panels are photographs of the same region in different focal planes. Left: photograph focusing on inner tissues, especially vascular tissues; asterisks, elongated MCs. Right: photograph focusing on the epidermis; arrows, GMCs; arrowhead, GC. **e**, Left: confocal image of epidermis from first or second true leaves of *wsb-1 gWSB-mVenus* at 5 DAG. Signals of WSB-mVenus are shown in green. Cell outlines were visualized by propidium iodide staining (magenta). Arrow, GMC; arrowheads, GCs. Right: confocal image of inner tissues from a fifth true leaf of *wsb-1 gWSB-mVenus* at 8 DAG. Signals of WSB-mVenus are shown in green. Cell outlines were visualized by SR2200 staining (magenta). Asterisks, MCs. **f**, Confocal images of myrosin (top) and stomatal (bottom) lineage cells in *wsb-1 gWSB-mTurquoise2 proTGG2:Venus-2sc* true leaves at 7 DAG. Asterisks, elongating MCs; arrows, GMCs; arrowheads, GCs. 2sc (C-terminal peptides of 2S albumin) is a vacuolar sorting signal. **g**, Enlarged images of stomatal-lineage cells from **f** (top left to right: GMC, GC just after division; bottom left to right: developing GC, mature GC). Scale bars, 50 µm (**d**,**e**), 20 µm (**f**) and 10 µm (**g**).

To examine whether FAMA is required for and directly regulates *WSB* expression, we determined the expression levels of *WSB* in *iFAMA* in response to oestrogen treatment using reverse-transcription quantitative PCR (RT–qPCR). Consistent with the RNA-seq data, *WSB* expression was rapidly induced after 8 h of oestrogen treatment and reached a >7-fold increase after 24 h (Fig. 2a). Conversely, we failed to detect *WSB* transcripts in *fama* mutants (Fig. 2b) and detected no GUS signal from the leaves of *fama proWSB:GUS* seedlings (Extended Data Fig. 3d). These results suggest that FAMA is necessary and sufficient for *WSB* expression.

**Extended Data Fig. 2:**
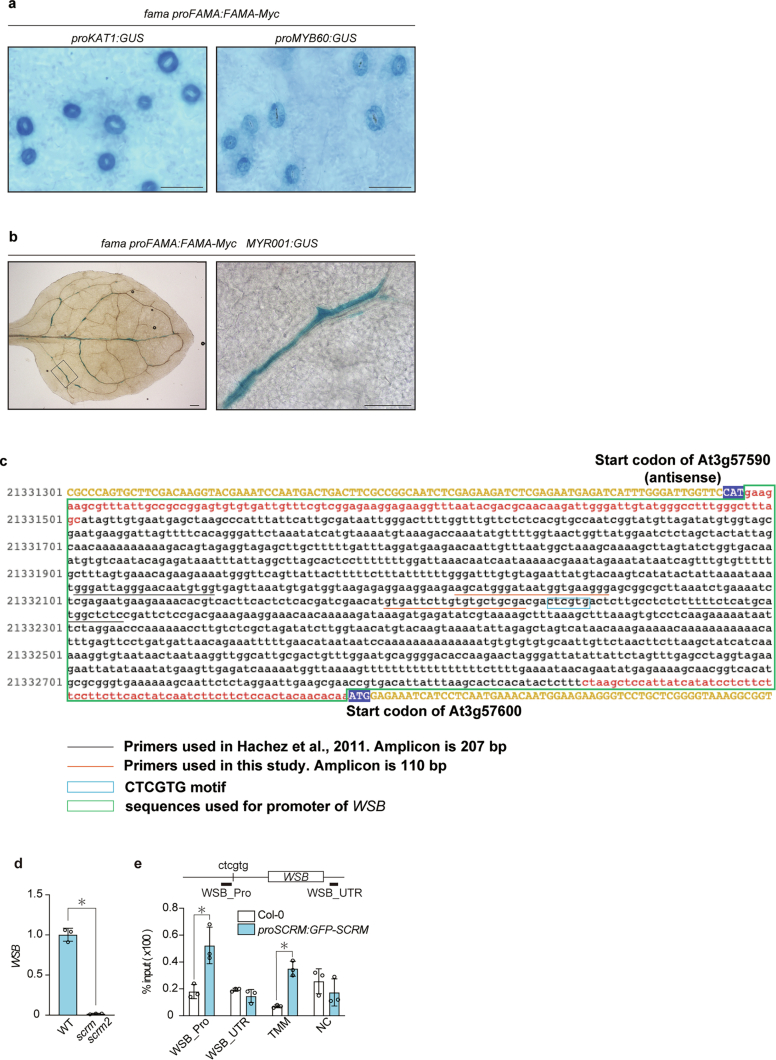
FAMA and SCRM directly activate *WSB* expression. **a**, Left, GUS staining of true leaves from *fama* seedlings harboring *proFAMA:FAMA-Myc* and *proKAT1:GUS* at 14 DAG. Right, GUS staining of true leaves from *fama* seedlings harboring *proFAMA:FAMA-Myc* and *proMYB60:GUS* at 14 DAG. Note that *proFAMA:FAMA-Myc* complemented the *fama* mutant phenotypes and induced the expression of the mature GC markers *KAT1* and *MYB60*. **b**, Left, GUS staining of true leaves from *fama* seedlings harboring *proFAMA:FAMA-Myc* and *proVSR1:GUS* at 14 DAG. Right, an enlarged image of boxed area in left. Note that *proFAMA:FAMA-Myc* complemented *fama* mutant phenotypes and induced the expression of the MC marker *MYR001/VSR1*. **c**, Sequence of the *WSB* promoter showing the position of primers used in this study and by ref. ^20^ for the ChIP experiments. **d**, RT-qPCR analysis of *WSB* transcript levels in whole seedlings at 14 DAG for the WT and *scrm scrm2*. Experiments were performed three times, and open circles represent individual data points. Data are means ± SD. A two-tailed Student’s *t*-test was used to calculate the *p*-values between WT and *scrm scrm2*. *, *p* < 0.05. **e**, ChIP-qPCR binding assay showing the binding of SCRM to the *WSB* locus (*n* = 3; the percentage of input). The percentage of input (% input) of GFP-SCRM at the *WSB* was analyzed using *proSCRM:GFP-SCRM* seedlings at 4 DAG. Whole seedlings were used as samples for the extraction of DNA-protein complexes. Open circles represent the percentage of input from each sample. Data are means ± SD. A two-tailed unpaired Student’s *t*-test was used to calculate the *p*-values between *proSCRM:GFP-SCRM* and Col-0. *, *p* < 0.05. The *TMM* locus served as a positive control; NC, negative control. Scale bars, 100 µm (**b**, left) and 50 µm (**a** and **b**, right).

**Extended Data Fig. 3:**
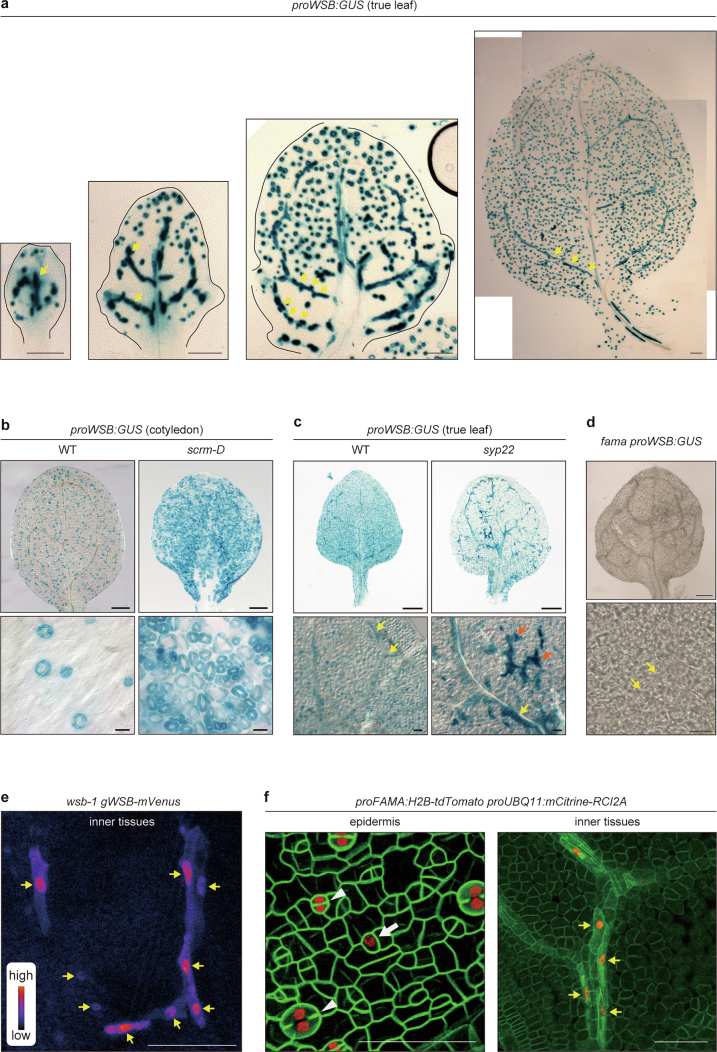
*WSB* is expressed in MCs and GCs throughout leaf development. **a**, Change in *proWSB:GUS* staining pattern as a function of development. True leaves from *proWSB:GUS* at 14 DAG were used for GUS staining. The leaves were arranged from young (higher-order leaves) to old (lower-order leaves). Throughout leaf development, GUS signals were observed as small dots (GCs) and elongated cells along leaf veins (MCs, yellow arrows). Note that these staining patterns are very similar to those observed for *proFAMA:GUS*^14,25,26^. **b**, GUS staining of cotyledons from WT and *scrm-D* seedlings at 14 DAG harboring the *proWSB:GUS* reporter. Image of whole leaves (upper); enlarged images of epidermis (lower). Note that in *scrm-D*, the entire epidermis differentiates into GCs with blue signals (GUS). **c**, GUS staining of rosette leaves from WT and *syp22* seedlings at 14 DAG harboring *proWSB:GUS*. Images of whole leaves (upper); enlarged images of inner tissues (lower). Yellow arrows, MCs along veins; orange arrows, clusters of MCs. **d**, *WSB* expression depends on FAMA. GUS staining of a rosette leaf from *fama-1 proWSB:GUS* seedling at 14 DAG. Whole leaf (upper), enlarged image of inner tissues (lower). Yellow arrows, veins. Note that no blue signal (GUS) was observed in the leaves. **e**, WSB accumulates primarily in the nucleus of myrosin lineage cells. Confocal Z-stack image of a rosette leaf from *wsb-1* g*WSB-mVenus* seedling at 8 DAG. mVenus signal intensities are shown in blue to red according to increasing intensity levels. Yellow arrows, nucleus of myrosin lineage cells. **f**, *FAMA* is expressed in GMCs, GCs, and MCs. Confocal Z-stack image of a rosette leaf from a seedling at 8 DAG carrying *proFAMA:H2B-tdTomato* (red; nucleus) and *proUBQ11:mCitrine-RCI2A* (green; plasma membrane). Samples were cleared with ClearSee. Left panel, white arrows indicate GMCs, and white arrowheads indicate GCs in the epidermis. Right panel, yellow arrows indicate MCs in inner tissues. Scale bars, 100 µm (**a**), 500 µm (**b** and **c**, upper), 50 µm (**b** and **c**, lower; **e** and **f**), 200 µm (**d**, upper), and 20 µm (**d**, lower).

FAMA was reported to directly bind to the *WSB* promoter region in chromatin immunoprecipitation–quantitative PCR (ChIP–qPCR) assays using *FAMA* overexpression lines^20^. We thus asked whether FAMA directly binds to the *WSB* promoter region in GCs and MCs by performing a ChIP–qPCR analysis using a complementation line, *fama proFAMA:FAMA-myc*^44^, in which MC-specific reporters and GC-specific reporters were expressed properly (Extended Data Fig. 2a,b), with an anti-myc antibody (Fig. 2c). Indeed, we detected a significant increase in FAMA binding to a *WSB* promoter region near a CTCGTG motif (-672 to -667 bp relative to the ATG) that is identical to the cognate *cis* element of the FAMA sister protein SPCH^45^ (Fig. 2c and Extended Data Fig. 2c). The value of % input (binding strength) to the *WSB* promoter was higher than that to the *EPIDERMAL PATTERNING FACTOR 1* (*EPF1*) locus to which binding of FAMA was previously shown^44^. These results suggest that FAMA directly binds to the *WSB* promoter in GCs and MCs. *WSB* expression was lower in *scrm* mutants (Extended Data Fig. 1c) and almost undetectable in the *scrm scrm2* double mutant (Extended Data Fig. 2d). In addition, SCRM-GFP (a fusion of SCRM to the green fluorescent protein)^17^ bound to the same region in the *WSB* promoter (Extended Data Fig. 2e). These results confirm that FAMA directly activates *WSB* expression (Fig. 2 and Extended Data Fig. 3d). Moreover, we determined that SCRM also directly activates *WSB* expression (Extended Data Figs. 1c and 2d,e). Collectively, these results suggest that FAMA–SCRM complexes are critical transcriptional activators for *WSB*.

### *WSB* is highly expressed in both MC and GC lineages

We investigated the spatial expression pattern of *WSB* in planta by generating transgenic lines expressing the *GUS* reporter gene under the control of a 1.4-kb *WSB* promoter fragment (*proWSB:GUS*). We observed GUS staining in elongated cells with horn-like extensions, that is, MCs, along the vasculature in the inner layer of leaves (Fig. 2d left); in small round cells, corresponding to GMCs; and in GCs in the leaf epidermis (Fig. 2d right), suggesting that *WSB* is expressed throughout leaf development in these specific cell types (Extended Data Fig. 3a). Consistent with the RNA-seq data (Fig. 1d,e), we noticed more GUS-positive cells in the epidermis of *scrm-D proWSB:GUS* seedlings (Extended Data Fig. 3b) and the inner layer of leaf tissue from *syp22 proWSB:GUS* seedlings than in the WT (Extended Data Fig. 3c).

We also investigated the accumulation of WSB with a translational fusion construct introduced in a *WSB* mutant (*wsb-1*; see below), *wsb-1 gWSB-mVenus*. In at least 10 independent transgenic lines, the *gWSB-mVenus* reporter (encoding a fusion between WSB and yellow fluorescent protein) was highly expressed in patches in leaf inner tissues and the epidermis, based on mVenus fluorescence patterns. We detected the accumulation of WSB in the nucleus of myrosin-lineage cells in inner tissues (Fig. 2e right and Extended Data Fig. 3e), as well as GMCs and young GCs in the epidermis (Fig. 2e left). We also observed weak WSB-mVenus signals in the cytoplasm of MCs and GCs.

The myrosinase gene *TGG2* is a marker for mature GCs and MCs. To clarify whether *WSB* is expressed in differentiating GCs and MCs, we compared the expression patterns of *WSB* and *TGG2*. Accordingly, we crossed the *proTGG2:Venus-2s*^46^ (encoding Venus targeted to vacuoles) transgenic line to *wsb-1* and transformed the resulting line with the *gWSB-mTurquoise2* construct (encoding WSB fused to cyan fluorescent protein^47^). We detected both fluorescent proteins in developing MCs and GCs, marking sites of Venus-2sc and WSB-mTurquoise2 accumulation (Fig. 2f). In the stomatal lineage, GMCs accumulated only WSB-mTurquoise2 (Fig. 2g). GCs just after division and young GCs presented both fluorescent markers, while mature GCs only accumulated Venus-2sc driven by the *TGG2* promoter. These results suggest that *WSB* is expressed before *TGG2* and that WSB specifically localizes to the nuclei of MCs, GMCs and young GCs. These results indicate that the expression of *WSB* follows the same pattern as that of its upstream regulator, *FAMA*, except in mature GCs^14,25,26^ (Extended Data Fig. 3f).

### Generation of *WSB* knockout mutants by CRISPR/Cas9

**Fig. 3:**
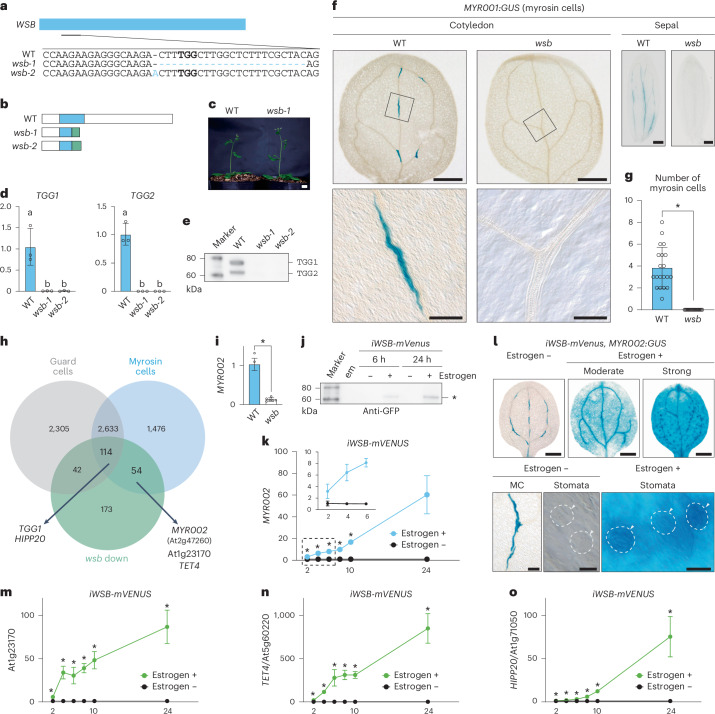
WSB is required for the development of MCs. **a**, Diagram of part of the *WSB* locus with the sgRNA target site and resulting mutations in the newly obtained CRISPR/Cas9-generated alleles, *wsb-1* and *wsb-2*. The protospacer adjacent motif (PAM) for Cas9 is highlighted in bold; deleted or inserted nucleotides are highlighted in blue. Blue box, exon. **b**, Diagram of WSB in the WT, *wsb-1* and *wsb-2*. Blue box, AP2 domain; green box, amino acids after the frameshift, with sequence different from the WT. **c**, The development of the *wsb-1* mutant and that of WT at 28 DAG are comparable. **d**, RT–qPCR analysis of relative expression levels of the myrosinase genes *TGG1* and *TGG2* in stems of the WT and *wsb* mutants (*n* = 3). The elongating top 1 cm of the stem without flowers was collected at 25–30 DAG. The relative expression levels were presented by setting the average expression level of *TGG1* or *TGG2* in the wild type to 1. Experiments were performed 3 times and open circles represent individual data points. Data are means ± s.d. Different lowercase letters indicate significant differences, as determined using one-way ANOVA followed by Tukey–Kramer test (*P* < 0.05). **e**, Immunoblot analysis of rosette leaves from the WT and *wsb* mutants at 14 DAG with anti-TGG1 and anti-TGG2 antibodies. Marker, a molecular weight marker. **f**, GUS staining of the cotyledons and sepals from WT and *wsb-3* plants harbouring the MC marker reporter *MYR001:GUS*. For cotyledons and sepals, seedlings at 2 DAG and flowers from plants at 25–30 DAG were used, respectively. Boxed area in top panel is enlarged at the bottom. **g**, Number of MCs (GUS-positive cells) in the cotyledons of WT and *wsb-3* seedlings (*n* = 20). Open circles represent the number of MCs from each sample. Data are means ± s.d. A two-tailed unpaired Student’s *t*-test was used to calculate the *P* values between the WT and *wsb*. *P < 0.05. **h**, Venn diagram showing the extent of overlap between GC-specific genes (grey), MC-specific genes (blue) and genes downregulated in *wsb* (green). For RNA-seq analysis of *wsb-1* mutants, the elongating top 1 cm of the stem without flowers was collected at 25–30 DAG, and total RNA was extracted. **i**, RT–qPCR analysis of *MYR002* in the WT and *wsb-1* (*n* = 6). The elongating top 1 cm of the stem without flowers was collected at 25–30 DAG for RT–qPCR. Open circles represent individual data points. Data are means ± s.d. A two-tailed unpaired Student’s *t*-test was used to calculate the *P* values between WT and *wsb*. *P < 0.05. **j**, Immunoblot analysis of mVenus in *iWSB-mVenus* seedlings with anti-GFP antibodies. Seedlings at 4 DAG were transferred to inductive medium containing 10 µM oestrogen (+) or maintained on medium with no oestrogen (−) and incubated for 6 or 24 h. * indicates the molecular weight of WSB-mVenus fusion proteins. Marker, a molecular weight marker; em, an empty lane. **k**, Time-course analysis of relative *MYR002* expression levels in *iWSB-mVenus* seedlings at 4 DAG with or without oestrogen (10 µM) treatment. *n* = 6 (6, 10 and 24 h) and 7 (2, 4 and 8 h). Data are means ± s.d. A two-tailed unpaired Student’s *t*-test was used to calculate the *P* values between expression with and without oestrogen treatment. *P < 0.05. Dashed box is enlarged in inset. **l**, Cells ectopically expressing *MYR002* are induced by the transient overexpression of *WSB-mVenus*. Top: phenotypes of cotyledons from *iWSB-mVenus* seedlings at 4 DAG with or without 48 h of oestrogen treatment (10 µM). *MYR002*-expressing cells were visualized by GUS staining (blue). Bottom: enlarged images of cotyledon. Arrowheads, GCs. **m**–**o**, Time-course analysis of relative expression levels for At1g23170 (**m**), *TET4* (**n**) and *HIPP20* (**o**) in *iWSB-mVenus* seedlings at 4 DAG with or without oestrogen (10 µM) treatment. *n* = 6 (6, 10 and 24 h) and 7 (2, 4 and 8 h). The relative expression levels were presented by setting the average expression level in mock-treated plants to 1. Data are means ± s.d. A two-tailed unpaired Student’s *t*-test was used to calculate the *P* values between expression with and without oestrogen treatment. *P < 0.05. Scale bars, 1 cm (**c**), 500 µm (**f** cotyledon top and **l** top), 200 µm (**f** sepal), 100 µm (**f** cotyledon bottom and **l** bottom MC) and 20 µm (**l** bottom stomata).

**Extended Data Fig. 4:**
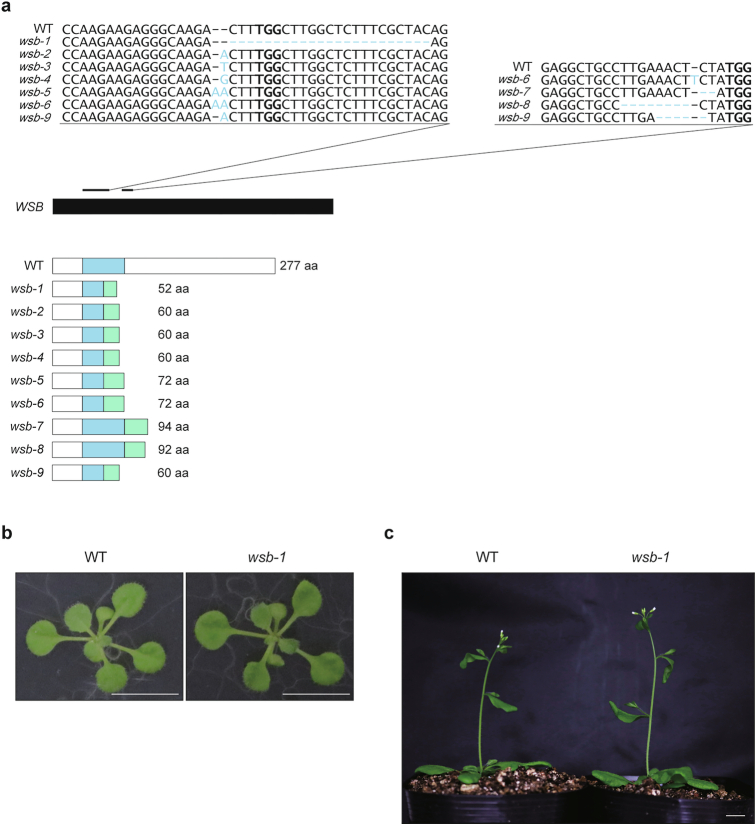
Generation of mutants of *WSB* by CRISPR/Cas9. **a**, Gene disruption of At3g57600 (*WSB*) in Arabidopsis using CRISPR/Cas9-mediated gene editing. Diagram of part of the *WSB* gene with the sgRNA target sites and resulting mutations in the newly obtained CRISPR/Cas9-generated alleles. The protospacer adjacent motif (PAM) for Cas9 is in bold; deletions or inserted nucleotides in the new mutant alleles are in blue. Black box, exon; blue box, AP2 domain; green box, amino acids after frameshifts that are different from the WT. aa, amino acids. **b**, The development of *wsb-1* mutant seedlings is comparable to that of WT seedlings at 14 DAG. **c**, The development of *wsb-1* mutant plants is comparable to that of WT plants at 28 DAG. Scale bars, 1 cm (**b** and **c**).

To clarify the biological functions of WSB in vivo, we generated loss-of-function mutants for *WSB* via clustered regularly interspaced short palindromic repeats (CRISPR)/CRISPR-associated protein 9 (Cas9)-mediated genome editing^48^, as no T-DNA insertion mutants with insertions in the exon are available. We obtained nine mutants (*wsb-1* to *wsb-9*) with frameshifts introducing premature stop codons (Fig. 3a,b and Extended Data Fig. 4a). All *wsb* mutants except *wsb-7* and *wsb-8* lacked a part of the sequence encoding the DNA-binding domain (the AP2 domain) (Fig. 3b and Extended Data Fig. 4a). Plant sizes of *wsb* mutants were comparable to those of the WT (Fig. 3c and Extended Data Fig. 4b,c).

### WSB is required for the differentiation of MCs

**Extended Data Fig. 5:**
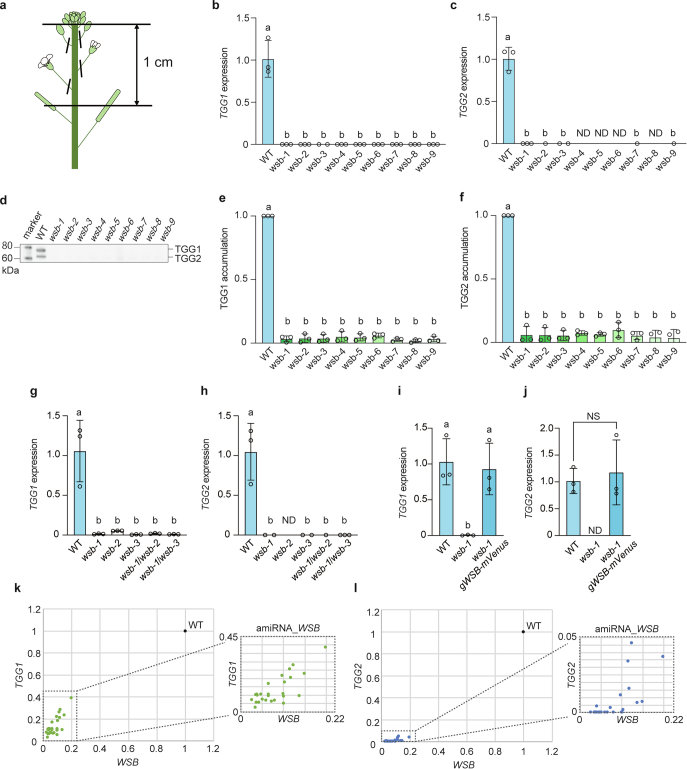
WSB is required for the expression of the myrosinase genes *TGG1* and *TGG2*. **a**, Diagram for the sampling from the top 1 cm of the stem without siliques, flowers, and flower primordia. Horizontal lines indicate where tissues were excised. **b**, **c**, RT-qPCR analysis of *TGG1* (**b**) and *TGG2* (**c**) transcript levels in the stems of the WT and *wsb* mutants. The elongating top 1 cm of the stem without flowers was collected at 25–30 DAG. Experiments were performed three times, and open circles represent individual data points. Data are means ± SD. Different lowercase letters indicate significant differences, as determined by one-way ANOVA test followed by Tukey-Kramer test (*p* < 0.05); ND, not detected. **d**, Immunoblot analysis of true leaves from the WT and *wsb* mutants at 14 DAG with anti-TGG1 and anti-TGG2 antibodies. Note that the immunoblot image in Fig. 3E was taken from this image. An image of the entire blot is shown in Supplementary Fig. 1. marker: a molecular weight marker. **e**, **f**, Quantitative analysis of the immunoblot data including the data shown in (**d**). Experiments were performed three times (see Supplementary Fig. 1 and Supplementary Table 14); open circles represent individual data points from each replicate. Data are means ± SD. Different lowercase letters indicate significant differences, as determined by one-way ANOVA test followed by Tukey-Kramer test (*p* < 0.05). **g**, **h**, Allelism tests between *wsb-1*, *wsb-2*, and *wsb-3*. RT-qPCR analysis of *TGG1* (**g**) and *TGG2* (**h**) transcript levels in true leaves of seedlings at 14 DAG for WT, *wsb-1*, *wsb-2*, *wsb-3*, *wsb-1*/*wsb-2* F_1_, and *wsb-1*/*wsb-3* F_1_. The *wsb-1*/*wsb-2* F_1_ and *wsb-1*/*wsb-3* F_1_ seedlings were generated by crossing. Experiments were performed three times; open circles represent individual data points from each replicate. Data are means ± SD. Different lowercase letters indicate significant differences, as determined by one-way ANOVA test followed by Tukey-Kramer test (*p* < 0.05). **i**, **j**, The *gWSB-mVenus* transgene rescues the phenotypes of *wsb-1*. RT-qPCR analysis of *TGG1* (**i**) and *TGG2* (**j**) transcript levels in the true leaves at 14 DAG for WT, *wsb-1*, and *wsb-1 gWSB-mVenus*. *wsb-1 gWSB-mVenus* was generated by transforming the *gWSB-mVenus* construct into *wsb-1*. Data are means ± SD. Experiments were performed three times; open circles represent individual data points from each replicate. Different lowercase letters indicate significant differences (**i**), as determined by one-way ANOVA test followed by Tukey-Kramer test (*p* < 0.05). A two-tailed Student’s *t*-test was used to calculate the *p*-values between WT and *wsb-1 gWSB-mVenus* (NS; not significant). **k, l**, Concentration-dependent effects of *WSB* on the expression levels of *TGG1* (**k**) and *TGG2* (**l**). RT-qPCR analysis of *TGG1* and *TGG2* transcript levels in WT and *WSB*-knockdown lines (amiRNA*-WSB*). Twenty-six independent T_1_ plants were analyzed. The black and green circles represent the expression levels in the true leaves at 14 DAG for *TGG1* from the WT and amiRNA*-WSB*, respectively. The black and blue circles represent the expression levels in the true leaves at 14 DAG for *TGG2* from the WT and amiRNA*-WSB*, respectively. The boxed area in the left plots is enlarged in the right plots.

**Extended Data Fig. 6:**
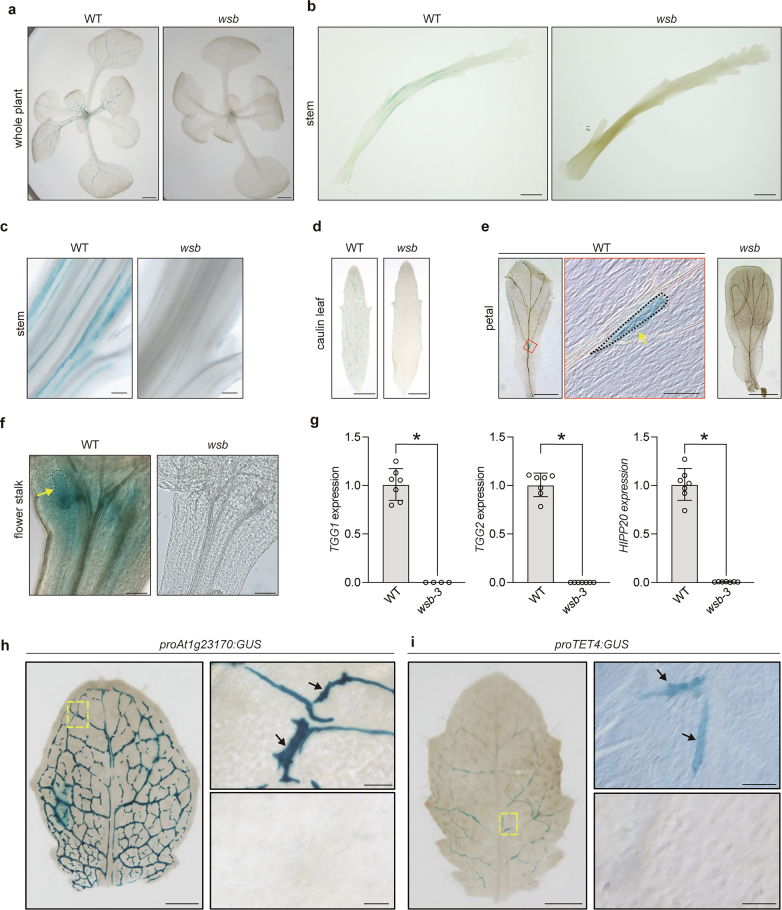
WSB is required for the development of MCs. **a–f**, GUS staining of entire plants (**a**), stems (**b** and **c**), cauline leaves (**d**) petals (**e**), and flower stalks (**f**) for WT and *wsb-3* plants harboring the MC reporter *MYR001:GUS*. For (**a**), seedlings at 16 DAG were used. For (b-f), plants at 25–30 DAG were used. The boxed area in (**e**) is enlarged in the right panel. Arrows, MCs. **g**, RT-qPCR analysis of *TGG1*, *TGG2*, and *HIPP20* transcript levels in the stems of WT and *wsb-1* mutants. The elongating top 1 cm of the stem without flowers was collected at 25–30 DAG. Experiments were performed seven times, and open circles represent individual data points. Data are means ± SD. A two-tailed unpaired Student’s *t*-test was used to calculate the *p*-values between *wsb* and Col-0. *, *p* < 0.05. **h**, **i**, GUS staining of a rosette leaf from WT Arabidopsis (Col-0) harboring *proAt1g2317020:GUS* (**h**) and *proTET4:GUS* (**i**) at 14 DAG. Enlarged image of myrosin lineage cells (upper right) and epidermal stomatal lineage cells (lower right). Arrows indicate MCs along veins. Scale bars, 1 mm (**a, b, d**), 500 µm (**e**, **h**, **i**), 100 µm (**c, f**), 50 µm (**e**, enlarged, **h**, **i**, upper right), and 20 µm (**h**, **i**, lower right).

To examine whether WSB, similar to FAMA, is required for the development of MCs, we characterized MC development in *wsb* mutants by RT–qPCR analysis for the endogenous MC markers *TGG1* and *TGG2*. We detected no transcripts for these genes (Fig. 3d and Extended Data Fig. 5a–c) and much lower TGG1 and TGG2 abundance in *wsb* mutants by immunoblot analysis (Fig. 3e, Extended Data Fig. 5d–f and Supplementary Figs. 1–4). *TGG1* and *TGG2* transcripts were largely undetectable in the F_1_ progenies of *wsb-1*/*wsb-2* and *wsb-1*/*wsb-3* (Extended Data Fig. 5g,h); however, the fluorescent translational fusion construct *gWSB-mVenus* fully rescued *TGG1* and *TGG2* expression levels when introduced into the *wsb-1* mutant, indicating that the WSB-mVenus fusion is fully functional (Extended Data Fig. 5i,j). In addition, *WSB* showed a dosage-dependent effect on the expression of myrosinase genes, based on the analysis of *WSB*-knockdown lines expressing an artificial microRNA designed to decrease *WSB* transcript levels (Extended Data Fig. 5k,l). Collectively, WSB is essential for the expression of myrosinase genes. We also examined MC development in *wsb-3* mutants with the MC-specific reporter *MYR001:GUS*^24,31^. We detected barely any activity for the reporter along the vasculature of whole *wsb-3* seedlings, leaves (cotyledons, true leaves and cauline leaves), stems and flowers (sepals, petals and flower stalks), whereas MCs developed normally in the WT, suggesting that MCs fail to differentiate in *wsb* (Fig. 3f,g and Extended Data Fig. 6a–f). The defects in MC development seen in *wsb* were reminiscent of those in *fama*^25,26^, suggesting that *WSB* is a critical downstream target of FAMA for the development of MCs.

### WSB governs transcriptional networks for both MCs and GCs

To explore the transcriptional networks regulated by WSB, we performed a comparative RNA-seq analysis of WT and *wsb-1* seedlings (Fig. 3h green and Supplementary Data 7). We identified 383 downregulated genes in *wsb*, including *TGG1* and *HIPP20* (MC and GC markers) as well as *TGG2* (MC marker), suggesting that WSB is required for their expression (Fig. 3h, Extended Data Fig. 6g and Supplementary Data 7). When compared to single-cell RNA-seq (scRNA-seq) data for MCs and GCs from mature leaves^49^, 54 and 42 of the 383 downregulated genes were expressed specifically in MCs and GCs, respectively, with another 114 expressed in both MCs and GCs (Fig. 3h and Supplementary Data 8). These results suggest that WSB governs transcriptional networks for the differentiation of both MCs and GCs, in agreement with the *WSB* expression pattern.

To verify whether WSB can activate MC-specific genes in various cell types, among the 54 genes above, we focused on the TF gene *WRKY23* (hereafter referred to as *MYR002*), as we had previously identified it as a FAMA downstream gene^26^ and it was reported to be an MC-specific marker gene on the basis of scRNA-seq data^50^. In addition, *WRKY23* is one of the genes co-expressed with *WSB* in the ATTED-II database^42^ (Fig. 1g). We confirmed that *MYR002* expression levels are much lower in *wsb-1* (Fig. 3i), suggesting that WSB is required for *MYR002* expression. To test the sufficiency of WSB for *MYR002* expression, we generated an oestrogen-inducible *WSB-mVenus* line (hereafter referred to as *iWSB-mVenus*). We detected WSB-mVenus in *iWSB-mVenus* after 6 h of oestrogen treatment, with higher levels reached after 24 h of oestrogen treatment (Fig. 3j and Supplementary Figs. 5–7). The expression levels of *MYR002* rapidly rose >3-fold after 2 h of continuous oestrogen treatment, reaching >60-fold higher levels at 24 h under oestrogen treatment compared with mock-treated *iWSB-mVenus* seedlings (Fig. 3k). We also examined GUS staining in *iWSB-mVenus MYR002:GUS* seedlings generated by crossing the two independent transgenic lines. In mock-treated seedlings, consistent with a previous report^50^, we specifically observed GUS staining in MCs but not in GCs (Fig. 3l). In seedlings treated with oestrogen for 48 h, we detected GUS signals throughout the cotyledons, including pavement cells and mesophyll cells, as well as a strong signal in the vasculature (Fig. 3l top middle and right). Notably, we also observed GUS signals in GCs (Fig. 3l lower right). In addition, WSB induced the expression of other MC marker genes (*HIPP20*, At1g23170 and *TETRASPANIN 4* (*TET4*, At5g60220)) (Fig. 3m–o and Extended Data Fig. 6h,i). Notably, WSB did not induce the expression of *MYR001* (Supplementary Fig. 8). Taken together, these results suggest that WSB can activate the expression of a subset of MC marker genes in various cell types.

### *SCAP1* is a stomatal lineage-specific direct target of FAMA

**Fig. 4:**
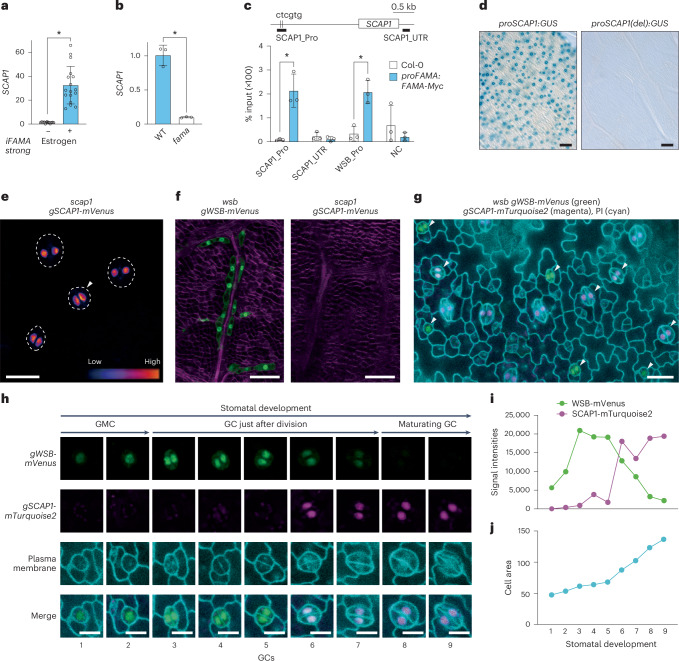
*SCAP1* is a stomatal lineage-specific direct target gene of FAMA. **a**, RT–qPCR analysis of *SCAP1* transcript levels in *iFAMA*^*strong*^ seedlings at 4 DAG with (*n* = 17) or without (*n* = 13) oestrogen (10 µM) treatment for 24 h. Open circles represent individual data points. Data are means ± s.d. A two-tailed unpaired Student’s *t*-test was used to calculate the *P* values between expression with and without oestrogen treatment. *P < 0.05. **b**, RT–qPCR analysis of *SCAP1* transcript levels in whole seedlings at 14 DAG of the WT and *fama* (*n* = 3). Open circles represent individual data points. Data are means ± s.d. A two-tailed unpaired Student’s *t*-test was used to calculate the *P* values between the WT and *fama*. *P < 0.05. **c**, ChIP–qPCR binding assay of FAMA at the *SCAP1* locus (*n* = 3; % input). The percentage of input of FAMA-Myc at *SCAP1* was analysed using *proFAMA:FAMA-Myc* seedlings at 4 DAG. Whole seedlings were used as samples for the extraction of DNA–protein complexes. Open circles represent the percentage of input from each sample. Data are means ± s.d. A two-tailed unpaired Student’s *t*-test was used to calculate the *P* values between *proFAMA:FAMA-Myc* and Col-0. *P < 0.05; NC, negative control. **d**, GUS staining of the epidermis from true leaves of WT (Col-0) at 14 DAG, harbouring the reporter construct *proSCAP1:GUS*^20^ or *proSCAP1(del):GUS*. For *proSCAP1:GUS*, the promoter fragment was from −1,770 to −1 bp; for *proSCAP1(del):GUS*, the promoter fragment was from −1,703 to −1 bp. For *proSCAP1(del):GUS*, 15 T_2_ lines were analysed. No GUS staining was observed in *proSCAP1(del):GUS*. **e**, Confocal image of the first or second leaves from a *scap1 gSCAP1-mVenus* seedling at 5 DAG. Signal intensities are shown in blue to red according to increasing intensity levels. Arrowhead, GC. **f**, Confocal image of inner tissues from fifth leaves of a *wsb gWSB-mVenus* (left) or *scap1 gSCAP1-mVenus* true leaves (right) at 8 DAG. mVenus signal is shown in green and the signal from the dye SR2200 (cell wall) is shown in purple. Note that an mVenus signal was hardly detected in *scap1 gSCAP1-mVenus*. **g**, Confocal images of the sixth true leaf from transgenic seedlings at 10 DAG, harbouring *gWSB-mVenus* and *gSCAP1-mTurquoise2* (shown in green and magenta, respectively) in the *wsb-1* mutant background. Cell walls were stained with propidium iodide (cyan). Stomatal-lineage cells indicated by arrowheads are enlarged in **h**. **h**, Confocal images of each and merged channels for nine GCs indicated by arrowheads in **g**. GCs were arranged along with stomatal development, which was estimated by GC size. Note that WSB-mVenus and SCAP1-mTurquoise2 co-accumulated at stages 6 and 7. **i**, Quantification of fluorescence signals for WSB-mVenus (green) and SCAP1-mTurquoise2 (magenta) through stomatal development using LAS X quantification mode. **j**, Quantification of cell area for GCs through stomatal development using LAS X quantification mode. Scale bars, 80 µm (**d**), 40 µm (**f**), 20 µm (**e**,**g**), 10 µm (**h**).

**Extended Data Fig. 7:**
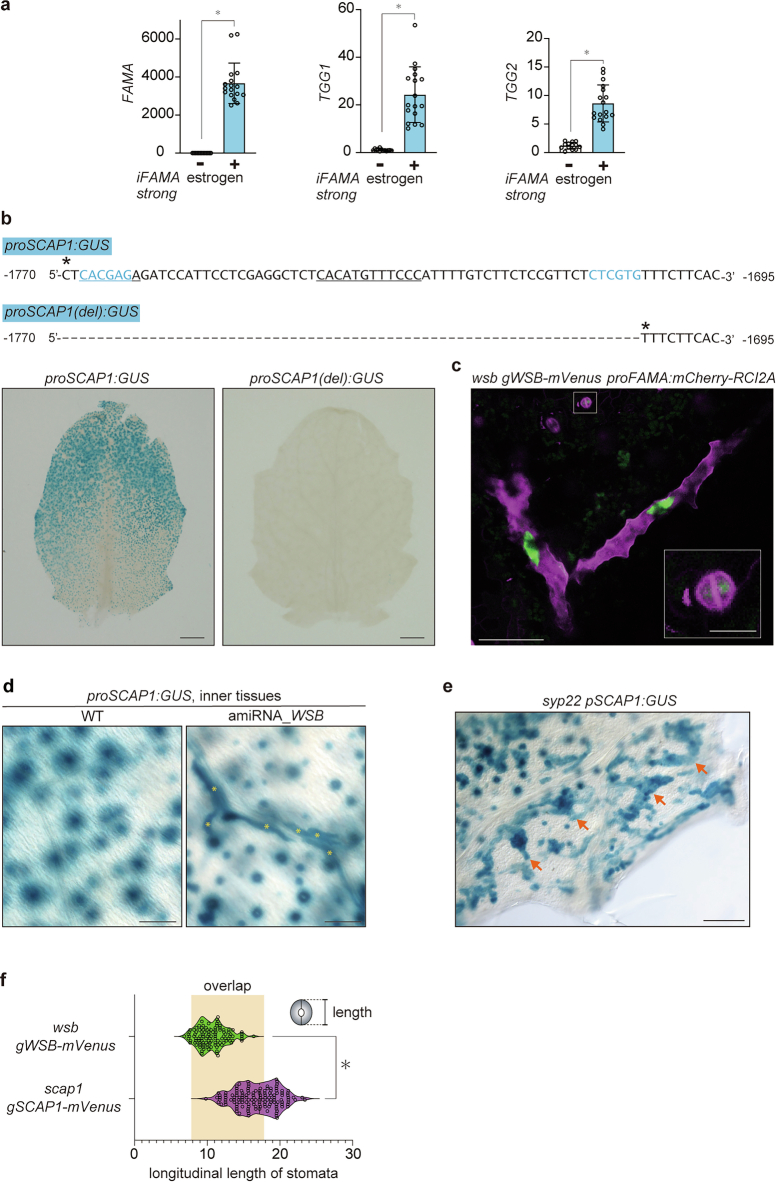
Expression pattern of *SCAP1.* **a**, Establishment of strong *iFAMA* lines. Relative transcript levels of *FAMA*, *TGG1*, and *TGG2* in seedlings at 4 DAG from strong *iFAMA* lines with (+, *n* = 17) or without (−, *n* = 13) a 24-h treatment with oestrogen (10 µM). Open circles represent individual data points. Data are means ± SD. Note that the expression levels of the three tested genes were significantly upregulated by oestrogen treatment compared to samples without oestrogen treatment. A two-tailed Student’s *t*-test was used to calculate the *p*-values (*, *p* < 0.05). **b**, Partial sequences of the *SCAP1* promoter ( − 1,770 to −1,695 bp) constructs. Blue indicates two CTCGTG motifs ( − 1,768 to −1,763 bp, antisense; −1,709 to −1,704 bp, sense). The underlined sequences indicate two *cis*-elements that are required for the expression of *SCAP1*^23^. Asterisks, start positions of the promoter fragments for *GUS* reporter lines. A 67-bp region of the *SCAP1* promoter is required for *SCAP1* expression. *proSCAP1:GUS*, DNA fragment from −1,770 to −1 bp; *proSCAP1(del):GUS*, DNA fragment from −1,703 to −1 bp. GUS staining of true leaves from WT Arabidopsis (Col-0) carrying *proSCAP1:GUS*^22^ or *proSCAP1(del):GUS* at 16 DAG is shown. For *proSCAP1(del):GUS*, 15 T_2_ lines were analyzed. Note that no GUS staining was observed in *proSCAP1(del):GUS*. **c**, Confocal images of true leaves from *wsb* harboring g*WSB-mVenus* (green) and *proFAMA:mCherry-RCI2A* (magenta) at 14 DAG. mCherry-RCI2A, plasma membrane marker. WSB highly accumulates in the nucleus of mature MCs (nuclear size is larger than 30 µm). **d**, GUS staining of inner tissues for true leaves from WT Arabidopsis (Col-0) and *WSB*-knockdown lines (amiRNA*-WSB*) harboring *proSCAP1:GUS* at 14 DAG. Note that ectopic expression of *SCAP1* was detected in MCs of amiRNA*-WSB* (yellow asterisks). Round, fuzzy GUS signals are derived from out-of-focus GUS signals from GCs. **e**, GUS staining of true leaves from *syp22* seedlings at 14 DAG harboring *proSCAP1:GUS*. Orange arrows, clusters of MCs. **f**, Longitudinal length (µm) of stomata expressing *WSB-mVenus* (green, *n* = 108, including two GMCs) or *SCAP1-mVenus* (purple, *n* = 135). Note that the expression windows of *WSB-mVenus* and *SCAP1-mVenus* partially overlap. Two-tailed unpaired Student’s *t*-test was used to calculate the *p*-values between *WSB-mVenus* and *SCAP1-mVenus*. *, *p* < 0.05. Scale bars, 500 µm (**b**); 100 µm (**e**);50 µm (**c** and **d**); 10 µm (**c**, inset).

*WSB* was expressed in stomatal-lineage cells (Fig. 2), and RNA-seq analysis revealed that loss of WSB function decreased the expression of 156 GC-related genes (Fig. 3h), suggesting that WSB may be involved in stomatal development. However, in our cultivation conditions, we did not see clear changes in stomatal morphology in *wsb* single mutants. We hypothesized that *WSB* and other direct FAMA target genes might function synergistically in stomatal development. We focused on the DOF-type TF gene *SCAP1* on the basis of the following reasoning, although we did not identify *SCAP1* as a differentially expressed gene in the RNA-seq analysis of *iFAMA* (Fig.1b). First, *SCAP1* was downregulated in *scrm* (Extended Data Fig. 1c). Second, FAMA was reported to activate *SCAP1* expression in different *FAMA*-inducible lines^20^, and 50% of all stomata in *scap1* mutants show skewed morphologies^22^. Third, *SCAP1* is the only other co-expressed TF-encoding gene with *FAMA* with strong correlation (correlation value 1–5; bold lines in Fig. 1g) in the ATTED-II database^42^ together with *WSB*. As the inducibility of the oestrogen-inducible system varied among different transgenic lines, we generated another *FAMA* overexpression line (hereafter referred to as *iFAMA*^*strong*^), in which we induced *FAMA* more strongly than in the original *iFAMA* line (Extended Data Fig. 7a, compare with Extended Data Fig. 1b). In *iFAMA*^*strong*^, we observed *FAMA* overexpression phenotypes, as reported previously^20^ (Supplementary Fig. 9). Using *iFAMA*^*strong*^ and consistent with the previous report^20^, we determined that *SCAP1* expression is indeed upregulated by FAMA (Fig. 4a). Conversely, *SCAP1* expression levels were lower in the *fama* mutant (Fig. 4b). In addition, we established that FAMA directly binds to the *SCAP1* promoter regions by ChIP–qPCR (Fig. 4c) using a primer pair that amplifies a *SCAP1* promoter fragment containing two CTCGTG motifs (Fig. 4c and Extended Data Fig. 7b). A *proSCAP1:GUS* reporter line showed specific GUS staining in GCs (Fig. 4d and Extended Data Fig. 7b)^22^. By contrast, the *proSCAP1*_delta_67_bp*:GUS* reporter construct lacking the two CTCGTG motifs produced no GUS signal in GCs (Fig. 4d and Extended Data Fig. 7b). Consistent with these results, we noticed two *cis* elements, recently reported to be critical for *SCAP1* expression^23^, within the 67-bp DNA fragment deleted in *proSCAP1*_delta_67_bp*:GUS* (Extended Data Fig. 7b). We barely detected any *SCAP1* expression in MCs, as evidenced by the very low GUS staining in *proSCAP1:GUS* seedlings and Venus fluorescence in *gSCAP1-mVenus* seedlings (Fig. 4d,f Extended Data Fig. 7b). We rarely found a signal for SCAP1-mVenus in the nucleus of large MCs (Supplementary Fig. 10). These results confirm that *SCAP1* is a stomatal lineage-specific gene that is also a direct target of FAMA.

We hypothesized that WSB might inhibit *SCAP1* expression in MCs because the expression of *WSB* persisted longer in MC lineages than in GCs (Fig. 2e–g and Extended Data Fig. 7c). Consistent with this idea, we frequently detected the ectopic expression of *SCAP1* in MCs of *WSB*-knockdown lines (Extended Data Fig. 7d). Importantly, we did not notice any clear difference in the expression window of *SCAP1* in stomatal lineages between the WT and *WSB*-knockdown lines (Supplementary Fig. 11). These results suggest that the sustained expression and/or high levels of *WSB* repressed the expression of *SCAP1* specifically in MC lineages. In addition, we detected ectopic expression of *SCAP1* in a subset of MCs in *syp22* leaves, in which polar auxin transport (PAT) is perturbed^39^, as indicated by a previous transcriptome analysis (Extended Data Fig. 7e)^26^. These results suggest that *WSB* and PAT repress the expression of the guard cell-specific gene *SCAP1* in the MC lineage, resulting in the fate specification of MCs.

### *WSB* and *SCAP1* are co-expressed in GCs just after division

We examined the accumulation of SCAP1 protein with a translational fusion construct introduced into the *scap1* mutant (*scap1 gSCAP1-mVenus*). In at least 10 independent transgenic lines, we detected expression of the *gSCAP1-mVenus* reporter as patches in the epidermis, highlighting the accumulation of SCAP1 in the nuclei of GCs, but not of GMCs (Fig. 4e). In addition, we rarely detected SCAP1-mVenus in MCs (Fig. 4f). To clarify the expression window of *WSB* and *SCAP1* in the stomatal lineage, we compared the sizes of GMCs and GCs accumulating each protein (Extended Data Fig. 7f). Stomatal-lineage cells expressing *WSB-mVenus* were on average smaller than those expressing *SCAP1-mVenus*, while a subset of GCs accumulated both proteins, suggesting that *WSB* has an earlier expression window than *SCAP1*, although they partially overlap. To explore this hypothesis in more detail, we generated two translational reporter lines, *gWSB:mVenus* and *gSCAP1:mTurquoise2*; we transformed *wsb-1 gWSB-mVenus* plants using *Agrobacterium* harbouring *gSCAP1-mTurquoise2* constructs. Using this line, we visualized both WSB and SCAP1 fused to different fluorescent proteins in the same stomatal-lineage cells from GMCs to maturing GC stages (Fig. 4g,h and Supplementary Figs. 12–15) and quantified the signal intensities for WSB and SCAP1 (Fig. 4i and Supplementary Fig. 14), and cell area (Fig. 4j and Supplementary Fig. 14). From this analysis, we conclude that WSB and SCAP1 co-occur in the same GCs just after division (stages 6 and 7 in Fig. 4h–j and Supplementary Fig. 14) when guard cell area dramatically increases, suggesting that these two TFs contribute to the differentiation of GCs at the stage just after final division.

### WSB and SCAP1 act synergistically to differentiate GCs

**Fig. 5:**
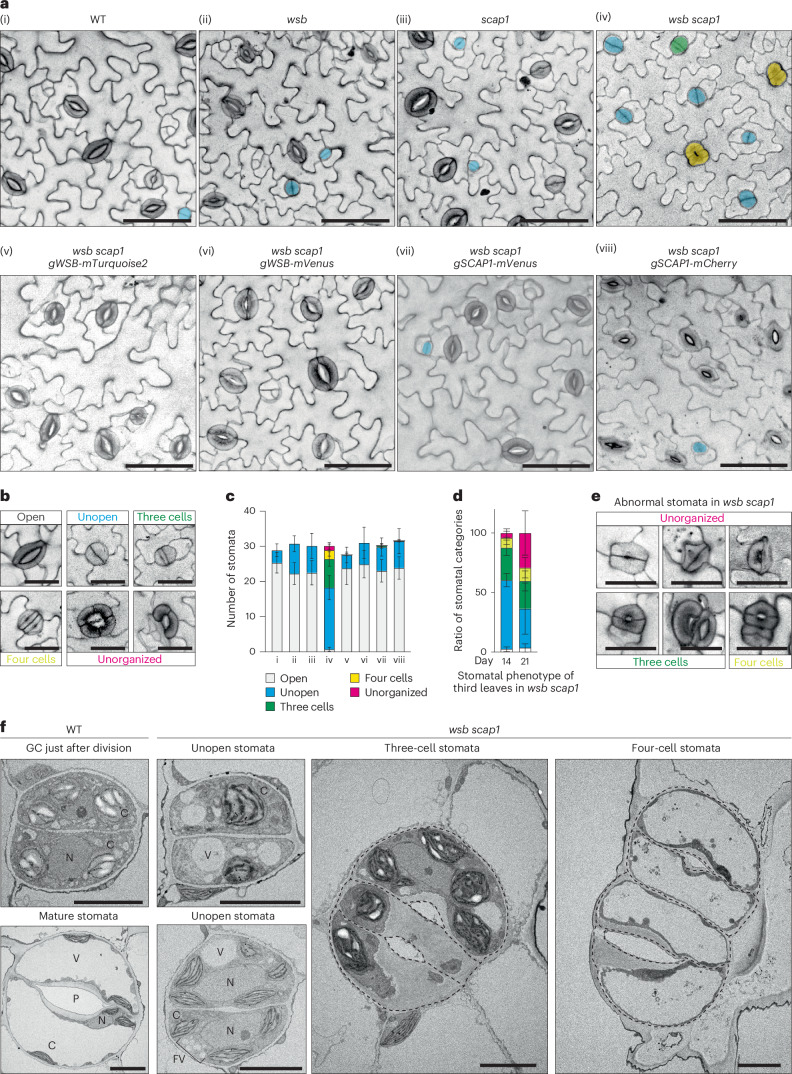
WSB and SCAP1 are synergistically required for the development of GCs. **a**, Representative confocal images (converted to greyscale) showing the abaxial epidermis from third true leaves of seedlings at 14 DAG from the indicated genotypes. Cell walls were stained with propidium iodide. GCs were manually traced and are highlighted in different colours (blue, unopened stomata; green, three-cell stomata; yellow, four-cell stomata). To generate *wsb scap1*, we used the *wsb-1* allele. **b**, Representative confocal images for each category of stomata. Cell walls were stained with propidium iodide. **c**, Number of stomata in the 0.308-mm^2^ field from the third true leaves of the indicated genotypes at 14 DAG in **a** (WT *n* = 9, *wsb* *n* = 8, *scap1* *n* = 8, *wsb scap1* *n* = 15, *wsb scap1 gWSB-mTurquoise2* *n* = 24, *wsb scap1 gWSB-mVenus* *n* = 9, *wsb scap1 gSCAP1-mVenus* *n* = 21, *wsb scap1 gSCAP1-mCherry* *n* = 13). Data are means ± s.d. **d**, Ratio of stomatal categories in the 0.308-mm^2^ field from the third true leaves of *wsb scap1* at 14 (*n* = 15) and 21 DAG (*n* = 11). Colour scheme as in **c**. Representative images of epidermal phenotypes from *wsb scap1* seedlings at 21 DAG are shown in Supplementary Fig. 16. Data are means ± s.d. **e**, Representative confocal images for the ‘unorganized’ category of stomata in *wsb scap1* at 21 DAG. Cell walls were stained with propidium iodide. **f**, Representative electron micrographs of stomata from the WT (left) and abnormal stomata in *wsb scap1* (right). True leaves from plants at 21 DAG were used, except for WT GCs just after division (14 DAG). P, pore; N, nucleus; C, chloroplast; V, vacuole; FV, fragmented vacuole. Scale bars, 50 µm (**a**), 25 µm (**b**,**e**) and 5 µm (**f**).

**Extended Data Fig. 8:**
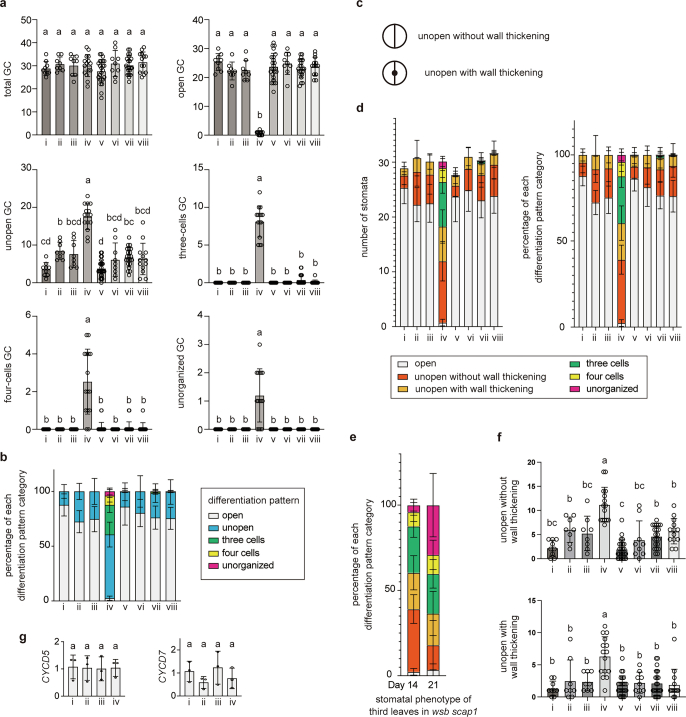
Synergistic interaction between *WSB* and *SCAP1* in the development of GCs. **a**, Number of GCs of in the 0.308-mm^2^ field of the third true leaves from the indicated genotypes at 14 DAG in each category (WT [i], *n* = 9; *wsb* [ii], *n* = 8; *scap1* [iii], *n* = 8; *wsb scap1* [iv], *n* = 15; *wsb scap1 gWSB-mTurquoise2* [v], *n* = 24; *wsb scap1 gWSB-mVenus* [vi], *n* = 9; *wsb scap1 gSCAP1-mVenus* [vii], *n* = 21; *wsb scap1 gSCAP1-mCherry* [viii], *n* = 13). Data are means ± SD. Different lowercase letters indicate significant differences, as determined by one-way ANOVA followed by Tukey-Kramer test. To generate *wsb scap1*, we used the *wsb-1* allele. **b**, Percentage of each differentiation pattern category in seedlings at 14 DAG for the indicated genotypes (WT [i], *n* = 9; *wsb* [ii], *n* = 8; *scap1* [iii], *n* = 8; *wsb scap1* [iv], *n* = 15; *wsb scap1 gWSB-mTurquoise2* [v], *n* = 24; *wsb scap1 gWSB-mVenus* [vi], *n* = 9; *wsb scap1 gSCAP1-mVenus* [vii], *n* = 21; *wsb scap1 gSCAP1-mCherry* [viii], *n* = 13). Grey, open stomata; blue, unopened stomata; green, three-cell stomata; yellow, four-cell stomata; pink, unorganized stomata. Data are means ± SD. **c**, Diagram of two different types of unopen stomata. **d**, Left, number of stomata in the 0.308-mm^2^ field of the third true leaves for the indicated genotypes at 14 DAG in **a** (WT, *n* = 9; *wsb*, *n* = 8; *scap1*, *n* = 8; *wsb scap1*, *n* = 15; *wsb scap1 gWSB-mTurquoise2*, *n* = 24; *wsb scap1 gWSB-mVenus*, *n* = 9; *wsb scap1 gSCAP1-mVenus*, *n* = 21; *wsb scap1 gSCAP1-mCherry*, *n* = 13). Grey, open stomata; orange, unopen without wall thickening; light orange, unopen with wall thickening; green, three-cell stomata; yellow, four-cell stomata; pink, unorganized stomata. Data are means ± SD. Right, ratio of stomatal categories for third true leaves from *wsb scap1* at 14 DAG. **e**, Ratio of stomatal categories in the 0.308-mm^2^ field for third true leaves of *wsb scap1* at 14 and 21 DAG. The color scheme is the same as in (d). **f**, Number of GCs of in the 0.308-mm^2^ field of the third true leaves for the indicated genotypes at 14 DAG in each category (WT [i], *n* = 9; *wsb* [ii], *n* = 8; *scap1* [iii], *n* = 8; *wsb scap1* [iv], *n* = 15; *wsb scap1 gWSB-mTurquoise2* [v], *n* = 24; *wsb scap1 gWSB-mVenus* [vi], *n* = 9; *wsb scap1 gSCAP1-mVenus* [vii], *n* = 21; *wsb scap1 gSCAP1-mCherry* [viii], *n* = 13). Data are means ± SD. Different lowercase letters indicate significant differences, as determined by one-way ANOVA followed by Tukey-Kramer test. **g**, RT-qPCR analysis of *CYCD5* and *CYCD7* transcript levels in true leaves of WT, *wsb*, *scap1*, and *wsb scap1* seedlings at 14 DAG (*n* = 3). Experiments were performed three times, and open circles represent individual data points. Data are means ± SD. Different lowercase letters indicate significant differences, as determined by one-way ANOVA test followed by Tukey-Kramer test (*p* < 0.05).

To assess whether WSB and SCAP1 act synergistically in the differentiation of GCs, we crossed previously isolated *scap1* single mutants^22^ with *wsb-1* to obtain the *wsb scap1* double mutant. First, we examined stomatal development on the abaxial epidermis of the third true leaves of seedlings at 14 DAG. Unlike the WT and the single mutants, the *wsb scap1* double mutant had very few normal GCs (Fig. 5a), with fewer than 3% compared with the WT (Fig. 5b,c and Extended Data Fig. 8a,b). Instead, *wsb scap1* developed irregular GCs of four types: (1) GCs without a pore (unopened, blue), (2) three GCs in one stomatal unit (three cells, green), (3) a pair of stomata with direct contact (four cells, yellow) and (4) unorganized GCs, for example, four cells in a single stoma and a discontinuous circle of stomata (unorganized, pink) (Fig. 5a–c and Extended Data Fig. 8a,b). Furthermore, a fraction of unopen GCs had strong propidium iodide (PI) staining at the centre of the division plane (Extended Data Fig. 8c–f). However, in many cases, we did not find clear pores with strong accumulation of PI, suggesting that strong PI signals indicate wall thickening rather than pore formation. We counted the number of unopen GCs with/without wall thickening (Extended Data Fig. 8c–f). Over 60% of stomata in *wsb scap1* were unopened GCs at this stage, with a diameter similar to that of WT GCs just after division, suggesting that unopened GCs in *wsb scap1* are generated by the proper division of GMCs, then stall at the GC stage just after division, without forming a pore. Consistent with this scenario, the expression levels of two cyclin D genes, *CYCD5* and *CYCD7*, which promote GMC division^51,52^, were not clearly different and almost comparable to those in *wsb scap1*, the WT and the single mutants (Extended Data Fig. 8g). To rule out the possibility that stomatal phenotypes might be due to potential off-target effects during the generation of each single mutant using genome editing and chemicals, we introduced complementation constructs into the *wsb scap1* double mutant: *proWSB:WSB-mTurquoise2*, *proWSB:WSB-mVenus*, *proSCAP1:SCAP1-mVenus*, *proSCAP1:SCAP1-mCherry* and *proSCAP1:SCAP1-mTurquoise2*. All complementation constructs restored stomatal phenotypes to WT levels (Fig. 5a–c, Extended Data Fig. 8 and Supplementary Fig. 12), validating the hypothesis that a functional WSB or SCAP1 is required to ensure normal stomatal development.

Next, to examine whether unopen GCs in *wsb scap1* differentiate into normal GCs or abnormal GCs at later time points, we investigated stomatal development on the abaxial epidermis of third true leaves from WT and *wsb scap1* seedlings at 14 and 21 DAG (Fig. 5d,e, Extended Data Fig. 8e and Supplementary Fig. 16). The frequency of unopen GCs (blue in Fig. 5d) was lower, while that of unorganized and irregular GCs (green, yellow and pink in Fig. 5d) was higher at 21 DAG than at 14 DAG. In addition, at 21 DAG, we observed more types of abnormal stomata in *wsb scap1*, some of which were not seen at 14 DAG (Fig. 5e). Some of the three-cell stomata and four-cell stomata seen in 21-DAG seedlings had pores (Fig. 5e,f). These results suggest that unopen GCs at 14 DAG might have the potential to differentiate, but they failed to properly differentiate into normal GCs, instead differentiating into irregular GCs at 21 DAG.

Finally, we compared GCs just after division and mature GCs between WT and *wsb scap1* at a structural level by transmission electron microscopy (TEM) (Fig. 5f). Almost all organelles in GCs just after division appeared similar in the two genotypes. However, we noticed the accumulation of starch granules (bright white colour) in the chloroplasts of GCs at the stage just after division in the WT, while starch rarely accumulated in the chloroplasts of *wsb scap1* GCs at the same stage, suggesting that starch biosynthesis is inhibited or that synthesized starch is quickly degraded in the chloroplasts of GCs following the loss of WSB and SCAP1 function. In addition, consistent with our confocal microscopy observations, we detected three-cell stomata and four-cell stomata in the TEM images (Fig. 5f). In addition, in some GCs of *wsb scap1*, the development of vacuoles was severely inhibited (Fig. 5f three-cell stomata). Overall, these results suggest that WSB and SCAP1 act synergistically in the differentiation of GCs.

Fewer GCs result in decreased CO_2_ uptake and photosynthesis. Consistent with this scenario, the *wsb scap1* double mutant displayed a dwarf phenotype (Supplementary Fig. 17). The degree of dwarfism was less severe than that of *fama* mutants (for example, *wsb scap1* produced seeds, unlike *fama*) (Supplementary Fig. 17). This difference might be explained by the presence of a few normal stomata in *wsb scap1* (Fig. 5a–c).

FAMA represses the expression of *CYCD7* to inhibit unnecessary GC divisions. Therefore, *fama* tumours, which are undifferentiated stomatal-lineage cells with symmetric divisions in multiple places along the longest wall, are induced by the overexpression of *CYCD7* (ref. ^52^). We thus asked whether the abnormal GCs in *wsb scap1* undergo additional divisions by introducing the *proFAMA:CYCD7-mVenus* reporter. We observed small cell clusters with random orientation for the division plane in the epidermis instead of unopen GCs, but these cell clusters were not identical to *fama* tumours in terms of the orientation of the cell divisions (Supplementary Fig. 18). These results suggest that CYCD7 can induce random divisions of stomatal-lineage cells in *wsb scap1* instead of the symmetric divisions or division along the longest wall previously reported in *fama*.

### Genome-wide mapping of WSB-binding sites

**Fig. 6:**
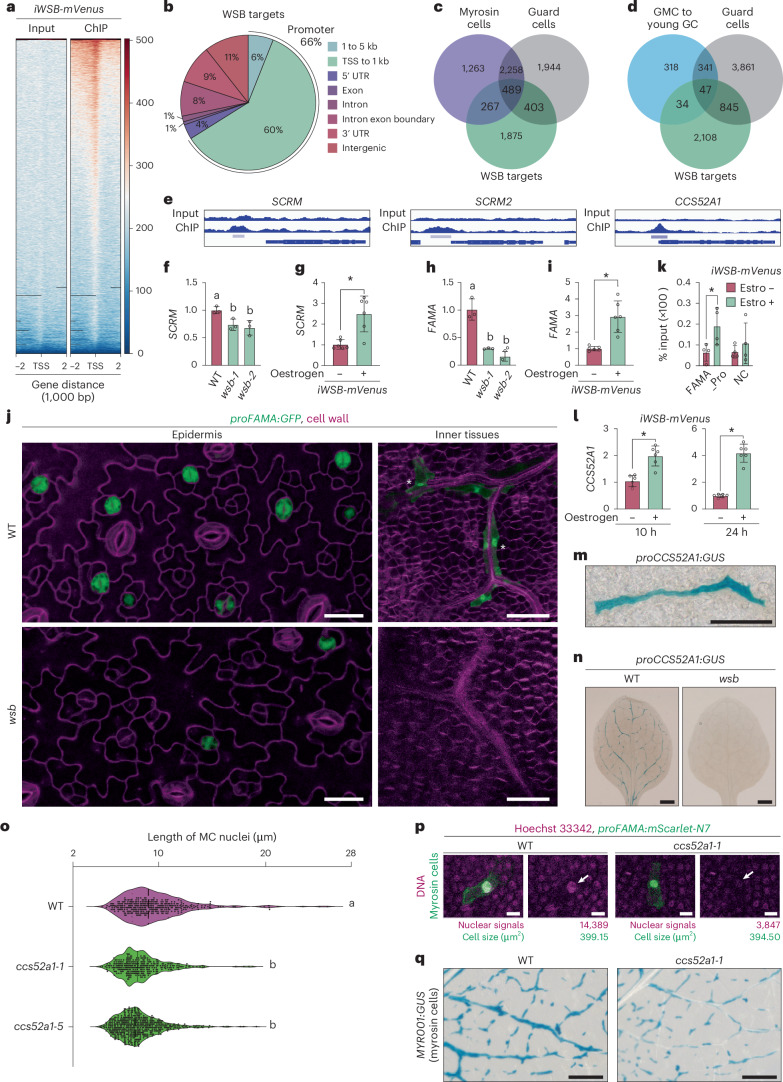
Genome-wide mapping of WSB-binding sites reveals positive feedback regulation between WSB and the FAMA–SCRM complex and specific roles for CCS52A1 in the differentiation of MCs. **a**, ChIP analysis of WSB-binding sites in *iWSB-mVenus* seedlings at 4 DAG with oestrogen treatment (0.01 µM, 24 h). Left: input sample. Right: ChIP sample. Panels show read density heat maps around each detected peak. **b**, Pie chart showing the percentage of WSB-bound peaks according to their functional genomic context. **c**, Venn diagram showing the extent of overlap between WSB-bound genes (green), MC-specific genes (purple) and GC-specific genes (grey). **d**, Venn diagram showing the extent of overlap between WSB-bound genes (green), GMC to young GC-specific genes (blue) and GC-specific genes (grey). GMC to young GC-specific genes comprised genes in the sf2–sf5 stages reported in the scRNA-seq data of stomatal-lineage cells (ref. ^53^). **e**, WSB ChIP-seq signals at the *SCRM*, *SCRM2* and *CCS52A1* loci. The gene models are shown as blue bars and lines at the bottom of each panel. Significant enrichment regions are marked by light purple bars. Peak calling was performed using MACS2 (*q*-value < 0.01). **f**, RT–qPCR analysis of *SCRM* transcript levels in the true leaves of the WT and *wsb* mutants at 14 DAG (*n* = 3). Experiments were performed 3 times; open circles represent individual data points from each replicate. Data are means ± s.d. Different lowercase letters indicate significant differences, as determined using one-way ANOVA followed by Tukey–Kramer test (*P* < 0.05). **g**, RT–qPCR analysis of *SCRM* transcript levels in *iWSB-mVenus* seedlings at 4 DAG with or without oestrogen treatment (10 µM) for 24 h (*n* = 6). Experiments were performed 6 times and open circles represent individual data points. Data are means ± s.d. A two-tailed Student’s *t*-test was used to calculate the *P* values between with and without oestrogen. *P < 0.05. **h**, RT–qPCR analysis of *FAMA* transcript levels in the true leaves of the WT and *wsb* mutants at 14 DAG (*n* = 3). Experiments were performed 3 times and open circles represent individual data points. Data are means ± s.d. Different lowercase letters indicate significant differences, as determined using one-way ANOVA followed by Tukey–Kramer test (*P* < 0.05). **i**, RT–qPCR analysis of *FAMA* transcript levels in *iWSB-mVenus* seedlings at 4 DAG with or without oestrogen (10 µM) treatment for 24 h (*n* = 6). Experiments were performed 6 times and open circles represent individual data points. Data are means ± s.d. A two-tailed Student’s *t*-test was used to calculate the *P* values between with and without oestrogen. *P < 0.05. **j**, Confocal images of epidermis (left) and inner tissues (right) from *proFAMA:GFP* (top) and *wsb proFAMA:GFP* (bottom). For the epidermis, the 11th true leaves at 22 DAG were examined; for inner tissues, the 12th leaves at 23 DAG were observed. GFP signal is shown in green and cell walls were stained with propidium iodide (left) or SR2200 (right). Cell walls are shown in magenta. Asterisks indicate MCs. **k**, ChIP–qPCR assay showing the binding of WSB to the *FAMA* locus (*n* = 3). The percentage of input of WSB-mVenus at *FAMA* was analysed using *iWSB-mVenus* seedlings at 4 DAG with or without treatment with 10 µM oestrogen for 24 h. Open circles represent individual data points. Data are means ± s.d. A two-tailed Student’s *t*-test was used to calculate the *P* values between with and without oestrogen for each genomic locus. *P < 0.05. NC, negative control. **l**, RT–qPCR analysis of *CCS52A1* transcript levels in *iWSB-mVenus* seedlings at 4 DAG with or without oestrogen (10 µM) treatment for 10 or 24 h (*n* = 6). Experiments were performed 6 times and open circles represent individual data points. Data are means ± s.d. A two-tailed Student’s *t*-test was used to calculate the *P* values between with and without oestrogen. *P < 0.05. **m**, GUS staining of a rosette leaf from WT Arabidopsis (Col-0) seedling at 14 DAG, harbouring the *proCCS52A1:GUS* reporter. **n**, GUS staining of the first or second true leaves from WT Arabidopsis (Col-0) and *wsb-1* seedlings at 8 DAG, harbouring the *proCCS52A1:GUS* reporter. **o**, Nucleus length (µm) from MCs in the third and fourth true leaves of WT and *ccs52a1* seedlings at 8 DAG (WT *n* = 352, *ccs52a1-1* *n* = 317, *ccs52a1-5* *n* = 555). MC nuclei were labelled with the *proFAMA:mScarlet-N7* marker. Samples were cleared using ClearSee. In this graph, the individual data points mask the lines showing the median, first quartile (25th percentile) and third quartile (75th percentile). See another version of this graph without individual data points in Supplementary Fig. 19. **p**, Confocal images of nuclei from MCs stained with Hoechst 33342 in the fifth true leaves of WT and *ccs52a1-1* seedlings at 8 DAG. Signals of Hoechst 33342 fluorescence are correlated with DNA amounts (ploidy). MC nuclei were labelled with the *proFAMA:mScarlet-N7* marker. Samples were cleared using ClearSee and then stained with Hoechst 33342. Quantification of nuclear Hoechst 33342 signals and cell sizes was done using LAS X quantification mode. **q**, GUS staining of the ninth true leaves from WT and *ccs52a1-1* seedlings at 15 DAG, harbouring the MC marker reporter *MYR001:GUS*. Note that MCs in *ccs52a1-1* are smaller than those in the WT. Images of whole leaves are shown in Extended Data Fig. 9c. Statistical significance was tested using ANOVA followed by Tukey–Kramer test (**f**,**h**,**o**, *P* < 0.05) or a Student’s *t*-test (**g**,**i**,**k**,**l**, *P* < 0.05). Scale bars, 500 µm (**n**), 200 µm (**q**), 100 µm (**m**), 40 µm (**j** right), 20 µm (**j** left) and 10 µm (**p**).

To identify the global binding events of WSB, we performed ChIP-seq for WSB using *iWSB-mVenus* and an anti-GFP antibody. We identified 3,199 WSB-binding sites corresponding to 3,034 loci (Fig. 6a and Supplementary Data 9). Consistent with the status of WSB as a TF, 66% of all WSB-binding peaks were associated with promoters, mostly within 1,000 bp upstream of the transcriptional start site (Fig. 6a,b and Supplementary Data 9). To categorize direct targets of WSB by expression pattern, we compared these data to scRNA-seq data for MCs and GCs from mature leaves^49^, revealing that 267, 403 and 489 of the 3,034 WSB target genes are expressed specifically in MCs, GCs or both, respectively (Fig. 6c). To categorize WSB targets more precisely along stomatal development, we compared the lists of WSB targets to genes expressed from the GMC stage to the mature GC stage^53^, identifying 81 WSB target genes expressed during this developmental gradient (Fig. 6d and Supplementary Data 10). These results indicate that WSB directly binds to the genomic regions of genes that are expressed in mature MCs and GCs, suggesting that WSB participates in the maturation of MCs and GCs.

We also identified *SCRM* and *SCRM2* among the genes bound by WSB; their protein products form heterodimers with FAMA (Fig. 6e). We focused on *SCRM* because we previously showed that myrosinase genes are markedly less expressed in *scrm*, but not in *scrm2* (ref. ^26^). *SCRM* expression levels were lower in *wsb* and upregulated by WSB upon induction of *iWSB-mVenus*, suggesting positive feedback regulation between *WSB* and *SCRM* (Fig. 6f,g). As *SCRM* was required for *FAMA* expression (Extended Data Fig. 1c) and the FAMA–SCRM complex directly binds to the *FAMA* promoter^18^, we monitored *FAMA* expression in *wsb* mutant and *WSB* overexpression lines. *FAMA* transcript levels were lower in *wsb* and upregulated by WSB (Fig. 6h,i). In *wsb proFAMA:GFP*, we detected a GFP signal only in GCs just after division, indicating that WSB is required for continued *FAMA* expression in GCs (Fig. 6j left panels). We rarely detected a GFP signal in inner tissues of *wsb proFAMA:GFP*, suggesting that *FAMA* expression in MCs almost fully depends on WSB (Fig. 6j right panels). Finally, we detected the binding of WSB to the *FAMA* promoter under high *WSB* overexpression conditions (using *iWSB-mVenus* treated with 0.01 or 10 µM oestrogen for ChIP-seq and ChIP–qPCR for the *FAMA* locus, respectively) (Fig. 6k), suggesting that WSB may weakly bind to the *FAMA* promoter. Collectively, these results suggest the existence of a positive feedback regulation between WSB and the FAMA–SCRM complex in both MCs and GCs. This feedback is required for the sustained, strong expression of the pioneer TF gene, *FAMA*, in GCs and MCs.

**Extended Data Fig. 9:**
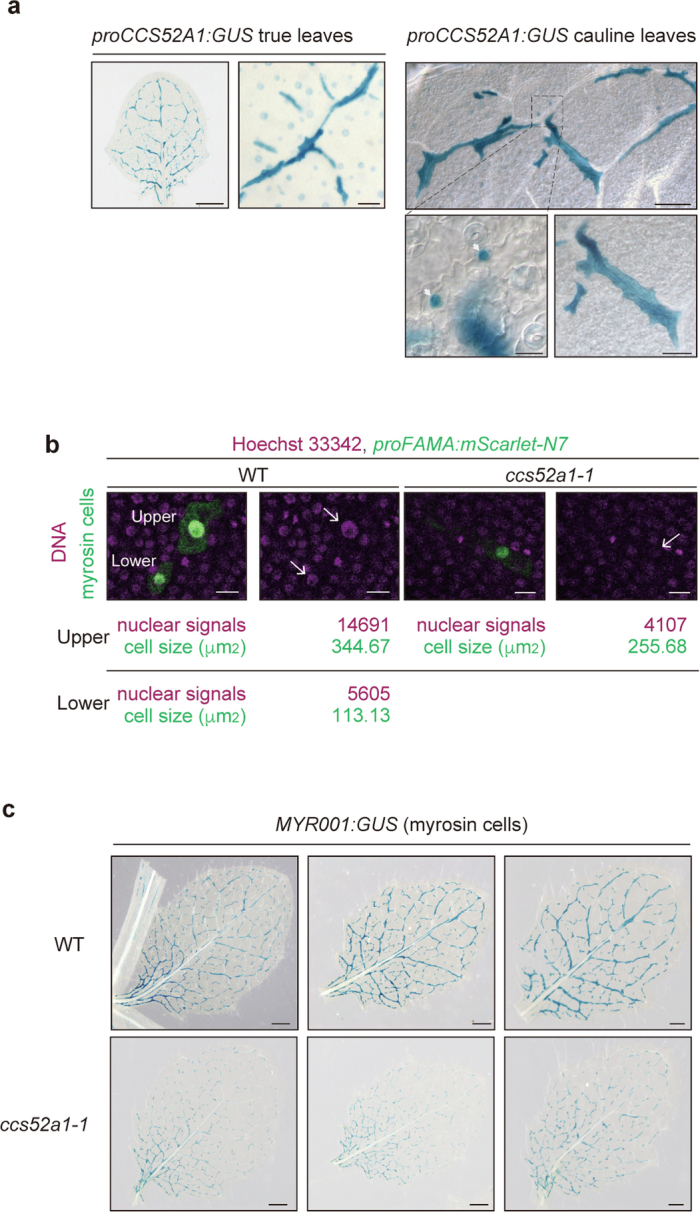
*CCS52A1* is required for the development of MCs. **a**, GUS staining of rosette (left) and cauline (right) leaves from WT Arabidopsis (Col-0) harboring *proCCS52A1:GUS* at 14 and 28 DAG, respectively. A strong signal was detected in MCs, a faint signal was detected in GCs, and moderate signal was detected in GMCs (arrows). **b**, Confocal images of nuclei stained with Hoechst 33342 for MCs of fifth true leaves from the WT and *ccs52a1-1* at 8 DAG. Signals of Hoechst 33342 fluorescence are correlated with DNA amounts (ploidy). MC nuclei were labeled with the *proFAMA:mScarlet-N7* marker. Samples were cleared by ClearSee and then stained with Hoechst 33342. Quantification of nuclear Hoechst 33342 signals and cell sizes using LAS X quantification mode. **c**, GUS staining of the seventh (left), eighth (middle), and ninth (right) true leaves from WT and *ccs52a1-1* seedlings at 15 DAG harboring the MC marker reporter *MYR001:GUS*. Note that MCs in *ccs52a1-1* are smaller than those in the WT. Scale bars, 500 µm (**a**, true leaves, left, **c**, left and middle); 200 µm (**c**, right); 100 µm (**a**, cauline leaves, upper); 50 µm (**a**, true leaves, right and **a**, cauline leaves, lower right); 20 µm (**a**, cauline leaves, lower left); 10 µm (**b**).

We then searched for factors that may promote cell and nuclear expansion, two characteristics of idioblast MCs. WSB bound to the promoter of *CELL CYCLE SWITCH PROTEIN 52 A1* (*CCS52A1*, or *FIZZY-RELATED 2* (*FZR2*)), which promotes endoreduplication and nuclear and cell expansion (Fig. 6e)^54–56^. In agreement with its role, *CCS52A1* was upregulated by WSB (Fig. 6l) and was also strongly expressed in MCs (Fig. 6m and Extended Data Fig. 9a). Furthermore, *CCS52A1* expression was downregulated in the *wsb-1* mutant (Fig. 6n), suggesting that high levels of CCS52A1 increase the ploidy, nuclear size and cell size of MCs. These three characteristics were diminished in *ccs52a1* mutants (Fig. 6o–q, Extended Data Fig. 9b,c and Supplementary Fig. 19). Collectively, these results suggest that the WSB–*CCS52A1* module is required for MC differentiation.

### WSB was co-opted for MC differentiation during evolution

**Fig. 7:**
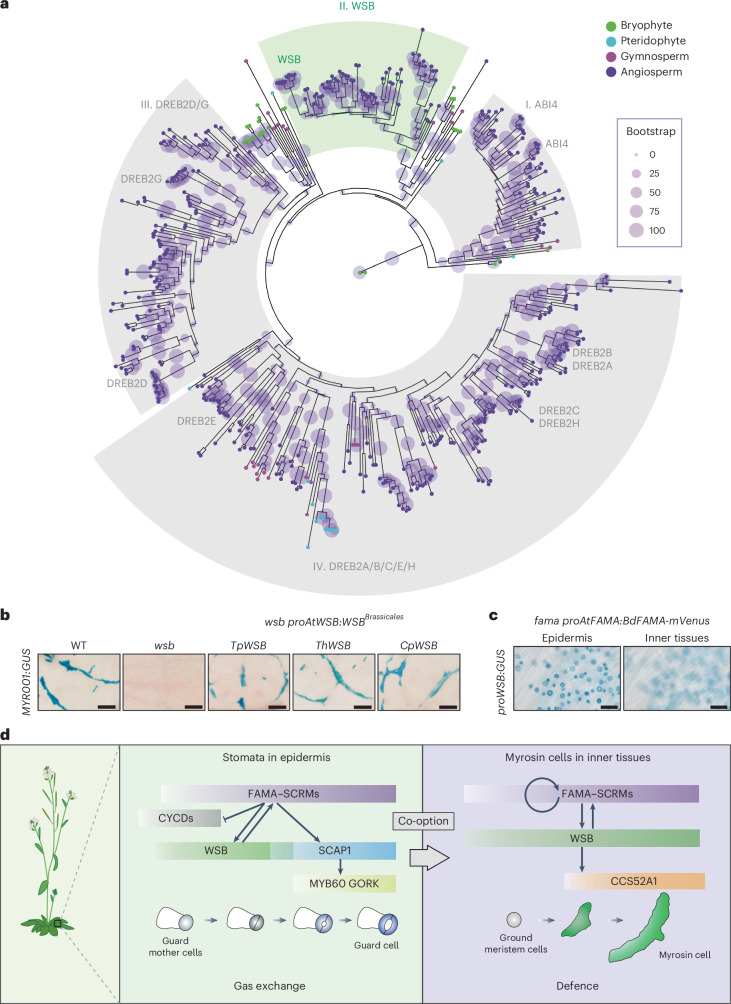
Hypothetical model for the co-option of the reduced transcriptional module, FAMA–*WSB*, during the evolution of Brassicales. **a**, Phylogenetic maximum-likelihood tree of Group IV AP2/ERF TFs. The sizes of light purple circles indicate the bootstrap value. See also the high-resolution tree in Supplementary Fig. 20. **b**, The expression of any one of three *WSB* homologues from Brassicales species rescues the loss of MC development seen in *wsb*. Each *WSB* homologue was driven by the *AtWSB* promoter in *wsb-3* harbouring the myrosin cell reporter *MYR001:GUS* (blue). True leaves of transgenic seedlings at 14 DAG were used for GUS staining. At least 2 independent T_2_ transgenic lines for each DNA construct were analysed. **c**, GUS staining of true leaves from *fama proAtFAMA:BdFAMA-mVenus* seedlings at 14 DAG, harbouring *proWSB:GUS*. The two panels are photographs of the same region in different focal planes. Left: photograph focusing on the epidermis. Right: photograph focusing on inner tissues. Note that the expression of *WSB* was only detected in stomatal-lineage cells. **d**, Proposed model. WSB (green) is required for the differentiation from GMCs to GCs. In cells from the stomatal lineage, FAMA–SCRMs (purple) inhibit ectopic divisions after the final division of the GMC, directly repressing the expression of *CYCD7* (grey) and *CDKB1;1* (ref. ^93^). In addition, FAMA–SCRMs directly activate the expression of *WSB*, and FAMA sequentially activates the expression of *SCAP1* (blue). These two TF genes are expressed in different expression windows (*WSB*, from GMC to young GC; *SCAP1*, from young GC to mature GC), but their expression windows also partially overlap. WSB and SCAP1 synergistically function in GC differentiation. WSB is required for the differentiation of ground meristem cells into MCs. FAMA–SCRMs directly activate *WSB* expression and then WSB directly activates *CCS52A1* (orange) for the differentiation of MCs. Ground meristem cells are stem cell-like cells in inner leaf tissues^24^. From genetic analyses, we propose that WSB suppresses the expression of *SCAP1* in MCs. The positive feedback loops of FAMA–*WSB* and WSB–*FAMA* may support the sustained and/or high expression of *WSB* in MC lineages. Scale bars, 100 µm (**b**) and 50 µm (**c**).

Our study revealed that WSB is required for the differentiation of both MCs and GCs, and for the expression of the pioneer TF gene *FAMA*. GCs are one of the oldest innovations of plants as they adapted to land, as evidenced by their presence in bryophytes, mosses and hornwort^31,32^. By contrast, MCs are present only in Brassicales plants^29^. Therefore, we hypothesized that WSB might not represent a Brassicales-specific TF but might instead have first been acquired for GC differentiation before being co-opted to form the MC differentiation pathway. To explore the evolutionary path of WSB, we generated a maximum-likelihood tree of 461 Group IV AP2/ERF TFs from 62 plant species (Fig. 7a and Supplementary Fig. 20). This high-resolution analysis revealed that, rather than a single subclass, as was previously reported^43^, Group IV AP2/ERF TFs form four distinct subclasses, I–IV. In Arabidopsis, the single-member WSB belongs to subclass II, supporting the notion that WSB has a unique biological function. Subclass II appears to be highly conserved in angiosperms, suggesting that plants acquired WSB before the emergence of Brassicales. Like other regulators of stomatal differentiation, WSB was lost secondarily in the seagrass *Zostera marina*, an astomatous plant (Fig. 7a and Supplementary Fig. 20)^57^. By contrast, subclass I, III and IV members are conserved, even in seagrass (Fig. 7a and Supplementary Fig. 20). These results indicate that WSB orthologues are intimately linked to the evolution of stomata. Collectively, our evolutionary analysis suggests that WSB was co-opted for MC differentiation during evolution.

**Extended Data Fig. 10:**
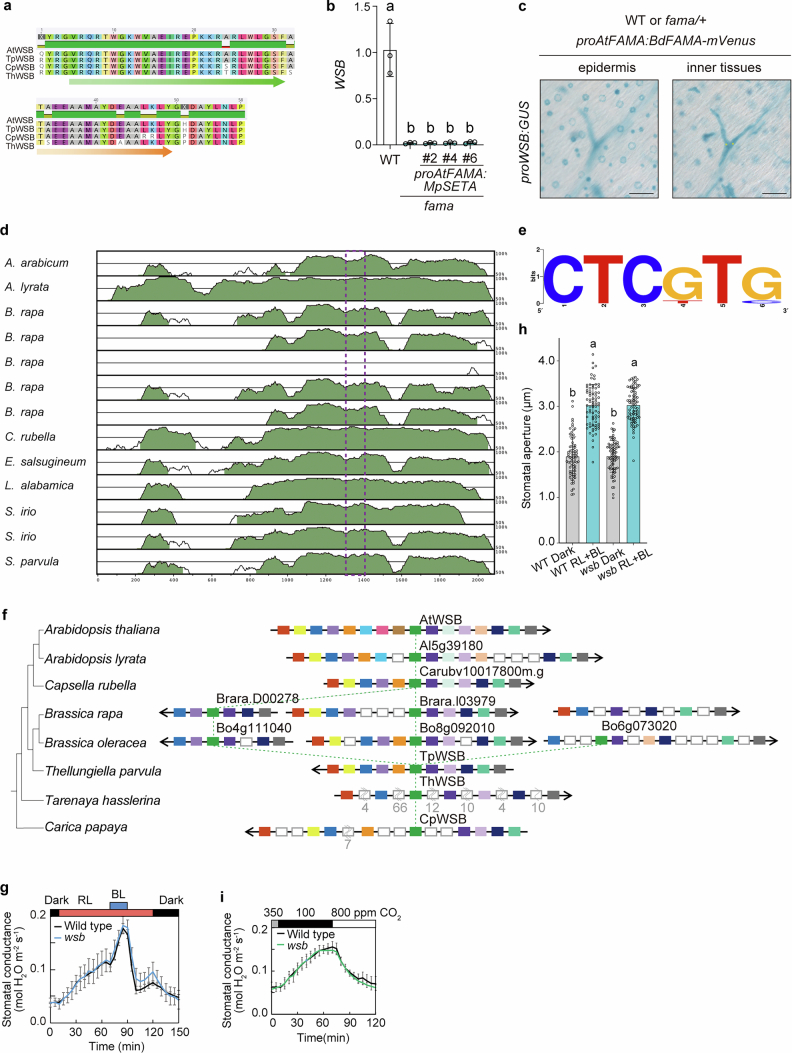
FAMA-binding sites in the *WSB* promoter are conserved in Brassicaceae. **a**, Alignment of the AP2/ERF domains of WSB and related proteins in Brassicales (AtWSB: *Arabidopsis thaliana*, TpWSB: *Thellungiella parvula*, CpWSB: *Carica papaya*, ThWSB: *Tarenaya hassleriana*). Green arrow, β-sheet; orange arrow, α-helical structures. **b**, RT-qPCR analysis of *WSB* transcript levels in whole seedlings at 12 DAG for the WT (Col-0), *fama*, and *fama proAtFAMA:MpSETA* (*n* = 3). Open circles represent individual data points. Data are means ± SD. Different lowercase letters indicate significant difference, as determined by one-way ANOVA test followed by Tukey-Kramer test (*p* < 0.05). **c**, GUS staining of true leaves from WT or *fama/FAMA proAtFAMA:BdFAMA-mVenus* seedlings at 14 DAG harboring *proWSB:GUS*. The two panels are photographs of the same region in different focal planes. Left, photograph focusing on the epidermis; right, photograph focusing on inner tissues. **d**, Pairwise alignment using mVISTA of the upstream promoter region of 13 *WSB* promoter sequences from Brassicales. The x-axis indicates the position in the upstream region of the *WSB* promoter, and the y-axis indicates the percentage conservation (50–100%). The number of conserved elements (green coloured) depends on the percentage identity ( > 70%) and length cutoffs (100 bp). The dashed purple box indicates the genomic region to which FAMA binds in Arabidopsis. **e**, WebLogo (https://weblogo.threeplusone.com/) of the core CTCGTG motif^60,61^. **f**, Synteny analysis of *WSB* orthologs. Gene lists from the eight plant species were obtained from the Plaza database (Dicot 4.5; http://bioinformatics.psb.ugent.be/plaza/). Genome synteny was detected by orthology ID in the Plaza database with evaluation of neighbouring genes (boxes in different colours) located in the genomic region of *WSB* orthologs (green coloured boxes). Detailed information is provided in Supplementary Table 11. **g**, Light-induced stomatal responses in the WT and the *wsb-1* mutant. Light-induced changes in stomatal conductance in intact leaves (true leaves from 4-week-old plants). Dark-adapted plants were illuminated with red light (RL: 300 µmol m^−2^ s^−1^) and blue light (BL: 10 µmol m^−2^ s^−1^) as indicated. Data are means ± standard error of the mean (SEM, *n* = 3; stomatal conductance [mol H_2_O m^-2^ s^-1^]). **h**, Light-induced stomatal opening on the epidermis. Epidermal strips from dark-adapted leaves (true leaves from 4-week-old plants) were illuminated with RL (50 µmol m^−2^ s^−1^) and BL (10 µmol m^−2^ s^−1^) for 2 h. Open circles represent individual data points. Data are means ± SD. (*n* = 75; stomatal aperture [µm]). Different lowercase letters indicate significant differences, as determined by ANOVA with Tukey’s test; *p* < 0.01. **i**, CO_2_-induced stomatal responses in the WT and the *wsb-1* mutant. CO_2_-induced changes in stomatal conductance in intact leaves (true leaves from 4-week-old plants) of dark-adapted plants. Ambient CO_2_ concentration was lowered from 350 to 100 ppm and then raised to 800 ppm as indicated. Data are means ± SEM (*n* = 3; stomatal conductance [mol H_2_O m^-2^ s^-1^]). Scale bars, 50 µm (**c**).

The presence of MCs is a synapomorphic characteristic of Brassicales^27^ (a characteristic derived from a common ancestor), which raises the question: When was WSB co-opted for MCs in the common ancestor of Brassicales? Synapomorphy posits that the MC-promoting function of WSB should be conserved across Brassicales plants. To assess this prediction, we compared the sequence of the DNA-binding domain in WSB and WSB-like proteins and performed complementation tests by expressing the coding sequences of *WSB* orthologues under the control of the Arabidopsis *WSB* promoter. The DNA-binding domain is indeed highly conserved among WSB orthologues from Brassicaceae (Arabidopsis and salt cress *Thellungiella parvula*), Cleomaceae (giant spider-flower *Tarenaya hassleriana*) and basal Brassicales plants, including Caricaceae (papaya *Carica papaya*) (Extended Data Fig. 10a). In addition, expressing *TpWSB*, *ThWSB* or *CpWSB* from the Arabidopsis *WSB* promoter rescued the loss of MCs seen in *wsb* (Fig. 7b), suggesting that the MC-promoting function of WSB is deeply conserved in Brassicales. We obtained the same results when driving the coding sequence for *WSB* orthologues in non-Brassicales from the Arabidopsis *WSB* promoter in *wsb* (Supplementary Fig. 21). Taken together, these results suggest that the co-option of WSB might have occurred in the common ancestor of Brassicales.

Next, to clarify the evolution of the FAMA–*WSB* module, we examined the induction of *WSB* by expressing the coding sequences of *FAMA* orthologues under the control of the Arabidopsis *FAMA* (*AtFAMA*) promoter in an Arabidopsis *fama* mutant. *MpSeta*, a bHLH Ia gene in the astomatous plant *Marchantia polymorpha* and the closest homologue to *AtFAMA*, *AtMUTE* and *AtSPCH*^58^, did not induce *WSB* expression (Extended Data Fig. 10b). Notably, *BdFAMA* from purple false brome (*Brachypodium distachyon*) induced *WSB* expression in the epidermis but not in inner tissues (Fig. 7c and Extended Data Fig. 10c). These results can be explained by a previous report stating that *BdFAMA* driven by the *AtFAMA* promoter in *fama* is expressed in the epidermal layer but not in inner tissues^59^. Moreover, the expression of *AtFAMA* in inner tissues requires positive feedback regulation by AtFAMA itself^25,26^. BdFAMA may not activate the *AtFAMA* promoter, resulting in the loss of *WSB* in inner tissues. These results suggest that the positive feedback-dependent expression of *FAMA* in inner tissues was acquired during evolution.

As FAMA directly activated *WSB* expression (Fig. 2), we explored the extent of conservation at *WSB* promoter regions in Brassicales and observed high conservation of a ~500-bp fragment in the *WSB* promoter regions across Brassicales plants, which includes both the FAMA-binding site and CTCGTG motifs (Extended Data Fig. 10d,e)^60,61^. In addition, synteny analysis suggested that the arrangement of the *WSB* locus in its genomic region is conserved in Brassicales (Extended Data Fig. 10f and Supplementary Data 11). Together, these results suggest that the FAMA–*WSB* transcriptional module may be conserved in this plant order. Overall, we propose that Brassicales plant species co-opted the FAMA–*WSB* module for the evolution of the myrosinase-glucosinolate defence strategy.

## Discussion

Despite the importance of GCs for gas exchange and MCs for plant defence, no component critical for MC and GC differentiation has been identified under the control of FAMA. Here we rediscovered *WSB* and identified *SCAP1* as direct targets of FAMA (Figs. 1, 2 and 4). *WSB* is expressed in both MCs and GCs, while *SCAP1* is specifically expressed in GCs (Figs. 2 and 4). *WSB* is required for the expression of myrosinase genes (Fig. 3). *wsb* mutants lack MCs, while the *wsb scap1* double mutant has few normal GCs (Figs. 3 and 5). In addition, ChIP-seq analysis revealed that WSB governs the transcriptional networks controlling the differentiation of both MCs and CGs, with WSB forming a positive feedback circuit with the FAMA–SCRM complex, while the WSB–*CCS52A1* module is a key module specific to the differentiation of MCs (Fig. 6). Finally, an evolutionary analysis revealed that WSB is conserved across stomatous angiosperms (Fig. 7a). It was also previously reported that MCs are Brassicales-specific idioblast cells^29^. From these results, we propose that the conserved and reduced FAMA–*WSB* transcriptional module was co-opted for MC differentiation during evolution (Fig. 7d). Below, we discuss the conserved stomatous transcriptional module, FAMA–*WSB–SCAP1*, and the co-option and neofunctionalization of the stomatal executors, FAMA and WSB, for defence against herbivores in Brassicales.

To our knowledge, *wsb* is the first mutant to be reported in which no MCs develop without being accompanied by dwarfism (Fig. 3). By contrast, *fama* mutants lack MCs but also showed strong dwarfism because of their absence of stomata, thus limiting CO_2_ fixation. In *wsb*, in addition to *TGG*s, 168 MC-expressed genes (for example, *HIPP20* and *WRKY23*) whose functions in MC are unknown were downregulated (Fig. 3h). Therefore, *wsb* is a useful tool to explore as yet undiscovered functions of MCs besides their role in plant defence. MCs differentiate from ground meristem cells, which are the stem cells in leaf primordia. As there is no good reporter gene for these ground meristem cells, it is unclear when and how the differentiation of MCs might stop or stall after the ground meristem cell stage in *wsb* or *fama* mutants. Notably, in our study, MCs in *WSB*-knockdown lines, in which WSB function and MCs were not completely lost, ectopically expressed *SCAP1* (Extended Data Fig. 7d). The characterization of these abnormal MCs in *WSB*-knockdown lines may shed light on the origin, trajectory and cell fate bifurcation of MC differentiation. Recently, it was reported that MCs exist in Arabidopsis roots during secondary growth and might differentiate from phloem parenchyma cells and/or companion cells^62^. It is an open question whether *SCAP1*-positive cells in *WSB*-knockdown lines are phloem parenchyma cells (specifically, companion cells) in leaves, even though the development of vascular tissues is different between leaves and roots. In addition, scRNA-seq or single-nucleus RNA sequencing analysis of leaf primordia, including MCs and phloem lineages, may provide useful information about the cell lineages and upstream factors of *FAMA* in inner tissues surrounding future vascular tissues. From the viewpoint of applied science, *WSB* may be a good target for gene editing to generate Brassicales vegetables/crops without pungency, as gene editing of multiple homologous genes, such as the *TGG*s, is time-consuming.

In this study, we showed that WSB and SCAP1 are synergistically required for the differentiation of GCs. It was previously reported that a quadruple mutant defective in *ERF49–52* (*ERF49*, *ERF50*, *WSB* (also known as *ERF51* and *DREB2F*) and *ERF52*) did not exhibit highly penetrant stomatal phenotypes^20^. Taken together, these results suggest that WSB did not function redundantly with homologous Group IV AP2/ERF TFs but rather synergistically with SCAP1, which belongs to the DOF family of TFs. Consistent with this idea, our phylogenetic analysis revealed that only WSB belongs to subclass II of the Group IV AP2/ERF TFs (Fig. 7a). These results indicate that FAMA executes GC differentiation by deploying the TFs WSB and SCAP1 (Fig. 7d).

Consistent with the observation that *SCAP1* expression is sustained in mature GCs, beyond its role in cell differentiation, SCAP1 is required for stomatal movement through the direct induction of *GATED OUTWARDLY-RECTIFYING K*^*+*^ *CHANNEL* (*GORK*) and *MYB60* (ref. ^22^). In agreement with the narrow expression window of *WSB* in GMCs and GCs just after division, light- or CO_2_-induced stomatal movement in *wsb* mutants was comparable to that in the WT (Extended Data Fig. 10g–i), suggesting functional differences between SCAP1 and WSB. Thus, whether WSB and SCAP1 share downstream targets and/or have specific targets are open questions. The transcriptional cascade starting from FAMA that includes *CYCD7*, *WSB* and *SCAP1* is a good model in which to study the timely expression of developmental regulators in plants. FAMA represses *CYCD7* and activates *WSB* and *SCAP1*, sequentially and with different timing (Fig. 7d). Notably, both *WSB* and *SCAP1* are direct targets of FAMA; however, *SCAP1* was slower to respond than *WSB* (Figs. 2 and 4). In addition, it was recently reported that ectopic induction of *FAMA* in embryos induced the ectopic expression of *WSB* but not of *SCAP1* (ref. ^21^). WSB did not suppress *SCAP1* expression in the stomatal lineages (Supplementary Fig. 11). These results suggest that unknown genetic and/or epigenetic barriers may exist that modulate the induction of *SCAP1* by FAMA in the stomatal lineage. We do not exclude the possibility that additional transcriptional activators are required for the induction of *WSB* and *SCAP1* in addition to FAMA. Consistent with this prediction, the repressive histone mark trimethylation of lysine 27 on histone H3 may modulate *SCAP1* expression^63^. To induce the expression of *SCAP1* by FAMA, the previous dilution of repressive histone marks may be needed. Further studies are required to clarify the role of epigenetic regulation at the *SCAP1* locus in the stomatal lineage. A set of 8,484 direct target genes were recently identified for FAMA^18^; therefore, other direct FAMA targets may play critical roles in other aspects of stomatal development and function.

In addition to promoting GC differentiation and fine-tuning GC divisions, a third role for FAMA in GC development is fate maintenance. FAMA interacts with RETINOBLASTOMA-RELATED (RBR) and HISTONE ACETYLTRANSFERASE OF THE CBP FAMILY 1 (HAC1), and the FAMA–RBR and FAMA–HAC1 complexes both maintain the irreversible fate of GCs through histone modifications^18,44,64,65^. For example, when the FAMA–RBR interaction is abolished, paired GCs differentiate inside of existing GCs and lobed GCs form. We did not observe such abnormal GCs on the epidermis of *wsb scap1*, suggesting that WSB and SCAP1 are not primarily involved in this third FAMA role. As with Arabidopsis FAMA, FAMA in rice (*Oryza sativa*) promotes this GC differentiation^66,67^. BdFAMA mainly promotes GC differentiation, although BdFAMA and BdMUTE share functions^59^. These results suggest that the promotion of GC differentiation is the primary role of FAMA in angiosperms. A stomatal function for WSB and SCAP1 in other species remains to be determined.

Genome-wide profiling of WSB targets revealed that WSB contributes to the full expression of *SCRM*. Reporter analyses revealed that WSB is required for *FAMA* expression (Fig. 6). *FAMA* was clearly downregulated in *wsb*, while *SCRM* was weakly downregulated in *wsb*. This difference may be explained by the broad expression pattern of *SCRM*, which is expressed in early stomatal lineages^17^ and mesophyll cells^26^, in addition to GMCs and GCs. The binding strength of FAMA to the *WSB* locus is comparable to that at the *EPF1* locus, to which FAMA is reported to bind (Fig. 2c). The binding strength of SCRM at the *WSB* locus is comparable to that at the *TMM* locus, to which SCRM is reported to bind (Extended Data Fig. 2e). Notably, the binding of WSB to the *FAMA* locus is very weak because such binding can only be detected when *WSB* is overexpressed, and it was not detected by ChIP-seq analysis for WSB (Fig. 6k). These results suggest that the binding of WSB to the *FAMA* locus is transient and/or that WSB indirectly binds to the *FAMA* locus, that is, it binds through other protein(s). Further experiments are required to fully elucidate the detailed binding mode of WSB to the *FAMA* locus.

Analyses of fluorescent reporters^14,17^ and scRNA-seq^53^ indicated that the FAMA–SCRM complex exists until GCs mature, when WSB disappears (Fig. 4). The positive feedback circuit between WSB and FAMA–SCRM underpins the sustained expression of *FAMA* and *SCRM* in the stomatal lineage (Fig. 7d). We detected weak GFP signals in GCs of *wsb proFAMA:GFP* just after their division (Fig. 6j), suggesting that *FAMA* expression is not fully dependent on WSB. Combined with the results that showed that *WSB* is not expressed in the epidermis of *fama*, we conclude that FAMA is an upstream factor of *WSB*. In the epidermis, it was previously reported that *MUTE*, which is expressed in meristemoids and early GMCs, directly induces *FAMA* expression in stomatal-lineage cells^51^. Therefore, these results suggest that MUTE induces *FAMA* in late meristemoids and/or early GMCs, which in turn induces *WSB* in GMCs and GCs just after division; finally, the FAMA–WSB feedback sustains *FAMA* expression in young/maturing GCs. Around the time when *WSB* expression declines, FAMA may start to induce the expression of *SCAP1*, whose encoded protein subsequently induces *MYB60* and *GORK* expression, key factors for stomatal maturation. It remains an open question how WSB disappears during stomatal differentiation even though *FAMA* is still expressed. One hypothesis is that unknown stomatal lineage-specific factors might degrade WSB. Alternatively, epigenetic regulators such as HAC1 may be required for the sustained expression of *FAMA*^18^. Future studies will shed light on how WSB and epigenetic regulators work together or independently to maintain sustained *FAMA* expression.

How did plants adjust the expression of *WSB* in MCs during evolution? In cells from the stomatal lineage, *FAMA* expression is MUTE dependent and FAMA independent^51^, and importantly, *MUTE* is not expressed in MCs^25^. Our comparative analysis of AtFAMA and BdFAMA indicates that a positive feedback regulation of FAMA by itself is essential for *FAMA* and subsequent *WSB* expression in MCs (Fig. 7c)^25,26^. How Brassicales plants established the positive feedback regulation of FAMA is an important question for further investigation. For example, the evolution of *cis* elements necessary for FAMA binding in the *FAMA* promoter and the 3D structure of the FAMA–SCRM complex bound to these *cis* elements should be examined. We also do not exclude the possibility that other inner tissue-specific phytohormones and/or TFs contribute to the expression of *WSB* with the FAMA–SCRM complex.

One remarkable difference between GCs and MCs is their cell size^29,68^. Here we showed that a positive regulator of cell and nuclear expansion, *CCS52A1*, was directly induced by WSB and was more strongly expressed in MCs than in GCs. Indeed, loss of CCS52A1 function resulted in smaller cells and nuclei, as well as lower ploidy, for mature MCs (Fig. 6). These results suggest that the strong and/or sustained induction of *CCS52A1* is a key cellular event for the differentiation of MCs (Fig. 7d). To clarify the detailed role of *CCS52A1* in the differentiation of MCs, live imaging of MC development and their ploidy change using the histone H2B reporter (for example, H2B-GFP) in WT and *ccs52a1* mutants is needed in future studies. In addition, comparative transcriptome studies of MCs from WT and *ccs52a1* are needed to elucidate the comprehensive role of CCS52A1 in MCs using single-cell analysis approaches. Further analysis of other WSB targets will provide a detailed molecular mechanism of MC differentiation.

In addition to co-option, we also revealed the neofunctionalization of *FAMA* and *WSB* during evolution (Fig. 7d). First, the neofunctionalization of *FAMA* led to its essential role in its own activation in the MC lineage. We do not exclude the possibility that the positive feedback regulation of *FAMA* also occurs in the stomatal lineage, but it is not essential because some cells in *fama-1* tumours express the *FAMApro:GFP* reporter^14^. Second, the neofunctionalization of *WSB* is reflected by its prolonged expression window in MC lineages. Prolonged expression of *WSB* strongly induces the expression of *CCS52A1* for MC differentiation (increased ploidy and cellular and nuclear expansion) (Fig. 6) and repressed *SCAP1* expression to prevent MCs from acquiring stomatal characters (Extended Data Fig. 7d). The stomatal-lineage expression window of *SCAP1* in *WSB*-knockdown lines (amiRNA*-WSB*) is indistinguishable from that in the WT, suggesting that the inhibitory effects of WSB toward *SCAP1* are MC lineage specific (Supplementary Fig. 11). For the prolonged expression of *WSB*, promoter activity and/or WSB stability may be critical. Indeed, *WSB* homologues outside Brassicales driven by the *AtWSB* promoter rescued the absence of MCs in the *wsb* mutant (Supplementary Fig. 21), suggesting that the *AtWSB* promoter is important for the development of MCs and neofunctionalization of *WSB*. Analyses of the evolution of *cis* regulatory elements in the *AtWSB* promoter are needed for future studies. We cannot address a possible neofunctionalization of WSB protein stability in Brassicales, as we used codon-optimized sequences for each *WSB* homologue tested, which may improve translational efficiency. These two neofunctionalization events of *FAMA* and *WSB* are connected by the transcriptional layers between FAMA and WSB. Taken together, these results suggest that the positive feedback-mediated sustained or high expression of *WSB* (*WSB* dynamics) in the MC lineage may have driven the evolution of MCs. Thus, we provide evidence here for the co-option and the neofunctionalization of stomatal executors, FAMA and WSB, for defence against herbivores in Brassicales.

## Methods

### Plant materials and growth conditions

The Arabidopsis (*Arabidopsis thaliana*) accession Columbia-0 (Col-0) was used for all lines except for *MYR002:GUS* (in C24). The T-DNA insertion mutants SALK_100073 (*fama-1*), SALK_003155 (*scrm*) and SAIL_808_B10 (*scrm2-1*) were obtained from the Arabidopsis Biological Resource Center (ABRC) at Ohio State University. The *iFAMA MYR001:GUS*^26^, *MYR001:GUS*^33^, *proTGG2:VENUS-2s*^46^ and *syp22* (ref. ^36^) germplasms were reported previously. *proFAMA:FAMA-myc*^44^ was provided by Dr Dominique Bergmann (Stanford University). *scrm scrm2* (ref. ^17^), *scrm-D*^17^ and *proSCRM:SCRM-GFP*^17^ were provided by Dr Keiko Torii (University of Texas at Austin). *scap1* (ref. ^22^) and *proSCAP1:GUS*^22^ were provided by Dr Juntaro Negi (Kyushu University). *MYR002:GUS* (*proWRKY23:GUS*^69^) was provided by Dr Tom Beeckman (Ghent University). *proFAMA:GFP*^64^ was provided by Dr EunKyoung Lee and Dr Abel Rosado (University of British Columbia). *ccs52a1-1* (refs. ^54,56^) and *proCCS52A1:GUS*^54,56^ were provided by Dr Masaaki Umeda (Nara Institute of Science and Technology). *ccs52a1-5* (ref. ^55^) was provided by Dr Eva Kondorosi (Institute of Plant Biology) and Dr Peter Mergaert (Institute for Integrative Biology of the Cell). *fama proAtFAMA:MpSETA*^58^ was provided by Dr Tomoo Shimada (Kyoto University). *wsb* mutants were generated by CRISPR/Cas9-mediated gene editing (see below). The *wsb scap1* double mutant, *wsb-1*/*wsb-2* F_1_ plants and *wsb-1*/*wsb-3* F_1_ plants were generated by crossing. Mutants and transgenic plants used in this study are listed in Supplementary Data 12. Seeds were surface sterilized with 70% (v/v) ethanol and then sown onto Murashige and Skoog (MS) medium (Wako) solidified with 0.5% (w/v) gellan gum (Wako) and containing 1% (w/v) sucrose. The seeds were stratified at 4 °C for 2–5 days to break dormancy and grown at 22 °C for 20 days under continuous light (100 µE s^−1^ m^−2^) or long-day conditions (16 h light/8 h dark) in a plant growth chamber (BiOTRON, LPH-411SP, NIPPON MEDICAL and CHEMICAL INSTRUMENTS) with fluorescent light tubes. Humidity in the plant growth chamber was kept at 40–50%. After 14–21 days, plants were transferred to pots containing vermiculite and Metro-Mix for subsequent growth. The humidity in the plant culture room was kept at 40–50%.

### Plasmid construction and transgenic Arabidopsis lines

The Gateway cloning system (Life Technologies) was used for plasmid construction. For transcriptional *GUS* constructs, a 1,444-bp *WSB* promoter fragment, a 1,703-bp *SCAP1* promoter fragment (*proSCAP1 67bp_del*) and a 1,704-bp *HIPP20* promoter fragment were individually cloned into pENTR D-TOPO (Invitrogen by Thermo, K240020). The resulting subcloned promoters were individually recombined into the binary vector pBGWFS7 or pHGWFS7 using LR reactions (LR Clonase II Enzyme mix, Invitrogen by Thermo, 11791100). For translational C-terminal fusion constructs of WSB, the coding sequence of *mVenus* or *mTurquoise2* was inserted in-frame and downstream of a 2.2-kb *WSB* genomic fragment (including 1.4 kb of the promoter sequence) without the stop codon and with a GGSG linker sequence. For translational C-terminal fusion constructs of SCAP1, the coding sequence of *mVenus* or *mCherry* or *mTurquoise2* was inserted in-frame and downstream of a 2.7-kb *SCAP1* genomic fragment (including 1.8 kb of promoter sequence) without the stop codon and with a GGSG linker sequence. After cloning into pENTR D-TOPO, the constructs were recombined into the binary vector pGWB601 (ref. ^70^) using an LR reaction. For the inducible overexpression construct of *WSB-mVenus*, the coding sequence of *mVenus* was inserted in-frame and downstream of the 0.9-kb *WSB* coding sequence without a stop codon and with a GGSG linker sequence. After cloning into pENTR D-TOPO, the constructs were recombined into the binary vector pMDC7 (ref. ^71^) using an LR reaction. For the *proWSB:TpWSB*, *proWSB:CpWSB*, *proWSB:ChWSB*, *proWSB:AmWSB*, *proWSB:BdWSB* and *proWSB:OrWSB* constructs, the coding sequences of *TpWSB*, *CpWSB*, *ChWSB*, *AmWSB*, *BdWSB* and *OrWSB* codon-optimized for Arabidopsis were synthesized (Eurofins Japan) and cloned into pENTR D-TOPO. The resulting constructs were then recombined into the binary vector R4pGWB601 with pENTR 5′-TOPO *proWSB* using an LR reaction. For the *proFAMA:H2B-tdTomato*, *proFAMA:mScarlet-N7*, *proFAMA:mCherry-RCI2A*, *proFAMA:CYCD7-mVenus* and *proFAMA:BdFAMA-mVenus* constructs, a 3,102-bp *FAMA* promoter fragment was cloned into pENTR 5′-TOPO, and the coding sequences of *mScarlet-N7*, *mCherry-RCI2A*, *CYCD7-mVenus* and *BdFAMA-mVenus* (*BdFAMA* was synthesized by Eurofins Japan) were cloned into pENTR D-TOPO, respectively. pENTR D-TOPO *H2B-tdTomato* was provided by Dr T. Goh (Nara Institute of Science and Technology). The resulting constructs were recombined into the binary vector R4pGWB601 (ref. ^70^) using an LR reaction. For *proUBQ11:mCitrine-RCI2A*, the *UBQ11* promoter was cloned into pENTR 5′-TOPO, and the coding sequence of *mCitrine-RCI2A* was cloned into pENTR D-TOPO. The resulting constructs were recombined into the binary vector R4pGWB601 (ref. ^70^) using an LR reaction. Two artificial microRNAs (amiRNAs) against *WSB* were designed using the WMD3-Web MicroRNA Designer (http://wmd3.weigelworld.org/cgi-bin/webapp.cgi)^72^ and amplified using the following primers from the pRS300 vector^72^: amiRNA-F, amiRNA-R, WSB_amiRNA3_1, WSB_amiRNA3_2, WSB_amiRNA3_3, WSB_amiRNA3_4, WSB_amiRNA6_1, WSB_amiRNA6_2, WSB_amiRNA6_3 and WSB_amiRNA6_4. The amplified amiRNA-*WSB* DNA fragment was cloned into the pENTR D-TOPO plasmid before being recombined into binary vector pFAST-R02 (ref. ^73^) using an LR reaction to generate amiRNA*-WSB*. For transcriptional *GUS* constructs, a *MYB60* promoter fragment (1,000 bp), a *KAT1* promoter fragment (2,000 bp), a *TET4* promoter fragment (1,000 bp) and an At1g23170 promoter fragment (1,398 bp) were cloned into pENTR D-TOPO (Invitrogen by Thermo, K240020). The resulting subcloned promoters were individually recombined into the binary vector pBGWFS7 or pHGWFS7 using LR reactions. All binary constructs were transformed individually into *Agrobacterium tumefaciens* strain GV3101. Plants were transformed with *Agrobacterium* cultures harbouring each binary vector using the floral-dip method^74^. T_1_ seedlings were selected on medium containing 10 mg l^−1^ BASTA or 25–50 mg l^−1^ hygromycin B, or the FAST-R reporter^73^. Mutants and transgenic plants used in this study are listed in Supplementary Data 12 and the primer sets are listed in Supplementary Data 13.

### Oestrogen treatment

Estradiol (Sigma-Aldrich, E8875-1G) was dissolved in 70% (v/v) ethanol to 10 mM concentration and used at 10 µM (1,000× dilution) or 0.01 µM (1,000,000× dilution, only for ChIP-seq experiments using *iWSB-mVenus* lines) as the final working concentration in water. For mock treatment, a 0.07% (v/v) ethanol solution was used. Seeds were sown onto MS medium (Wako) solidified with 0.5% (w/v) Gellan gum (Wako) and containing 1% (w/v) sucrose; seedlings at 4 DAG were transferred to water containing 10 µM oestrogen or water with 0.07% (v/v) ethanol, vacuumed at least three times and incubated for the indicated times.

### Generation of *wsb* mutants by CRISPR/Cas9

To generate *wsb* mutants by CRISPR/Cas9, two single guide RNAs (sgRNAs) were manually designed to target the sequence encoding the DNA-binding domain of WSB. The sgRNAs fused to the Arabidopsis *tRNA-Gly* gene were amplified using pGTR (Addgene, plasmid 63143)^75^ as PCR template and utilizing primers listed in Supplementary Data 13. The resulting PCR products harbouring the two sgRNA sequences were cloned into the pKI1.1R vector (Addgene, plasmid 85808)^48^ digested with AarI using NEBuilder HiFi enzyme (NEB). pKI1.1R harbouring the two sgRNAs was transformed into Arabidopsis Col-0 by the floral-dip method using *Agrobacterium* strain GV3101 (ref. ^74^). T_1_ transformants and T_2_ transgenic plants without the CRISPR/Cas9 construct were selected using the FAST-R reporter^73^.

### GUS staining and microscopy

Samples were first placed into ice-cold 90% (v/v) acetone for 15–30 min and then into β-glucuronidase (GUS) staining solution containing 0.5 mg ml^−1^ X-Gluc (Gold Biotechnology, G1281C5), 0.1 M sodium phosphate buffer (pH 7.0), 10 mM EDTA, 0.5–5 mM potassium ferricyanide, 0.5–5 mM potassium ferrocyanide and 0.1% (v/v) Triton X-100. Samples were vacuum infiltrated with GUS staining solution before being incubated at room temperature for 2–24 h^26^. Tissue sectioning was performed as described previously^39^. The sections were stained with 0.01% (w/v) toluidine blue (Wako Chemicals). Representative images were captured under an AX-70 light microscope (Olympus) and an AXIO Zoom V16 (Zeiss) microscope.

### Confocal laser-scanning microscopy

Fluorescence micrographs were obtained with a confocal laser-scanning microscope (Leica SP8 FALCON or STELLARIS 5) using dry objectives, a pulsed laser (440 nm), a diode laser (448 nm), a white-light laser (WLL), a HyD/HyD S detector and a gating system. The WLL allows for time-gated acquisition (gating system) to remove the autofluorescence from chlorophylls. The laser wavelengths used were mainly 440 nm (mTurquoise2), 488 nm (GFP), 514 nm (mVenus) and 552 nm (mCherry). For multicolour imaging, the sequential mode was used. The images were analysed and processed using LAS X (https://www.leica-microsystems.com/products/microscope-software/p/leica-las-x-ls/) (Leica), Fiji (https://fiji.sc/) and Photoshop 2021 (Adobe) software. To observe inner tissues, ClearSee solution (Fujifilm)^76^ was used. The samples were fixed overnight in 4% (w/v) paraformaldehyde in phosphate-buffered saline. Samples were cleared in ClearSee for at least 1 week. Before observations, SR2200 was added at a 5,000× dilution (SCRI Renaissance Stain 2200, Tokyo Future Style) for the staining of cell walls, or 80 μg ml^−1^ Hoechst 33342 solution (DOJINDO) for DNA staining. For quantification of stomatal features, cell peripheries were visualized following staining with 1 mg ml^−1^ propidium iodide (Molecular Probes, P1304MP). Methods for the staining of cell outlines are provided in Supplementary Data 14. The same confocal laser-scanning microscope settings were used when comparing the WT and mutants.

### RNA-seq analysis

Libraries for RNA‐seq were prepared using the Breath Adapter Directional sequencing method^77^. The libraries were sequenced on a Next‐Seq 500 instrument (Illumina). Mapping to the Arabidopsis reference genome (TAIR10) was conducted using Bowtie with the following options ‘‐‐all ‐‐best ‐‐strata ‐‐trim5 8’. The number of reads mapped to each gene was counted^77^. After normalization, the FDR and fold-change were calculated using the edgeR package in R. Differentially expressed genes were identified (FDR < 0.05). The data have been deposited at the DNA Data Bank of Japan (DRA013687).

### RT–qPCR

Leaves or stems were frozen in liquid nitrogen immediately after sample collection. An RNeasy Plant Mini kit (Qiagen, 74104) was used to extract total RNA. An RNase-Free DNase set (Qiagen, 79254) was used to eliminate genomic DNA contamination in RNA samples. Reverse transcription was performed using PrimeScript RT Master Mix (Takara, RR036A). Quantitative PCR was performed as described previously using FastStart Essential DNA Green Master Mix (Roche, 6924204001)^78^. Arabidopsis *ACT2* was used as the internal reference transcript. Each experiment was performed at least three times. The relative expression level of each gene was calculated using the 2^−ΔΔCT^ method^79^. Primers are listed in Supplementary Data 13.

### ChIP–qPCR experiment

For ChIP–qPCR, ChIP was carried out as described previously^80^. Samples were collected from seedlings at 4 DAG. For each sample, 300–600 mg of fresh seedling tissue was crosslinked in 1% (w/v) formaldehyde for 15 min. After quenching the crosslinking with 0.93% (w/v) glycine for 5 min, tissues patted dry with Kimwipes were frozen in liquid nitrogen and kept at −80 °C until use. Tissues were ground to a fine powder with an ice-cold mortar and pestle. Using nuclear extraction buffer, chromatin was isolated from the nuclear extracts. Fragmentation was conducted using a UD-201 Ultrasonic Disruptor sonicator (TOMY) or Bioruptor II (BMBio). After preclearing, the indicated antibodies were added and the mixtures were rotated overnight at 4 °C. The following antibodies were used: anti-Myc (sc-40 X, Santa Cruz Biotechnology, 3 µl per sample) and anti-GFP (SAB4301138, SIGMA, 5 µl per sample). For immunoprecipitation to capture DNA–protein complexes, Dynabeads with Protein A or G (Thermo Fisher) were used. Beads were washed in low-salt buffer and 250 mM LiCl buffer, and DNA was eluted from the beads overnight at 65 °C. The resulting DNA was purified using a QIAquick PCR Purification kit (Qiagen). DNA was quantified on a LightCycler 480 (Roche) instrument using FastStart Essential DNA Green Master Mix (Roche). The ratio of ChIP to input DNA (% input) was compared on the basis of the reaction threshold cycle for each ChIP sample compared with a dilution series of the corresponding input sample. Relative values were normalized to the negative control locus of the *TA3* retrotransposon (At1g37110)^80^. At least three independent experiments were performed. Primers are listed in Supplementary Data 13.

### ChIP-seq experiments

ChIP-seq was performed as previously described with slight modifications^81^. Two grams of *iWSB-mVenus* seedlings at 3 DAG were bathed in 0.01 µM estradiol in water for 24 h. Samples were then rapidly frozen in liquid nitrogen and stored at −80 °C until use. The frozen tissues were thoroughly ground using a mortar and pestle to obtain a fine powder. Chromatin was then fixed and isolated using a nuclei isolation buffer (10 mM HEPES, 1 M sucrose, 5 mM KCl, 5 mM MgCl_2_ and 5 mM EDTA) containing 1% (w/v) formaldehyde (Thermo Scientific). After chromatin fragmentation using an ultrasonicator (Covaris M220), immunoprecipitation of the resulting chromatin fragments was carried out using anti-GFP antibodies (SAB4301138, Sigma, diluted 1:1,000) and Dynabeads protein A (Thermo Scientific) at 4 °C. The validity of the antibodies was confirmed by the suppliers and by immunoblot analysis in the laboratory before use. Following immunoprecipitation, DNA was purified using a DNA Cleanup kit (New England Biolabs). The resulting DNA was used as a template to generate a sequencing library using a ThruPLEX DNA-seq kit (Rubicon Genomics), following manufacturer instructions. The immunoprecipitated fraction was analysed using a NovaSeq 6000 instrument (Illumina). The resulting FASTQ file underwent quality assessment using FastQC (v.0.11.7) (http://www.bioinformatics.babraham.ac.uk/projects/fastqc/); the raw reads were subjected to trimming using Trimmomatic (v.0.38)^82^. The reads were mapped to the Arabidopsis TAIR10 genome using Bowtie2 (v.2.3.4.2)^83^. Peak calling was performed with MACS2 (v.2.2.6)^84^ using the callpeak command with the parameter ‘--nomodel -q 0.01’. The read counts were calculated using featureCounts (v.1.6.3). Motif analysis was conducted using HOMER (v.4.1) (http://homer.ucsd.edu/homer/). Heat maps were generated using deeptools (v.3.2.1)^85^. Binding peaks were visualized in the Integrative Genomics Viewer (v.2.8.13)^86^. The data have been deposited at the DNA Data Bank of Japan (DRA016932).

### SDS–PAGE and immunoblot analysis

SDS–PAGE and immunoblot analysis were performed as described previously^36^. The antibodies used in this analysis were anti-GFP (1,000× dilution; SAB4301138, Sigma), anti-TGG1 (5,000× dilution)^36^, anti-TGG2 (5,000× dilution)^36^, anti-ACTIN (2,000× dilution; A0480, Sigma-Aldrich) and anti-histone H3 (1,000× dilution; AB1791, Abcam). Membranes were exposed to Immobilon Western HRP Substrate (Millipore, WBKL0500), and luminescence signals were detected with a CCD imager (ImageQuant LAS 4000, GE Life Sciences). Stripping of membranes was performed with Restore PLUS Western Blot Stripping Buffer (Thermo Fisher, 46430). SimplyBlue SafeStain (Invitrogen, 465034) was used for Coomassie brilliant blue staining of membranes to ensure equal loading. As molecular weight standards, SeeBlue Plus2 Pre-stained standard (Thermo Fisher) and MagicMark XP Western Protein (Thermo Fisher) were used for SDS–PAGE and immunoblot analysis, respectively. Full images of immunoblots and Coomassie brilliant blue-stained membranes are shown in Supplementary Figs. 1–7.

### Quantification of stomatal features

For confocal microscopy, cell peripheries of the third true leaves at 14 and 21 DAG were visualized following staining with 1 mg ml^−1^ propidium iodide (Molecular Probes, P1304MP). Images were captured using a confocal laser-scanning microscope (Leica SP8 FALCON) with a WLL, a HyD detector and a gating system. Approximately 40 sequential confocal slices were used for the construction of *Z*-stack images covering a 0.308-mm^2^ field. Brightness and contrast were uniformly adjusted using Photoshop 2021 (Adobe). The number of each category of stomata was scored in the 0.308-mm^2^ field (WT *n* = 9, *wsb* *n* = 8, *scap1* *n* = 8, *wsb scap1* *n* = 15, *gWSB-mTurquoise2* *n* = 24, *gWSB-mVenus* *n* = 9, *gSCAP1-mVenus* *n* = 21, *gSCAP1-mCherry* *n* = 13).

### Transmission electron microscopy

The third true leaves of WT and *wsb scap1* seedlings were cut and immediately fixed with 2% (w/v) paraformaldehyde and 2% (w/v) glutaraldehyde in 0.05 M cacodylate buffer (pH 7.4) at 4 °C overnight. After fixation, the samples were washed 3 times with 0.05 M cacodylate buffer for 30 min each and postfixed with 2% (w/v) osmium tetroxide in 0.05 M cacodylate buffer (pH 7.4) at 4°C for 3 h. The samples were infiltrated with propylene oxide (PO) twice for 30 min each, placed into a 1:1 (v/v) mixture of PO and resin (Quetol-651, Nisshin EM) for 3 h, and transferred to 100% resin for 3 h. The samples were allowed to polymerize at 60 °C for 48 h. The polymerized resin blocks were ultrathin sectioned to 80 nm with a diamond knife using an ultramicrotome (Ultracut UCT, Leica) and the sections were mounted onto copper grids. The sections were stained with 2% (w/v) uranyl acetate at room temperature for 15 min and washed with distilled water, followed by secondary staining with 1× lead stain solution (Sigma-Aldrich) at room temperature for 3 min. The grids were observed with a transmission electron microscope (JEM-1400Plus, JEOL) at an acceleration voltage of 100 kV. Digital images (3,296 × 2,472 pixels) were taken with a CCD camera (EM-14830RUBY2, JEOL).

### Phylogenetic analysis

Protein sequences related to Group IV members of the Arabidopsis AP2/ERF TF family^43^ were retrieved from the proteome databases in Phytozome 13 (https://phytozome-next.jgi.doe.gov/) and MarpolBase (https://marchantia.info/)^87^ using BLASTP searches. The amino acid sequences of WSB (At3g57600), At1g75490, At2g38340, At2g40220, At2g40340, At2g40350, At3g11020, At5g05410 and At5g18450 were used as queries for BLASTP searches with a threshold *e*-value of 12. A total of 461 protein sequences were collected from the following 62 plant species (gene name prefixes are given in parentheses): *Amaranthus hypochondriacus* (AH), *Amborella trichopoda* (evm_27.TU.AmTr_v1.0_scaffold), *Anacardium occidentale* (Anaoc.), *Ananas comosus* (Aco), *Anthoceros agrestis* (AagrOXF_evm.model.utg), *Anthoceros angustus* (AANG), *Anthoceros punctatus* (Apun_evm.model.utg), *Aquilegia coerulea* (Aqcoe), *Arabidopsis halleri* (Araha.), *Arabidopsis lyrata* (AL), *Arabidopsis thaliana* (AT), *Asparagus officinalis* (evm.TU.AsparagusV1_), *Beta vulgaris* (EL10Ac), *Brachypodium distachyon* (Bradi), *Brassica oleracea* var. *capitata* (Bol), *Brassica rapa* (Brara.), *Capsella rubella* (Carub.), *Carica papaya* (evm.TU.supercontig), *Ceratodon purpureus* (CepurGG1), *Ceratopteris richardii* (Ceric), *Cinnamomum kanehirae* (CKAN), *Citrus sinensis* (orange1.), *Coffea arabica* (evm.TU.Scaffold), *Corymbia citriodora* (Cocit.), *Dioscorea alata* (Dioal.), *Eucalyptus grandis* (Eucgr.), *Glycine max* (GlymaFiskIII.), *Gossypium hirsutum* (Gohir.), *Helianthus annuus* (HanXRQChr), *Hordeum vulgare* (HORVU.MOREX.r3.), *Kalanchoe laxiflora* (Kalaxd.), *Lactuca sativa* (Lsat_1_v5_gn), *Linum usitatissimum* (Lus), *Lotus japonicus* (Lj), *Manihot esculenta* (Manes.), *Marchantia polymorpha* (Mp), *Medicago truncatula* (Medtr), *Nymphaea colorata* (Nycol.), *Oryza sativa* (Os), *Phaseolus vulgaris* (Pv5-593.), *Physcomitrium patens* (Phpat.), *Picea abies* (MA), *Poncirus trifoliata* (Ptrif.), *Populus trichocarpa* (Potri.), *Ricinus communis* (gene name starts with number), *Salix purpurea* (Sapur), *Salvinia cucullata* (Sacu_v1.1_s), *Setaria italica* (Seita.), *Solanum lycopersicum* (Solyc), *Solanum tuberosum* (Soltu.), *Sorghum bicolor* (SbiSC187.), *Sphagnum fallax* (Sphfalx), *Sphagnum magellanicum* (Sphmag), *Spinacia oleracea* (Spov3_), *Spirodela polyrhiza* (Spipo), *Theobroma cacao* (Thecc.), *Thuja plicata* (Thupl.), *Triticum aestivum* (Traes), *Vitis vinifera* (VIT_), *Vigna unguiculata* (Vigun), *Zea mays* (Zm) and *Zostera marina* (Zosma). A multiple sequence alignment was created using MAFFT (v.7.505) with the ‘--auto’ option (https://mafft.cbrc.jp/alignment/software/)^88^, trimmed with trimAL (v.1.4.rev15) with the ‘-gt 1 -cons 10’ options (http://trimal.cgenomics.org/trimal)^89^ and used for phylogenetic analysis in RAxML (v.8.2.12) with the ‘-f a -m PROTGAMMAAUTO’ options with 1,000 bootstrap replicates (https://cme.h-its.org/exelixis/web/software/raxml/)^90^. The tree was visualized in R (v.4.2.1) with the ggtree package (v.3.4.2)^91^.

### Measurement of stomatal responses to light and CO_2_

Stomatal conductance in intact leaves was measured using a gas-exchange system (LI-6400, Li-Cor) as described previously^92^. Leaves from dark-adapted plants were illuminated with red light (300 µmol m^−2^ s^−1^) for 1 h, and then blue light (10 µmol m^−2^ s^−1^) was superimposed onto red light for 20 min. For the measurement of CO_2_-induced stomatal responses, the ambient CO_2_ concentration was lowered from 350 ppm to 100 ppm and then increased to 800 ppm in the dark. For stomatal aperture measurements, epidermal strips prepared from dark-adapted leaves were incubated in 5 mM MES-bistrispropane (pH 6.5), 50 mM KCl and 0.1 mM CaCl_2_ in the dark or under red light (50 µmol m^−2^ s^−1^) and blue light (10 µmol m^−2^ s^−1^) for 2 h at 24 °C. Stomatal apertures on the abaxial epidermis were measured using an inverted microscope (Eclipse TS100, Nikon).

### Statistics and reproducibility

Statistical analyses were performed using Microsoft Excel, GraphPad Prism 9 and GraphPad Prism 10. Student’s *t*-tests were used to compare two samples. For comparison of three or more samples, one-way analysis of variance (ANOVA) was used to test for significant interactions. When significant, Tukey–Kramer tests were used for pairwise comparisons. For experiments conducted without statistical analysis (for example, CLSM observation and GUS staining), a minimum of three independent biological replicates were performed to confirm reproducibility. The source data behind the graphs in the paper and all statistical analyses including exact *P* values are provided in Supplementary Data 15.

### Reporting summary

Further information on research design is available in the Nature Portfolio Reporting Summary linked to this article.

## Supplementary information

Supplementary Information Supplementary Figs. 1–21.

Reporting Summary

Supplementary Data 1 List of upregulated and downregulated genes in *iFAMA* at 8 h.

Supplementary Data 2 List of upregulated and downregulated genes in *iFAMA* at 24 h.

Supplementary Data 3 Gene ontology (GO) term enrichment analysis of *iFAMA* differentially expressed genes at 8 h.

Supplementary Data 4 List of upregulated and downregulated genes in *scrm* mutants.

Supplementary Data 5 List of genes overlapping between the four transcriptome datasets.

Supplementary Data 6 Comparison of *iFAMA* transcriptome data between this study and ref. ^20^.

Supplementary Data 7 List of upregulated and downregulated genes in *wsb* mutants.

Supplementary Data 8 WSB regulates the expression of MC-related and GC-related genes.

Supplementary Data 9 List of WSB-binding sites.

Supplementary Data 10 Comparative analysis of WSB ChIP-seq and scRNA-seq data of stomatal lineages.

Supplementary Data 11 Synteny analysis of *WSB* orthologues.

Supplementary Data 12 List of mutants and transgenic lines used in this study.

Supplementary Data 13 List of primers used in this study.

Supplementary Data 14 Methods for the visualization of cell outlines.

Supplementary Data 15 Source data for the graphs in the paper and all statistical analyses including exact *P* values.

## Data Availability

The accession numbers for the RNA-seq and ChIP-seq data generated in this study and deposited in the DDBJ database are DRA013687 and DRA016932, respectively. Sequence data from this study can be found in the GenBank/EMBL data libraries under the following accession numbers: *BIM1* (At5g08130), *CCS52A1* (At4g22910), *CYCD5* (At4g37630), *CYCD7* (At5g02110), *EPF1* (At2g20875), *FAMA* (At3g24140), *HIPP20* (At1g71050), *KAT1* (At5g46240), *MYB60* (At1g08810), *POLAR* (At4g31805), *RCI2A* (At3g05880), *SCAP1* (At5g65590), *SCRM* (At3g26744), *SCRM2* (At1g12860), *SHV3* (At4g26690), *SVL1* (At5g55480), *SYP22* (At5g46860), *TA3* (At1g37110), *TET4* (At5g60220), *TGG1* (At5g26000), *TGG2* (At5g25980), *TMM* (At1g80080), *UBQ11* (At4g05050), *VSR1* (At3g52850), *WRKY23* (At2g47260) and *WSB* (At3g57600).
