# Supplementary material for: Co-option and neofunctionalization of stomatal executors for defence against herbivores in Brassicales
Source: Nat Plants. 2025 Feb 24;11(3):483–504. doi: 10.1038/s41477-025-01921-1 (PMC11928322; doi:10.1038/s41477-025-01921-1)
Supplement: Supplementary file 1 — Supplementary Figs. 1–21. [file 41477_2025_1921_MOESM1_ESM.pdf]

nature plants

Supplementary information https://doi.org/10.1038/s41477-025-01921-1

Co-option and neofunctionalization of stomatal executors for defence against herbivores in Brassicales

In the format provided by the authors and unedited

Supplementary Fig. 1

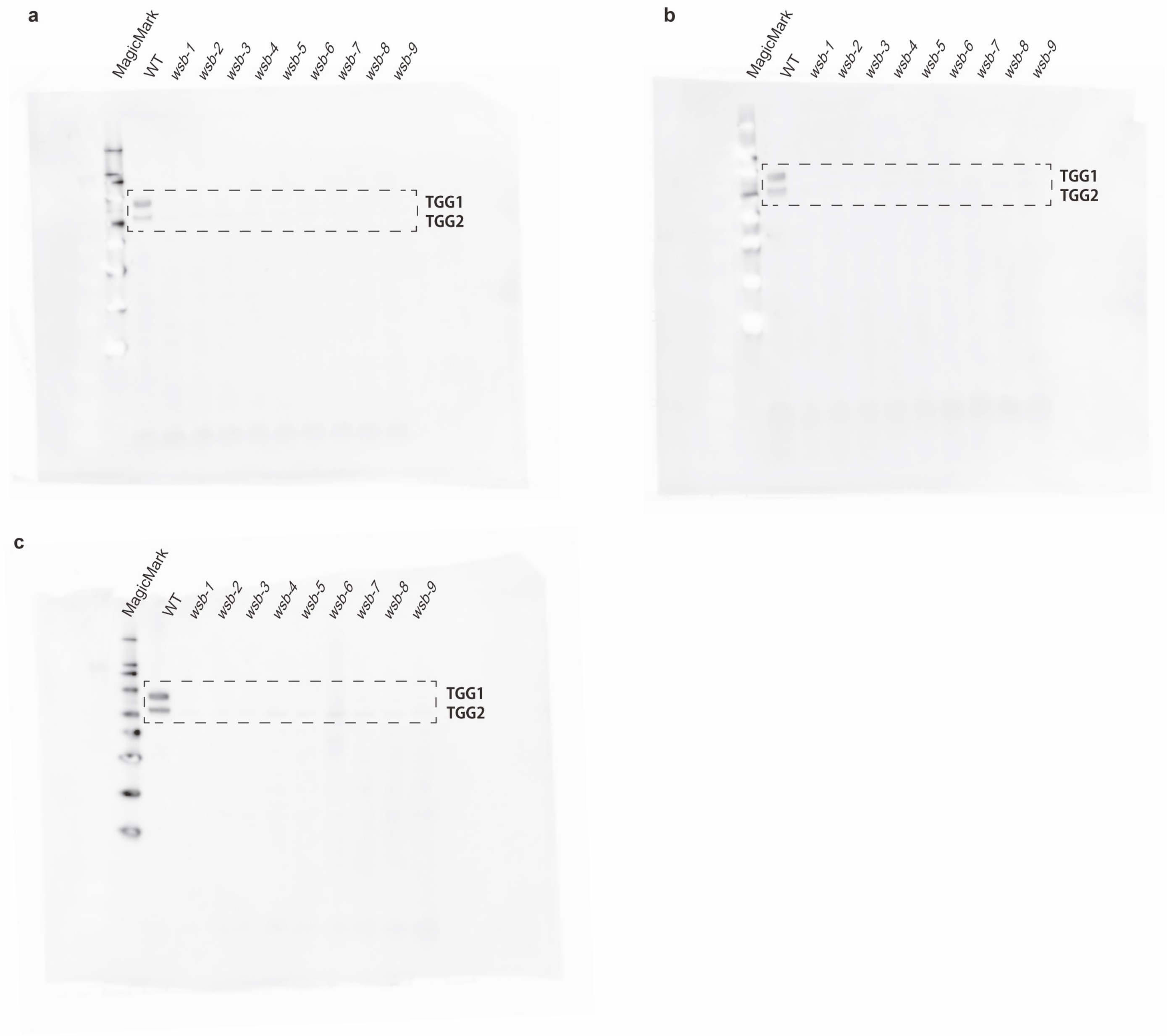

Supplementary Fig. 1| Full images of immunoblots using anti-TGG1 and anti-TGG2 antibodies.

A full image of immunoblots shown in Fig. 3e and Ex. Fig. 5d and two additional replicates of immunoblots. Signals were quantified (all values are given in Supplementary Table 14) and used for plotting graphs in Ex. Fig. 5e,f. MagicMark (MagicMark XP Western Protein Standard, Thermo Fisher Scientific) was used as the molecular size marker.

Supplementary Fig. 2

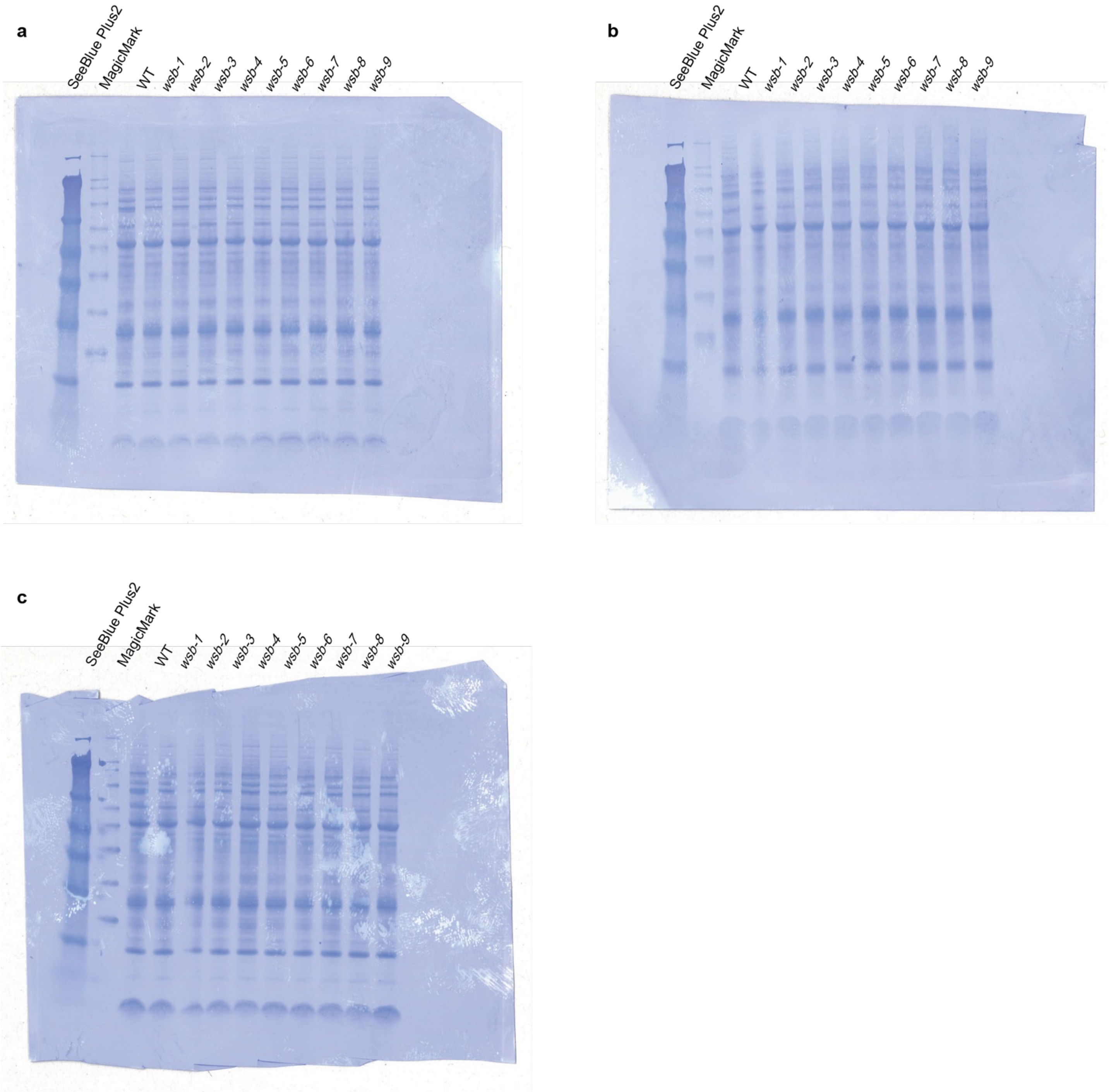

Supplementary Fig. 2| Full images of Coomassie-stained membranes for total proteins as a loading control.

Full images of Coomassie brilliant blue (CBB) staining of membranes used in Supplementary Fig. 1. MagicMark (MagicMark XP Western Protein Standard, Thermo Fisher Scientific) and SeeBlue Plus2 pre-stained standard (Thermo Fisher Scientific) were the molecular size markers.

Supplementary Fig. 3

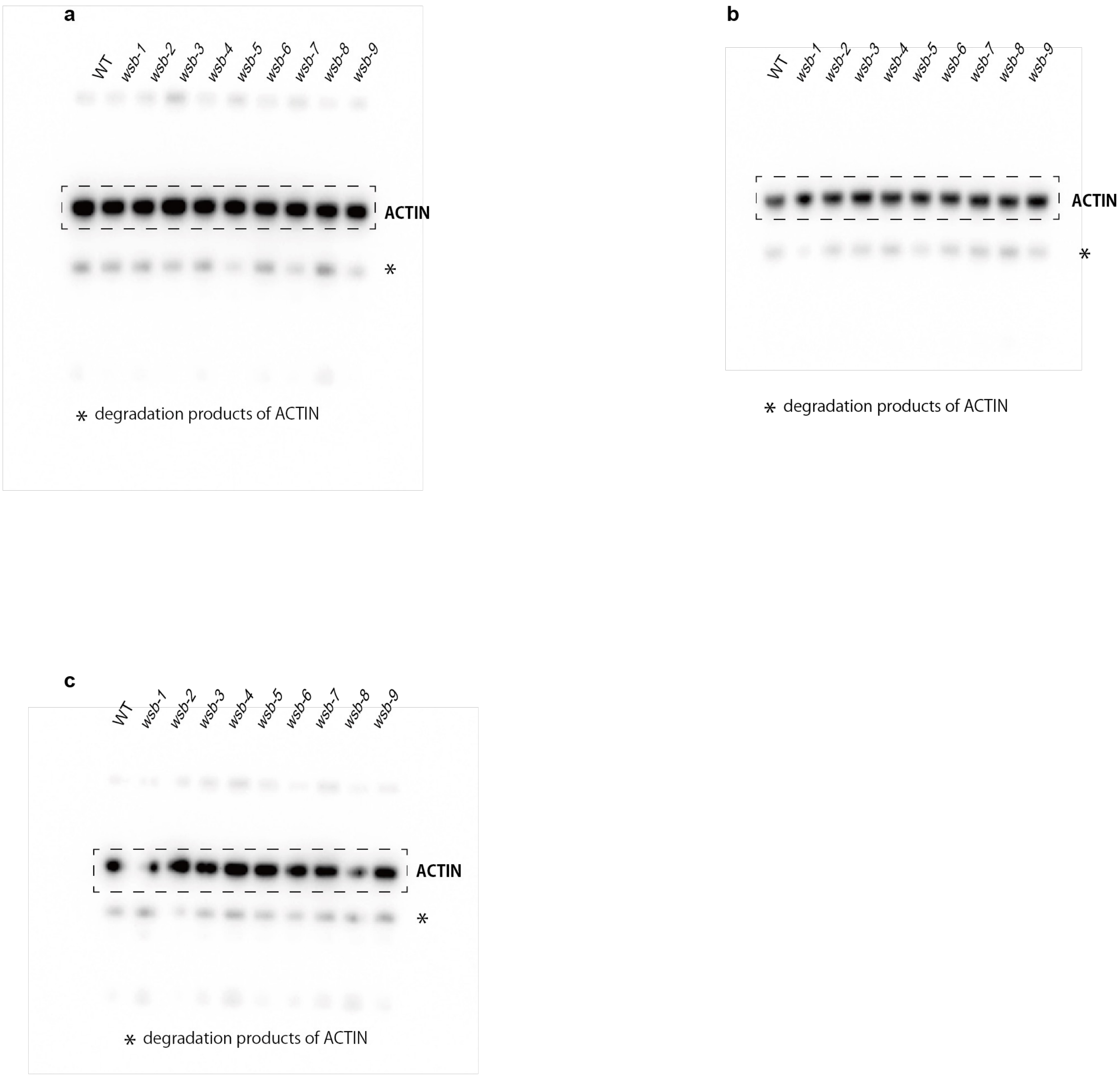

Supplementary Fig. 3| Full images of immunoblots using anti-ACTIN antibodies as a loading control.

Immunoblots were performed using the same membranes used in Supplementary Fig. 1 after stripping and re-blocking. Asterisks indicate possible degradation products of ACTIN.

Supplementary Fig. 4

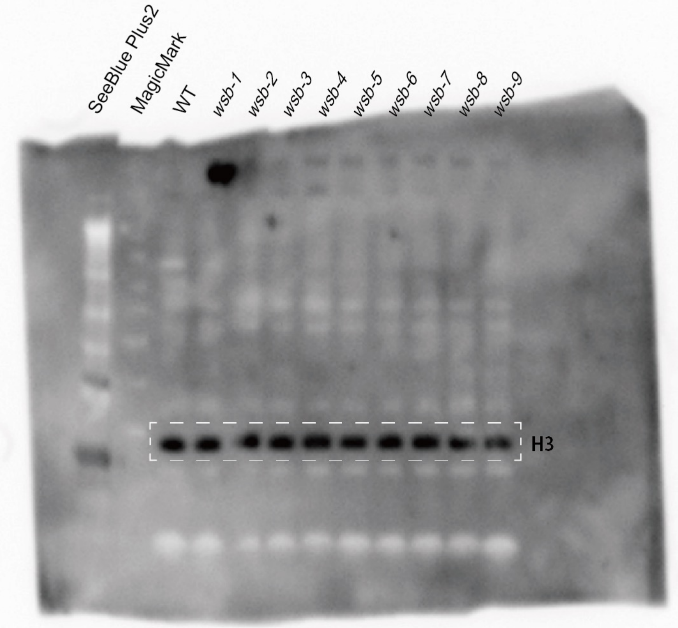

Supplementary Fig. 4| Full images of immunoblots using anti-H3 antibodies as a loading control.

An immunoblot was performed using the same membrane used in Supplementary Fig. 3c after stripping and re-blocking because some scratches were presents in the membrane.

Supplementary Fig. 5

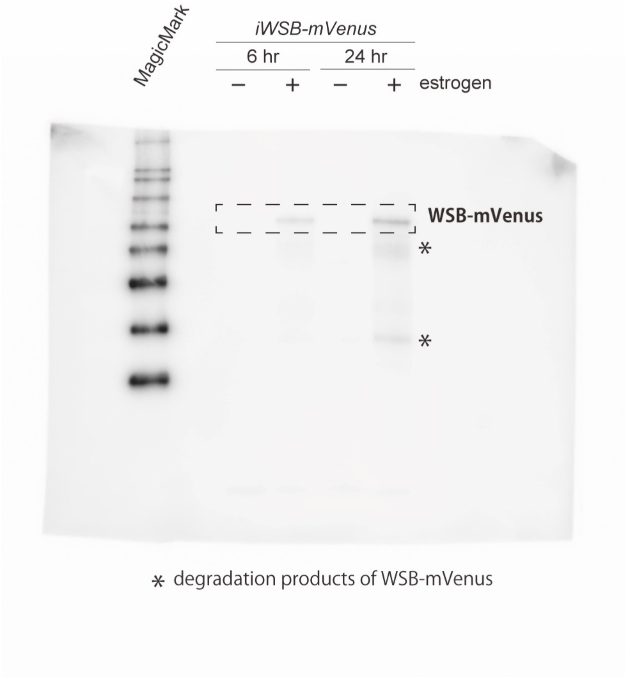

Supplementary Fig. 5| Full images of immunoblots using anti-GFP antibodies.
A full image of the immunoblot shown in Fig. 3j. MagicMark (MagicMark XP Western Protein Standard, Thermo Fisher Scientific) was the molecular size marker.

Supplementary Fig. 6

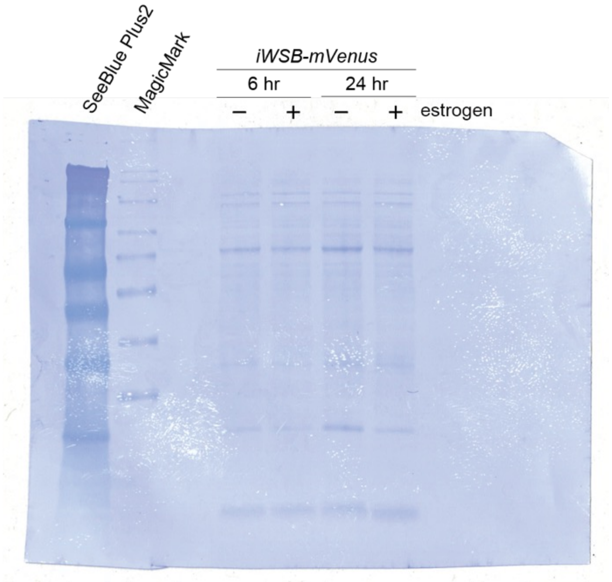

Supplementary Fig. 6| Full images of CBB staining of membranes as a loading control.
A full image of CBB staining of the membrane used in Supplementary Fig. 5. MagicMark (MagicMark XP Western Protein Standard, Thermo Fisher Scientific) and SeeBlue Plus2 Pre-stained standard (Thermo Fisher Scientific) were the molecular size markers.

Supplementary Fig. 7

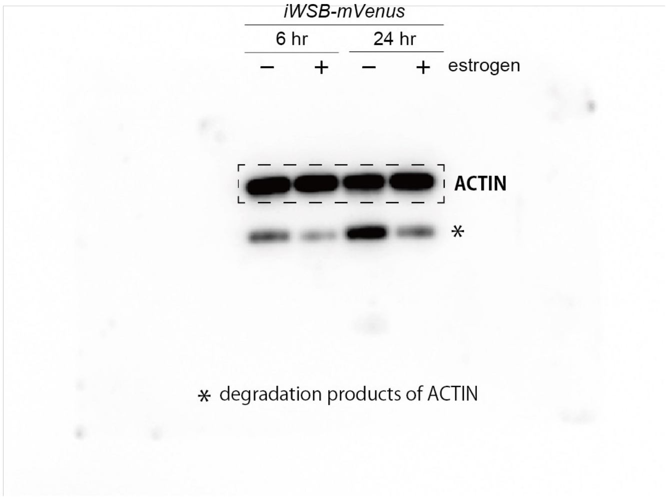

Supplementary Fig. 7| Full images of immunoblots using anti-ACTIN antibodies as a loading control.

Immunoblots were performed using the same membranes used in Supplementary Fig. 5 after stripping and re-blocking. Asterisks indicate possible degradation products of ACTIN.

Supplementary Fig. 8

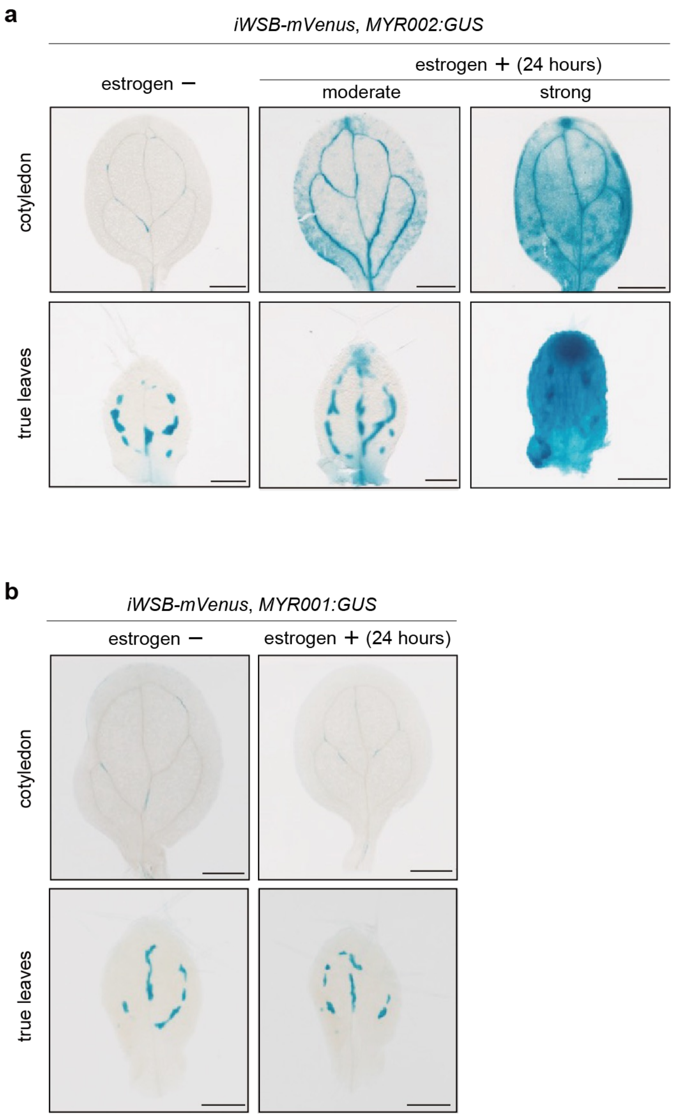

Supplementary Fig. 8| The transient overexpression of *WSB-mVenus* induces the ectopic expression of *MYR002* and does not induce that of *MYR001*.

a, Cells ectopically expressing *MYR002* are induced by the transient overexpression of *WSB-mVenus*. The phenotypes of cotyledons (upper) and true leaves (lower) of *iWSB-mVenus* seedlings at 4 DAG with or without 24 h of estrogen treatment (10 µM) are shown. Scale bars, 500 µm (cotyledon); 100 µm (true leaves).

b, Ectopic expression of *MYR001* is not induced by the transient overexpression of *WSB-mVenus*. The phenotypes of cotyledons (upper) and true leaves (lower) of *iWSB-mVenus* seedlings at 4 DAG with or without 24 h of estrogen treatment (10 µM) are shown. Scale bars, 500 µm (cotyledon); 100 µm (true leaves).

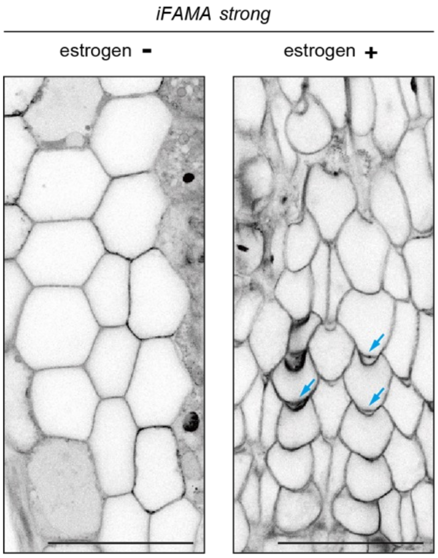

Supplementary Fig. 9| Epidermal phenotypes of hypocotyls for *iFAMA* overexpression lines.

Formation of ectopic guard cells is induced by the overexpression of *FAMA*. *iFAMA*^{strong} transgenic seedlings were cultivated in estrogen (10 μM)-containing medium for 15 days from seeds. Plasma membranes of hypocotyls cells were stained with propidium iodide. The confocal images were converted to grayscale. Arrows indicate ectopic pores.

Scale bars, 100 μm.

Supplementary Fig. 10

gSCAP1-mVenus (green)
SR2200 (magenta)

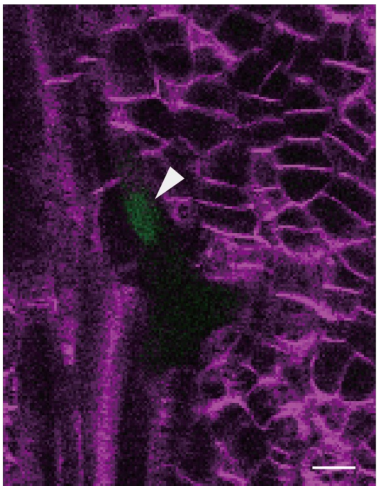

Supplementary Fig. 10| Expression *SCAP1* in myrosin cell lineages with very low frequency.
Confocal image of inner tissues from a *scap1 gSCAP1-mVenus* true leaf at 8 DAG. mVenus signal is shown in green, and the signal from the dye SR2200 (cell wall) is shown in magenta. Note that mVenus signal in myrosin cell lineages is very rare in *scap1 gSCAP1-mVenus*.
Scale bar, 10 μm.

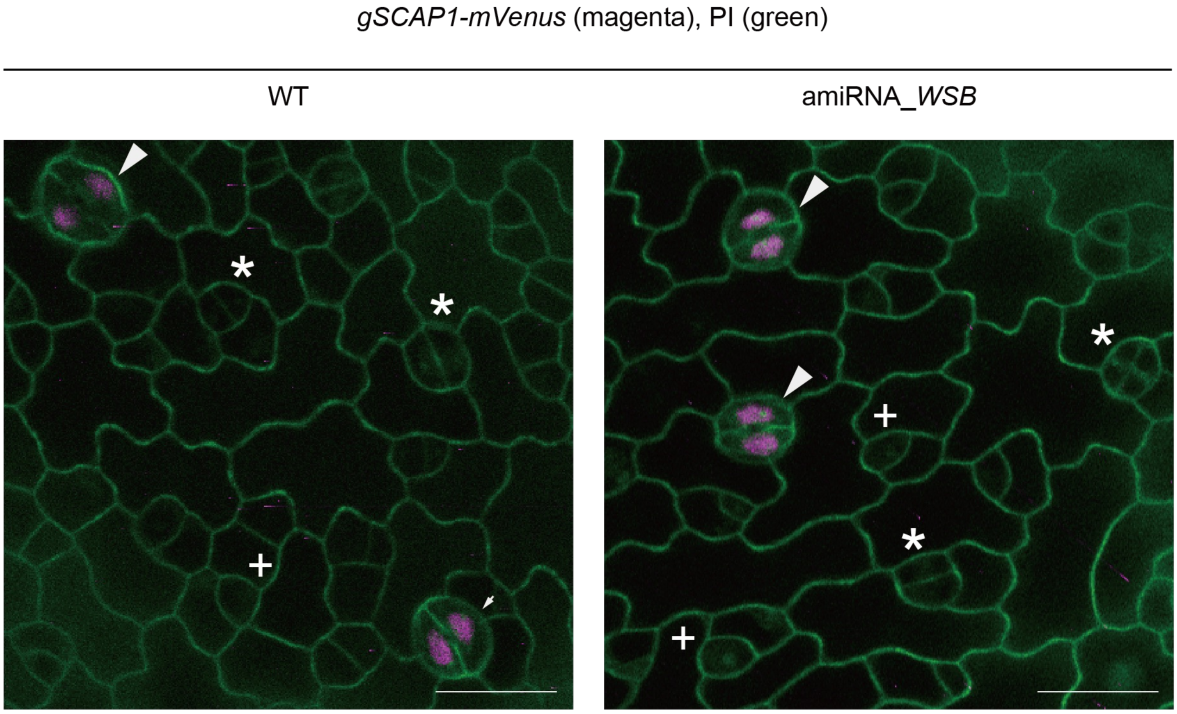

Supplementary Fig. 11| The expression window of *SCAP1* in stomatal lineages does not change in *WSB*-knockdown lines (amiRNA_*WSB*).

Confocal images of cotyledon epidermis from seedlings at 5 DAG from *scap1 gSCAP1-mVenus* (left) and *scap1 gSCAP1-mVenus* amiRNA-*WSB* (right). SCAP1-mVenus is shown in magenta. Cell walls were stained with propidium iodide (green). Arrowheads, GCs; asterisks, GCs just after division; plus, late meristemoids. Note that the expression window of *SCAP1* in the stomatal lineage in *WSB*-knockdown lines was comparable to that in the WT. Scale bars, 20 µm.

Supplementary Fig. 12

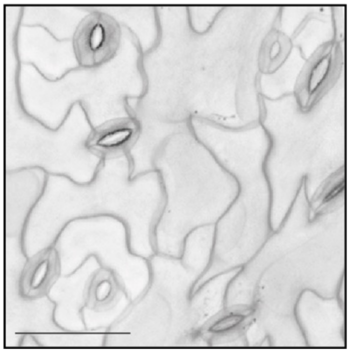

Supplementary Fig. 12| *gSCAP1-mTurquoise2* expression rescues the stomatal phenotypes of *wsb scap1*.
Cell walls were stained with propidium iodide. Confocal image (converted to grayscale) showing the abaxial epidermis from the true leaves of *wsb scap1 gSCAP1-mTurquoise2* at 19 DAG. Note that *gSCAP1-mTurquoise2* rescued the stomatal phenotypes of *wsb scap1*, suggesting that SCAP1-mTurquoise2 is functional. Scale bar, 50 µm

Supplementary Fig. 13

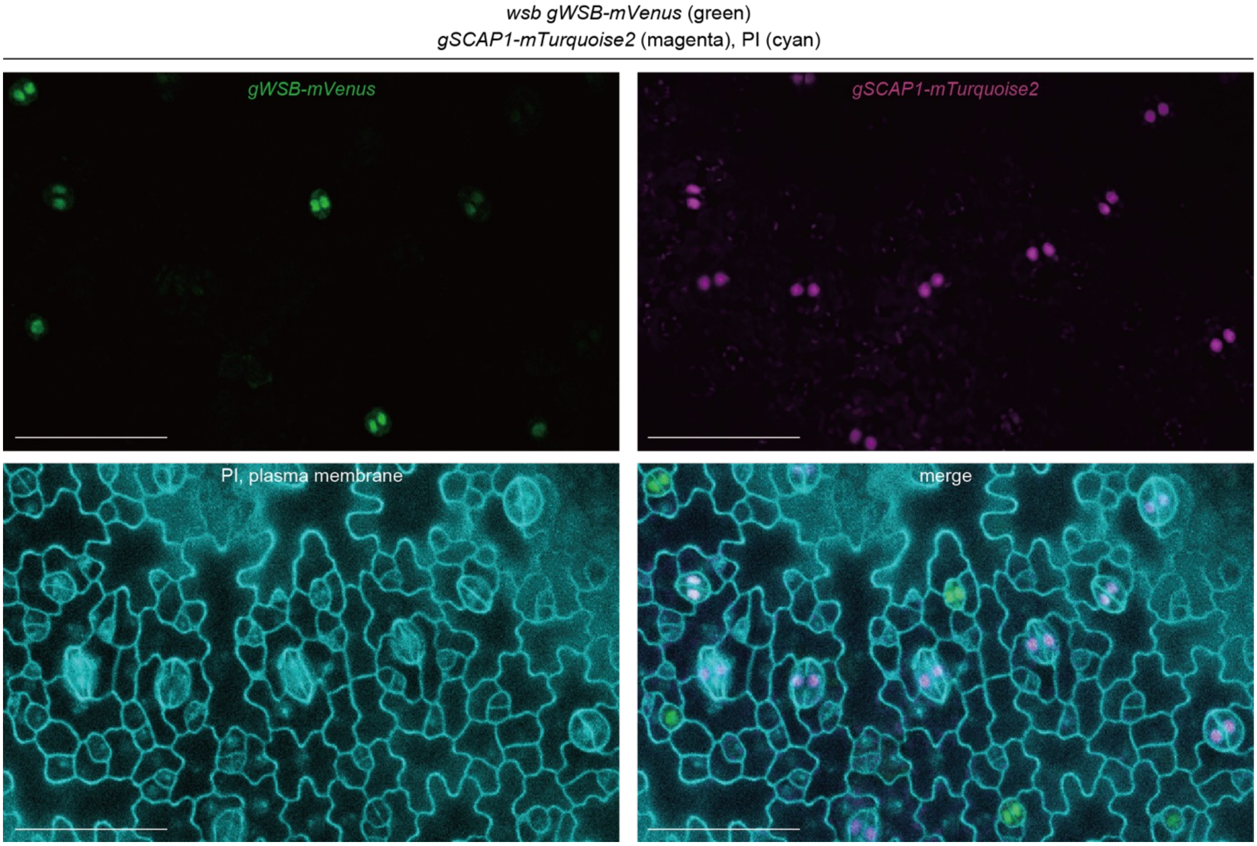

Supplementary Fig. 13| Confocal images of each and merged channels for Fig. 4g.
Confocal image showing the abaxial epidermis from the sixth true leaf of *wsb gWSB-mVenus gSCAP1-mTurquoise2* seedlings at 10 DAG.WSB-mVenus, SCAP1-mTurquoise2, and cell walls (propidium iodide) are shown in green, magenta, and cyan, respectively.
Scale bar, 50 µm

Supplementary Fig. 14

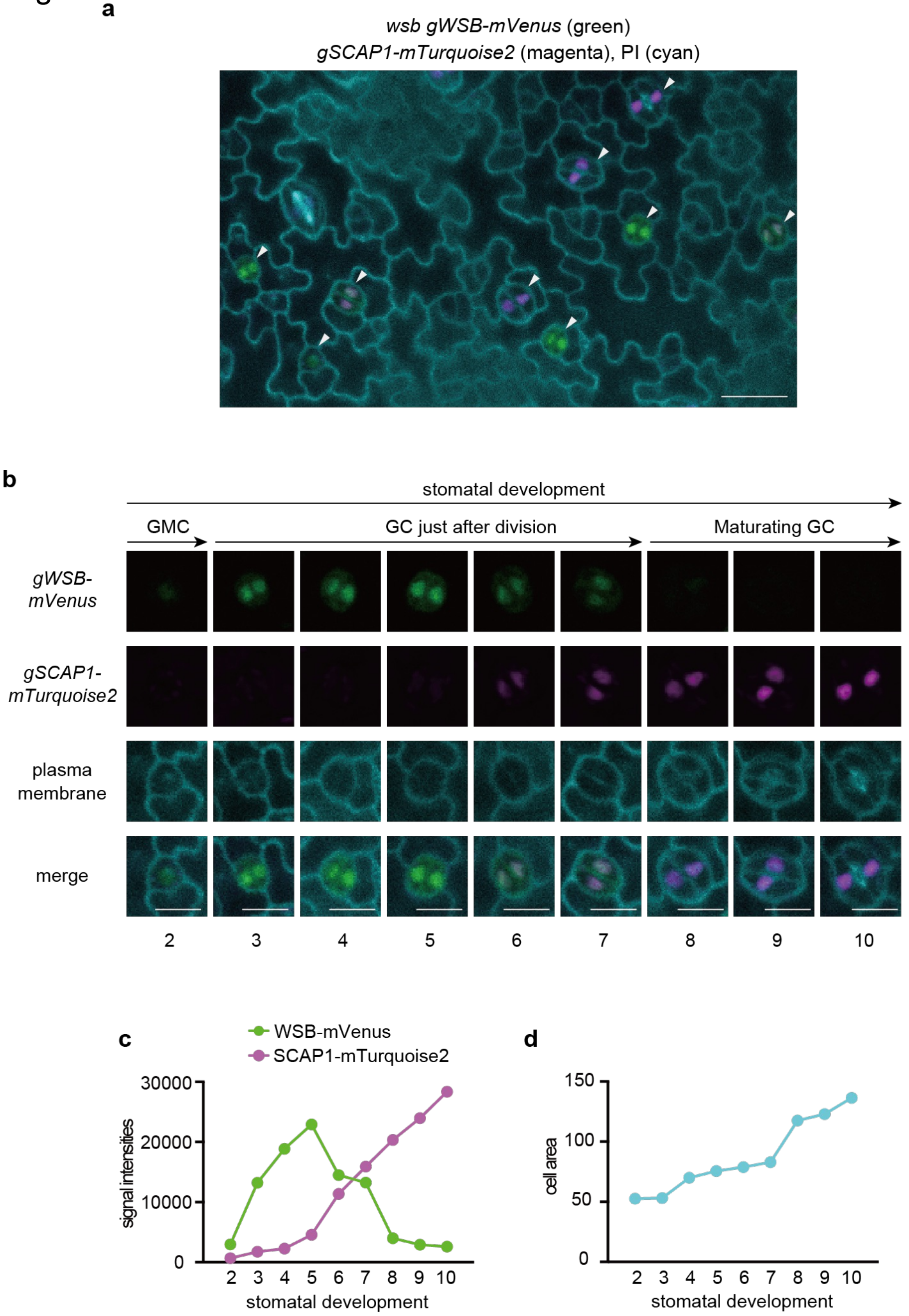

Supplementary Fig. 14| Confocal image and analysis of *gWSB-mVenus gSCAP1-mTurquoise2*

a, Confocal images of the abaxial epidermis from the fifth true leaves of *wsb gWSB-mVenus gSCAP1-mTurquoise2* seedlings at 10 DAG (WSB-mVenus and SCAP1-mTurquoise2 are shown in green and magenta, respectively). Cell walls were stained with propidium iodide (cyan). Stomatal lineage cells indicated by arrowheads are enlarged in (**b**).

b, Confocal images of each and merged channels of nine GCs indicated by arrowheads in (**a**). GCs were arranged as a function of stomatal development, which was estimated by GC sizes. Note that WSB-mVenus and SCAP1-mTurquoise2 co-accumulate at stages 6 and 7.

c, Quantification of fluorescent signals for WSB-mVenus (green) and SCAP1-mTurquoise2 (magenta) through stomatal development using LAS X quantification mode.

d, Quantification of cell area of GCs through stomatal development using LAS X quantification mode.

Scale bars, 20 μm (**a**); 10 μm (**b**).

Supplementary Fig. 15

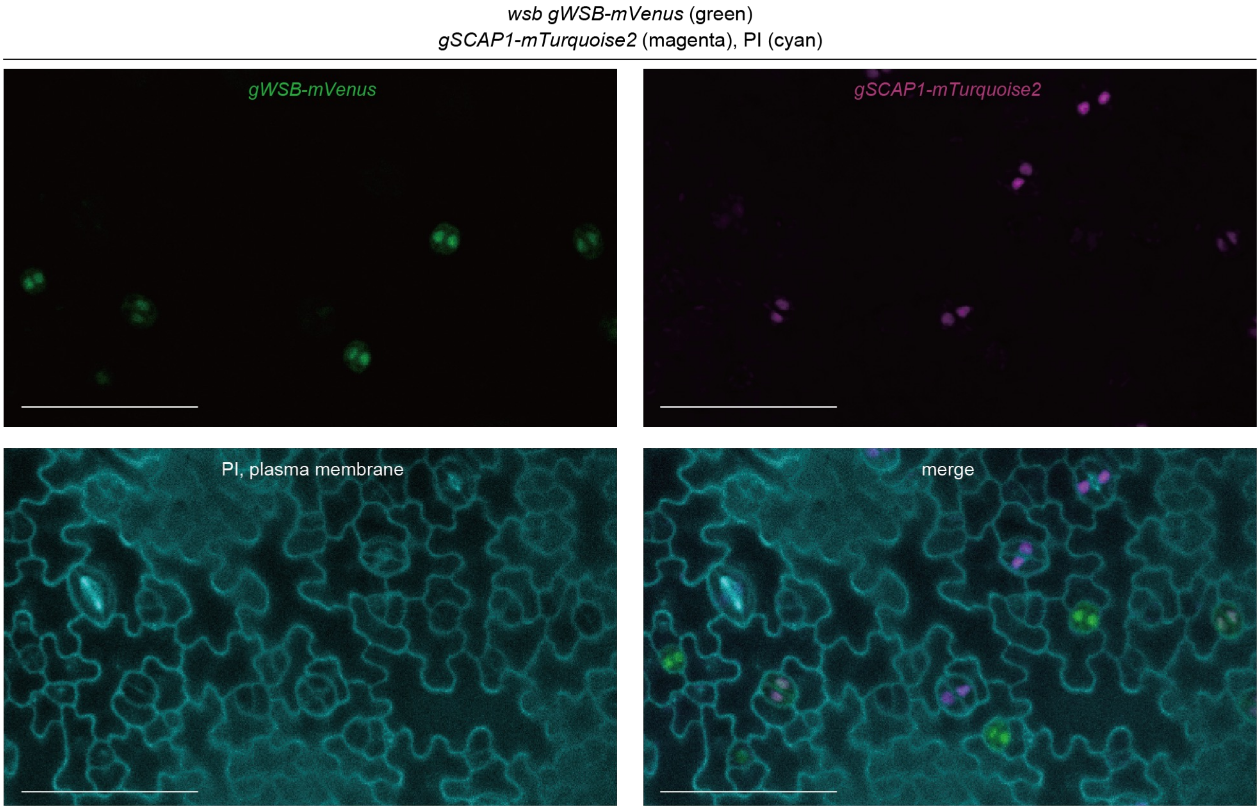

Supplementary Fig. 15| Confocal images of each and merged channels for Supplementary Fig. 14.

Confocal image showing the abaxial epidermis from the fifth true leaves of *wsb gWSB-mVenus gSCAP1-mTurquoise2* seedling at 10 DAG. WSB-mVenus, SCAP1-mTurquoise2, and cell walls (propidium iodide) are shown in green, magenta, and cyan, respectively.
Scale bar, 50 µm.

wsb scap1 (21 DAG)

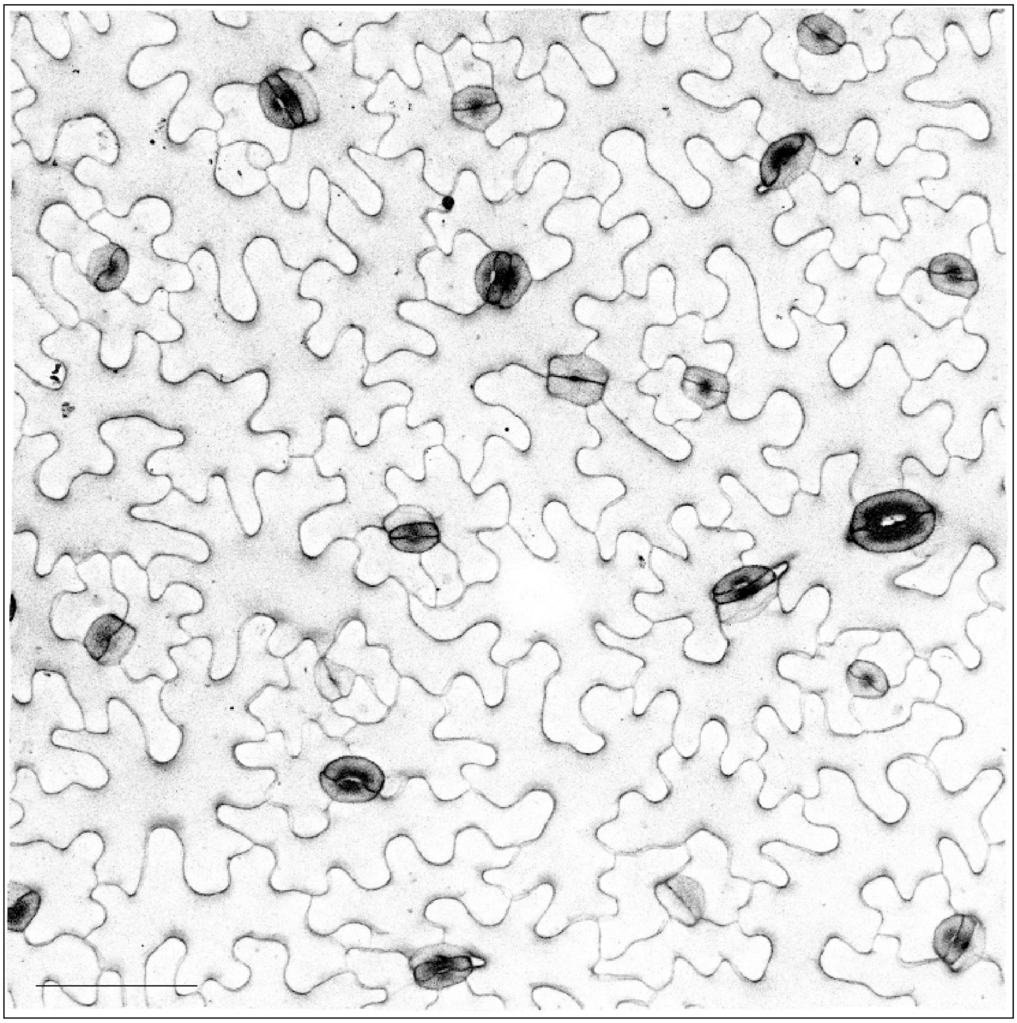

Supplementary Fig. 16| Stomatal phenotypes of *wsb scap1* at a later time point.
Cell walls were stained with propidium iodide. Approximately 40 sequential confocal slices were used for the reconstruction of Z-stack images covering the 0.308-mm^2 field. Confocal images (converted to grayscale) showing the abaxial epidermis from the third true leaf of seedlings at 21 DAG from *wsb scap1* are shown. Scale bars, 50 µm

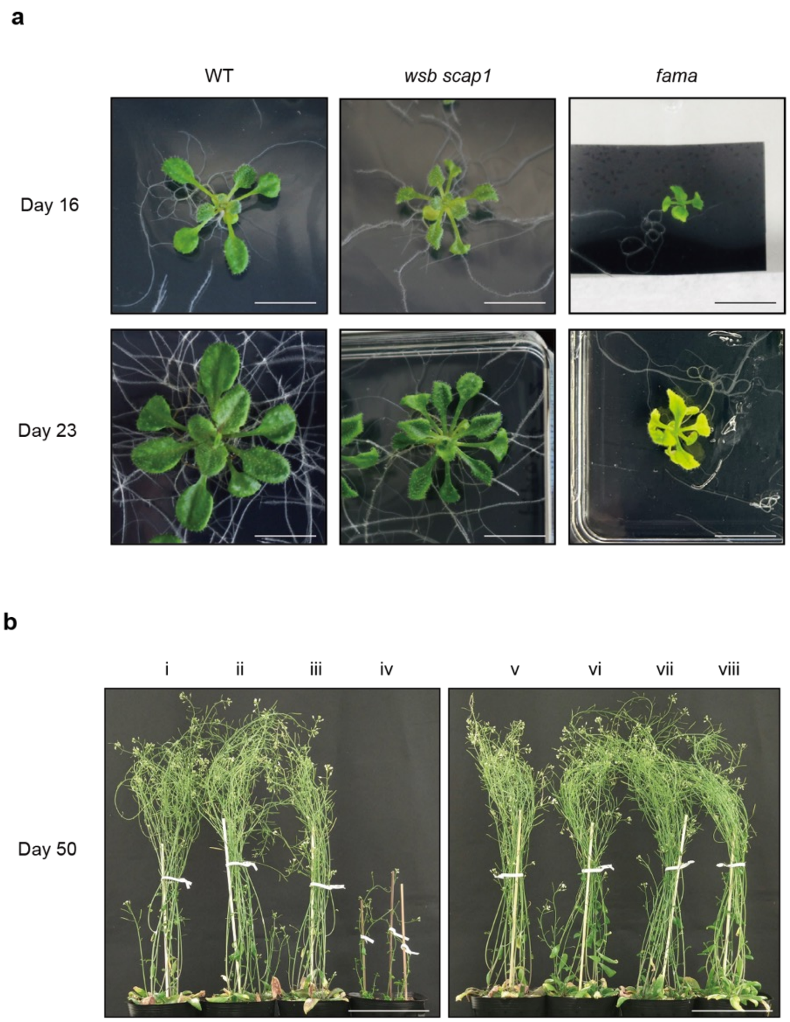

Supplementary Fig. 17| *wsb scap1* plants have a dwarf phenotype.
a, Upper, the development of WT, *wsb scap1*, and *fama* at 16 DAG. Lower, the development of WT, *wsb scap1*, and *fama* at 23 DAG. Scale bars, 1 cm.
b, The development of the indicated genotypes at 50 DAG. i, WT; ii, *wsb*; iii, *scap1*; iv, *wsb scap1*; v, *wsb scap1 gWSB-mTurquoise2*; vi, *wsb scap1 gWSB-mVenus*; vii, *wsb scap1 gSCAP1-mVenus*; viii, *wsb scap1 gSCAP1-mCherry*. Scale bars, 10 cm.

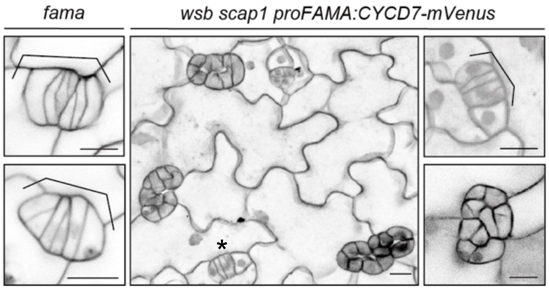

Supplementary Fig. 18| Overexpression of *CYCD7* generates additional divisions in the abnormal GCs of *wsb scap1*.

Cell walls were stained with propidium iodide. Confocal images (converted to grayscale) showing the abaxial epidermis from third true leaves at 14 DAG of *fama* (left) and *wsb scap1 proFAMA:CYCD7-mVenus* (right). *fama* tumors (black brackets in left) were observed only in *fama*. Clusters of undifferentiated stomatal lineage cells (enlarged in lower right) were observed in *wsb scap1 proFAMA:CYCD7-mVenus*.

Scale bars, 10 µm.

Supplementary Fig. 19

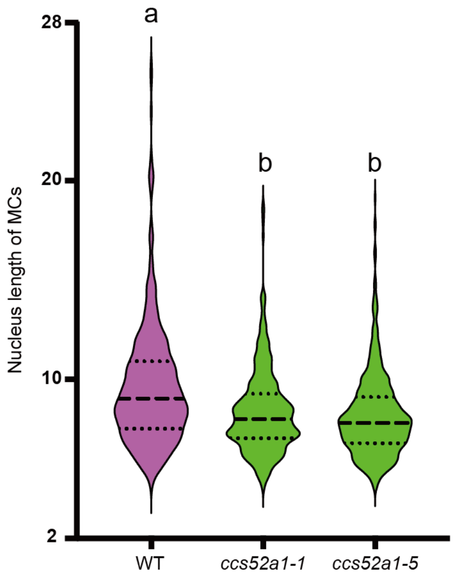

Supplementary Fig. 19| Nucleus length in the WT and *ccs52a1* mutants.
In Fig.6o, many circles from each individual data point masked the lines for the median, first quartile (25th percentile), and third quartile (75th percentile). The graph in Fig6o is reproduced here without the individual data points. Different lowercase letters indicate significant differences. An ANOVA test followed by Tukey-Kramer test was used to determine statistical significance ($p < 0.05$).

Supplementary Fig. 20

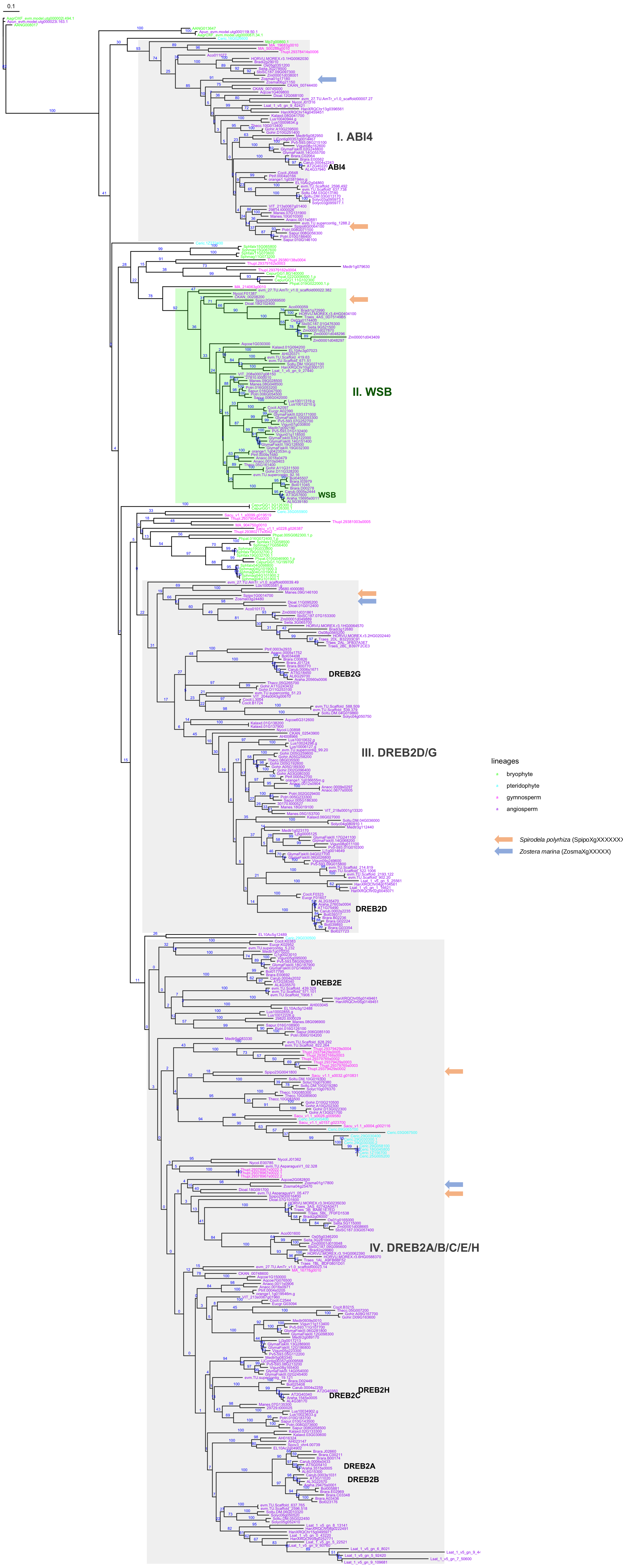

Supplementary Fig. 21

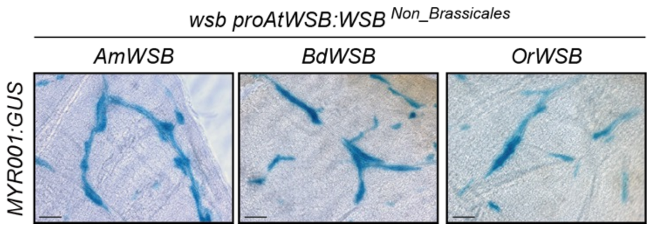

Supplementary Fig. 21| The expression of any one of three *WSB* homologs rescues the loss of MC development seen in *wsb*.

The expression of any one of three *WSB* homologs outside Brassicales rescues the loss of MC development seen in *wsb-3*. Each *WSB* homolog was driven by the *AtWSB* promoter in *wsb* harboring the myrosin cell reporter *MYR001:GUS* (blue). True leaves of transgenic seedlings at 14 DAG were used for GUS staining. At least two independent T2 transgenic lines were analyzed.

Scale bars, 50 µm.
