# Supplementary material for: Co-option and neofunctionalization of stomatal executors for defence against herbivores in Brassicales
Source: Nat Plants. 2025 Feb 24;11(3):483–504. doi: 10.1038/s41477-025-01921-1 (PMC11928322; doi:10.1038/s41477-025-01921-1)
Supplement: Supplementary file 2 — Reporting Summary [file 41477_2025_1921_MOESM2_ESM.pdf]

nature portfolio

Corresponding author(s): SHIRAKAWA, Makoto and ITO, Toshiro.

Last updated by author(s): Dec 28, 2024

Reporting Summary

Nature Portfolio wishes to improve the reproducibility of the work that we publish. This form provides structure for consistency and transparency in reporting. For further information on Nature Portfolio policies, see our Editorial Policies and the Editorial Policy Checklist.

Statistics

For all statistical analyses, confirm that the following items are present in the figure legend, table legend, main text, or Methods section.

n/a	Confirmed	
☐	☒	The exact sample size (*n*) for each experimental group/condition, given as a discrete number and unit of measurement
☐	☒	A statement on whether measurements were taken from distinct samples or whether the same sample was measured repeatedly
☐	☒	The statistical test(s) used AND whether they are one- or two-sided<br>*Only common tests should be described solely by name; describe more complex techniques in the Methods section.*
☒	☐	A description of all covariates tested
☐	☒	A description of any assumptions or corrections, such as tests of normality and adjustment for multiple comparisons
☐	☒	A full description of the statistical parameters including central tendency (e.g. means) or other basic estimates (e.g. regression coefficient) AND variation (e.g. standard deviation) or associated estimates of uncertainty (e.g. confidence intervals)
☐	☒	For null hypothesis testing, the test statistic (e.g. F, t, r) with confidence intervals, effect sizes, degrees of freedom and P value noted<br>*Give P values as exact values whenever suitable.*
☒	☐	For Bayesian analysis, information on the choice of priors and Markov chain Monte Carlo settings
☒	☐	For hierarchical and complex designs, identification of the appropriate level for tests and full reporting of outcomes
☒	☐	Estimates of effect sizes (e.g. Cohen's *d*, Pearson's *r*), indicating how they were calculated

Our web collection on statistics for biologists contains articles on many of the points above.

Software and code

Policy information about availability of computer code

Data collection	All software used (including the version and parameter, when not default) is indicated in the Methods section.
Data analysis	All software used (including the version and parameter, when not default) is indicated in the Methods section.

For manuscripts utilizing custom algorithms or software that are central to the research but not yet described in published literature, software must be made available to editors and reviewers. We strongly encourage code deposition in a community repository (e.g. GitHub). See the Nature Portfolio guidelines for submitting code & software for further information.

Data

Policy information about availability of data

All manuscripts must include a data availability statement. This statement should provide the following information, where applicable:

- Accession codes, unique identifiers, or web links for publicly available datasets
- A description of any restrictions on data availability
- For clinical datasets or third party data, please ensure that the statement adheres to our policy

Data supporting the findings of this work are available within the paper (and its supplementary information files). The datasets, plant materials, and mathematical programing language-written source code files are available from corresponding authors upon request.

Research involving human participants, their data, or biological material

Policy information about studies with human participants or human data. See also policy information about sex, gender (identity/presentation), and sexual orientation and race, ethnicity and racism.

Reporting on sex and gender	n/a
Reporting on race, ethnicity, or other socially relevant groupings	n/a
Population characteristics	n/a
Recruitment	n/a
Ethics oversight	n/a

Note that full information on the approval of the study protocol must also be provided in the manuscript.

Field-specific reporting

Please select the one below that is the best fit for your research. If you are not sure, read the appropriate sections before making your selection.

☒ Life sciences ☐ Behavioural & social sciences ☐ Ecological, evolutionary & environmental sciences

For a reference copy of the document with all sections, see nature.com/documents/nr-reporting-summary-flat.pdf

Life sciences study design

All studies must disclose on these points even when the disclosure is negative.

Sample size	Sample sizes were determined based on prior experience and typical standards in the field (Furuta et al., Nature Communications. 2024; Yamaguchi et al., 2021. Nature Communications; Vatén et al., 2018. Developmental Cell.). For statistical tests (i.e. Student's t-test, Tukey-Kramer test), at least 3 biological replicates were included to ensure enough sample size.
Data exclusions	Only experimental group and control group were included in the analyses. Any plants without these traits were excluded by phenotyping and/or genotyping.
Replication	All experiments were repeated at least three times, and attempts at replication were successful.
Randomization	Random selection was not conducted. Plants in either experimental group or control group were included in the study. These two groups were distinct from each other; experimental group was compared with control group for phenotyping/expression.
Blinding	Blinding was not applied because the experimental and control groups were clearly distinct in terms of their appearance and treatment, making blinding unnecessary. Furthermore, the potential for this distinction to introduce errors or biases was deemed to be minimal. However, we carefully evaluated the risk of any influence on data interpretation due to the lack of blinding and implemented additional experimental controls to mitigate such risks.

Reporting for specific materials, systems and methods

We require information from authors about some types of materials, experimental systems and methods used in many studies. Here, indicate whether each material, system or method listed is relevant to your study. If you are not sure if a list item applies to your research, read the appropriate section before selecting a response.

Materials & experimental systems

n/a	Involved in the study	
☐	☒	Antibodies
☒	☐	Eukaryotic cell lines
☒	☐	Palaeontology and archaeology
☒	☐	Animals and other organisms
☒	☐	Clinical data
☒	☐	Dual use research of concern
☐	☒	Plants

Methods

n/a	Involved in the study	
☐	☒	ChIP-seq
☒	☐	Flow cytometry
☒	☐	MRI-based neuroimaging

Antibodies

Antibodies used	Anti-Myc (300x dilution, sc-40 X; Santa Cruz Biotechnology)<br>anti-GFP (200x dilution for ChIP-qPCR and 1000x dilution for ChIP-seq, SAB4301138, SIGMA)<br>anti-TGG1 (5,000x dilution, previously described in Ueda et al., 2006. Plant and Cell Physiology)<br>anti-TGG2 (5,000x dilution, previously described in Ueda et al., 2006. Plant and Cell Physiology)<br>anti-ACTIN (2,000x dilution, A0480, Sigma-Aldrich)<br>Anti-histone H3 (1,000x dilution, AB1791, Abcam)
Validation	The following antibodies were validated by the suppliers.<br>https://www.scbt.com/p/c-myc-antibody-9e10<br>https://www.sigmaaldrich.com/JP/ja/product/sigma/sab4301138<br>Anti-TGG1 and anti-TGG2 were previously validated by Ueda et al. (Ueda et al., 2006. Plant and Cell Physiology.)<br>https://www.sigmaaldrich.com/JP/ja/product/sigma/a0480<br>https://www.abcam.com/en-us/products/primary-antibodies/histone-h3-antibody-nuclear-marker-and-chip-grade-ab1791

Plants

Seed stocks	All seed stocks used is indicated in the Methods section and Supplementary_table12.
Novel plant genotypes	All seed stocks used is indicated in the Methods section and Supplementary_table12.
Authentication	To confirm authentication of seeds (i.e. to avoid the effects off-target gene editing/mutations), we used independent alleles of mutants, and performed allelism tests and complementation tests.

ChIP-seq

Data deposition

☒ Confirm that both raw and final processed data have been deposited in a public database such as GEO.

☒ Confirm that you have deposited or provided access to graph files (e.g. BED files) for the called peaks.

Data access links<br>*May remain private before publication.*	The data was uploaded onto DDBJ website<br>https://www.ddbj.nig.ac.jp/index.html
Files in database submission	DRA016932
Genome browser session<br>(e.g. UCSC)	no longer applicable

Methodology

Replicates	Replicates agreed well. Data was further confirmed by ChIP-qPCR
Sequencing depth	Sample Name Total reads Trimmed Reads Mapped reads Mapping rate<br>iWSB_es_ChIP 42458162 26716148 23711229 88.75%<br>iWSB_es_Input 48313110 27586052 24932716 90.38%
Antibodies	anti-GFP (SAB4301138, SIGMA)
Peak calling parameters	Peak calling is described in the methods.
Data quality	Peak attributes are described in the methods.
Software	Software used is described in the methods.
